# Discovery of Benzo[*d*]imidazole‐6‐sulfonamides as Bromodomain and Extra‐Terminal Domain (BET) Inhibitors with Selectivity for the First Bromodomain

**DOI:** 10.1002/cmdc.202200343

**Published:** 2022-09-15

**Authors:** Alessandra Cipriano, Ciro Milite, Alessandra Feoli, Monica Viviano, Giacomo Pepe, Pietro Campiglia, Giuliana Sarno, Sarah Picaud, Satomi Imaide, Nikolai Makukhin, Panagis Filippakopoulos, Alessio Ciulli, Sabrina Castellano, Gianluca Sbardella

**Affiliations:** ^1^ Department of Pharmacy University of Salerno via Giovanni Paolo II 132 84084 Fisciano (SA) Italy; ^2^ Nuffield Department of Medicine Oxford University OX3 7DQ Oxford UK; ^3^ Division of Biological Chemistry and Drug Discovery School of Life Sciences University of Dundee Dow Street Dundee DD1 5EH, Scotland UK; ^4^ Discovery Technology Research Laboratories Ono Pharmaceutical Co., Ltd. 618-8585 Osaka Japan; ^5^ Oncology R&D Tumour Targeted Delivery AstraZeneca QMB Innovation Centre 42 New Road London E1 2AX UK

**Keywords:** Benzimidazole-6-sulfonamide, Bromodomain, Bioisosteres, Selectivity, Structure-activity relationships

## Abstract

The bromodomain and extra‐terminal (BET) family of proteins includes BRD2, BRD3, BRD4, and the testis‐specific protein, BRDT, each containing two *N*‐terminal tandem bromodomain (BRD) modules. Potent and selective inhibitors targeting the two bromodomains are required to elucidate their biological role(s), with potential clinical applications. In this study, we designed and synthesized a series of benzimidazole‐6‐sulfonamides starting from the azobenzene compounds MS436 (**7 a**) and MS611 (**7 b**) that exhibited preference for the first (BD1) over the second (BD2) BRD of BET family members. The most‐promising compound (**9 a)** showed good binding potency and improved metabolic stability and selectivity towards BD1 with respect to the parent compounds.

## Graphical abstract

**Bioisosteric replacement** of the azobenzene moiety of selective BET inhibitors with a benzimidazole ring afforded a set of benzimidazole‐6‐sulfonamides. Evaluation of the binding activity against diverse BRD families endorses the benzimidazole as a useful scaffold to obtain compounds with improved selectivity towards the first bromodomains of BET family proteins.

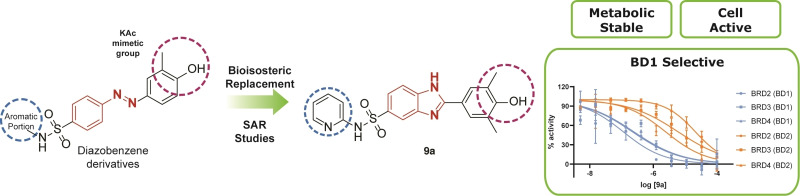

## Introduction

**Figure 1:**
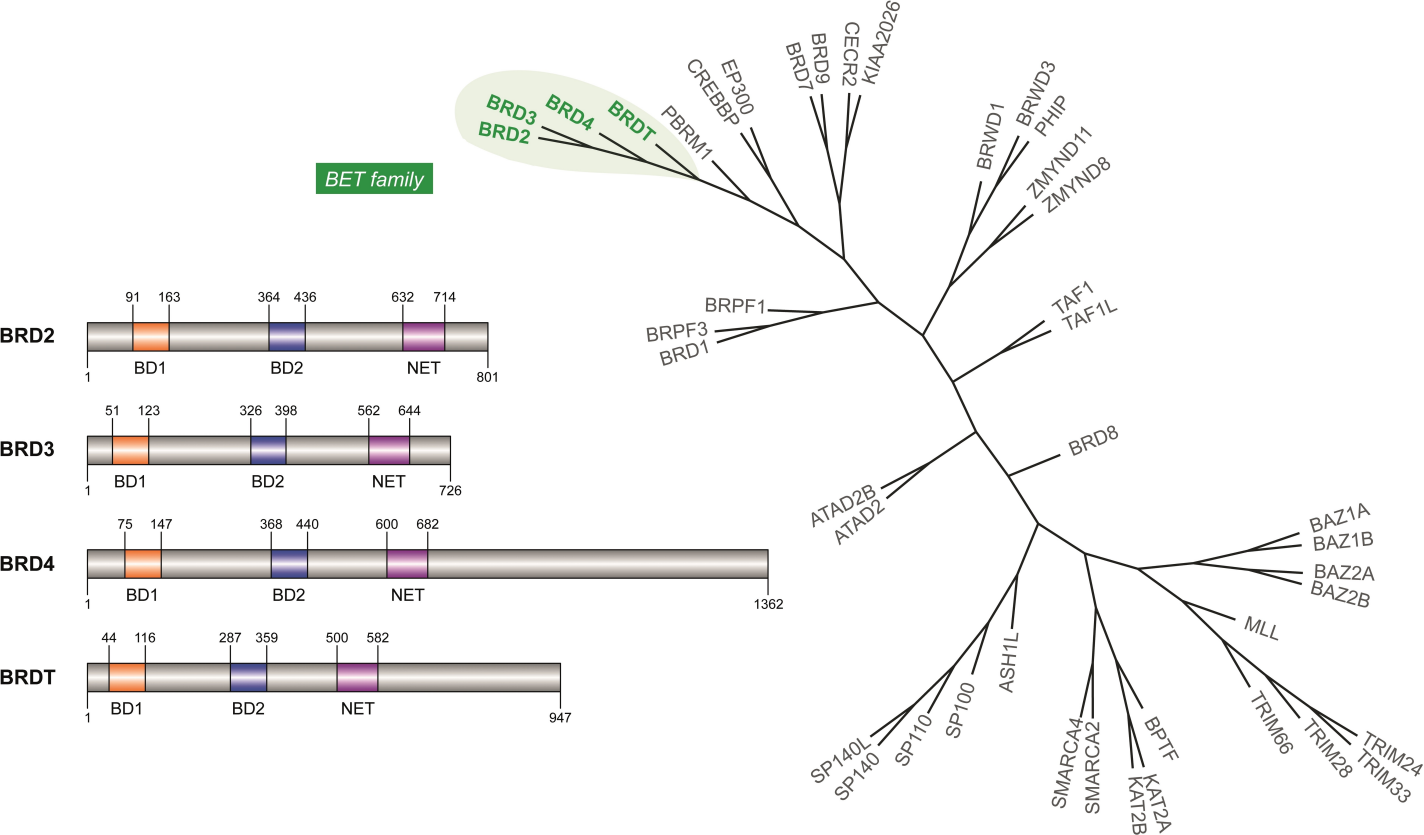
Bromodomain phylogenetic tree and domain structure of BET family.

Among all the proteins able to “read” the epigenetic code, bromodomains (BRDs) are specialised protein domains responsible for the recognition and binding of N‐acetyl lysine residues (KAc) on histone and non‐histone proteins. The human proteome encodes 61 different BRDs belonging to 42 proteins.^[1]^ Among these, the bromodomain and extra terminal (BET) family has been extensively studied, establishing a central role in transcription which is perturbed in several pathological conditions, including cancer and inflammation.^[2]^ The BET family includes four members, BRD2, BRD3, BRD4 and BRDT, each containing two highly homologous N‐terminal bromodomain modules, namely BD1 and BD2, that are responsible for the recognition and binding to KAc (Figure 1).

Since the identification of the first potent and selective ligands, (+)‐**JQ1** (**1**) and **I**‐**BET** (**2**),^[3]^ several small molecule classes have been developed as BET inhibitors (iBETs)^[4]^ leading to further clinical development with applications in oncology.^[5]^ However, the majority of these inhibitors show no selectivity towards individual BET family members, thus hampering their scope as chemical probes for the clear definition of the physiopathological role of individual BET proteins. It is not surprising that side effects reported in clinical trials involving BET inhibitors are believed to stem from their lack of selectivity.^[6]^

Recent evidence suggests that BD1 is related to the maintenance of gene expression and, therefore, linked to antitumor effects. On the other hand, BD2 selective inhibition specifically affects the induction of gene expression whilst leaving the maintenance of established transcription programs largely unaltered. In clinical oncology, these results are of particular interest considering that both pan‐BET and BD1 selective inhibitors have similar efficacy, however BD1‐selective inhibition could achieve the desired clinical outcomes while limiting on‐target side effects.^[7]^

**Figure 2:**
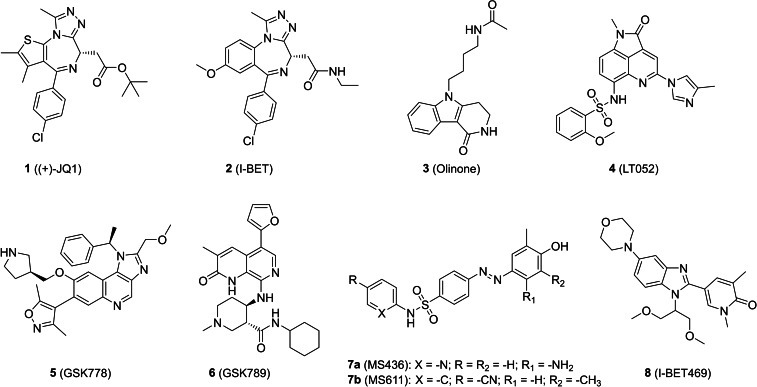
Structures of selected BET ligands.

It is therefore not surprising that the development of novel BD1‐selective BET inhibitors has gained a lot of attention. Development of site‐selective inhibitors is challenging because of the extremely high sequence identity shared between BET bromodomains, leading to only a small number of high selective compounds reported to date (Figure 2). Nevertheless, data from recent literature show considerable progress in the field. Starting from promising chemical scaffolds, several strategies have been reported for enhancing selectivity and potency of these early discovered compounds.^[8]^

One of the first examples of BD1 selective inhibitors was reported by Zhou and co‐workers. Starting from a tetrahydro‐pyrido indole scaffold, previously identified as inhibitor of the CBP BRD,^[9]^ a series of 1‐substituted‐ 2,3,4,5‐tetrahydro‐pyrido‐[4,3‐b]indol‐1‐ones was designed and tested as BET inhibitors. **Olinone** (**3**) showed a moderate affinity (K_D_=3.4 μM) but a preferential binding for BD1 over BD2 of all BET proteins.^[10]^ A fragment‐based screen identified the benzo[*cd*]indol‐2(1*H*)‐one core as promising scaffold for the development of BD1‐selective BET inhibitors. A subsequent series of structural modifications led to derivative **LT052** (**4**), which exhibited high potency towards BD1 (IC_50_=87.7 nM) and over 100‐fold selectivity for BD1 over BD2 when tested against BRD3 and BRD4.^[11]^ Recently, GlaxoSmithKline published two selective BD1 inhibitors: **GSK778** (**5**) and **GSK789** (**6**). Compound **5** showed a high inhibitory activity (IC_50_ from 41 nM to 75 nM) towards BD1 of all BET proteins with a good degree of selectivity (20–150 fold) for BD1 over BD2 of all the BET proteins. Despite the poor pharmacokinetic properties, **5** was used to demonstrate that selective BD1 inhibitors may be equally effective as pan‐BET inhibitors in cancer cells.^[7]^ Compound **6** showed a 1000‐fold selectivity for BD1 over BD2 in all BET proteins with an inhibition in the low nanomolar range (IC_50_=30 nM). Moreover, **6** showed the ability to inhibit cell growth of MV4‐11, HL60, and THP‐1 cell lines with IC_50_ values of 25, 390, and 258 nM, respectively. Despite the good biological activity, compound **5** suffers of high metabolic instability, probably due to the presence of an *N*‐methyl and electron‐rich aromatic portion, that prevented its further development.^[12]^

The azobenzene core has also been identified as a promising scaffold to develop selective iBETs. **MS436** (**7 a**) has nanomolar activity towards BD1 (BRD4 estimated K_i_=30–50 nM), with 10‐fold selectivity over BD2, while showing no selectivity between BD1 and BD2 of BRD2 and BRD3.^[13]^ **MS611** (**7 b**) exhibited better selectivity between BDs in the case of BRD4 (K_i_=0.41 μM and 41.3 μM for BD1 and BD2, respectively), but showed almost no difference in binding affinity between BD1 and BD2 of BRD2 and BRD3.[^10^, ^13^, ^14^] Although promising, these scaffolds have not been further developed, probably due to the presence of the azo‐moiety, which represent a metabolic hot spot^[15]^ besides the photoisomerization properties.^[16]^

Given our interest in the development of novel scaffolds for epigenetic modulators,^[17]^ we considered the azobenzene‐based compounds as a starting point to identify a novel chemotype as BD1 selective inhibitors with improved pharmacokinetic profiles. Here, we report the design, synthesis, and biochemical evaluation of novel benzimidazole‐based bromodomain BD1‐selective ligands. In addition, we evaluate the pharmacokinetic profile and in‐cell activity of the best performing compound.

## Results and Discussion

### Design, initial screening, and properties evaluation

From a structural point of view, we speculated that the azobenzene group in **7 a** and **7 b** (Figure 2) could be deemed as an open benzimidazole and, therefore, could be bioisosterically replaced by the latter. Benzimidazole, a privileged chemotype in medicinal chemistry, is metabolic stable and synthetically accessible.^[18]^ Moreover, it has been already proved to be an effective scaffold for the discovery of BRD ligands.^[19]^ In fact, GSK recently described a class of highly potent BET inhibitors featuring a dimethylphenol benzimidazole scaffold, identified by means of a DNA encoded library. The 3,5‐dimethylphenol portion contained in the compounds was identified as a metabolic hotspot and conveniently replaced with a 3,5‐dimethylpyridone. Further decoration of the benzimidazole scaffold finally yielded compound **8** (I‐BET469, Figure 2). This molecule showed a high activity *in vitro* (pIC_50_ of 7.9 on BD1 of BRD4), a 20 fold selectivity for BD1 over BD2 of BRD2, BRD3 and BRD4, and an efficient ability to engage BET proteins *in vivo*, inducing potent immunomodulatory effects.^[19b]^ However, despite these evidences, only few other reports describe benzimidazoles as BET ligands.^[20]^

**Figure 3:**
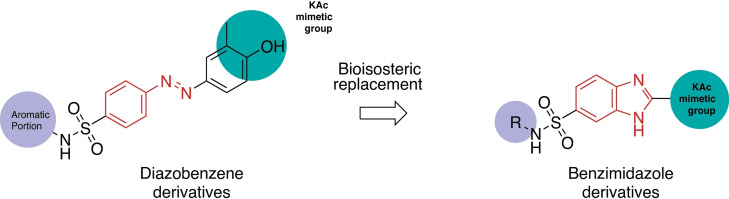
Design strategy.

Taking into account these considerations, we resolved to replace the azobenzene moiety of MS compounds (**7 a** and **7 b**) with a benzimidazole scaffold, with the goal to explore the effect of this bioisosteric substitution on potency, selectivity and pharmacokinetic properties (Figure 3).

**Figure 4:**
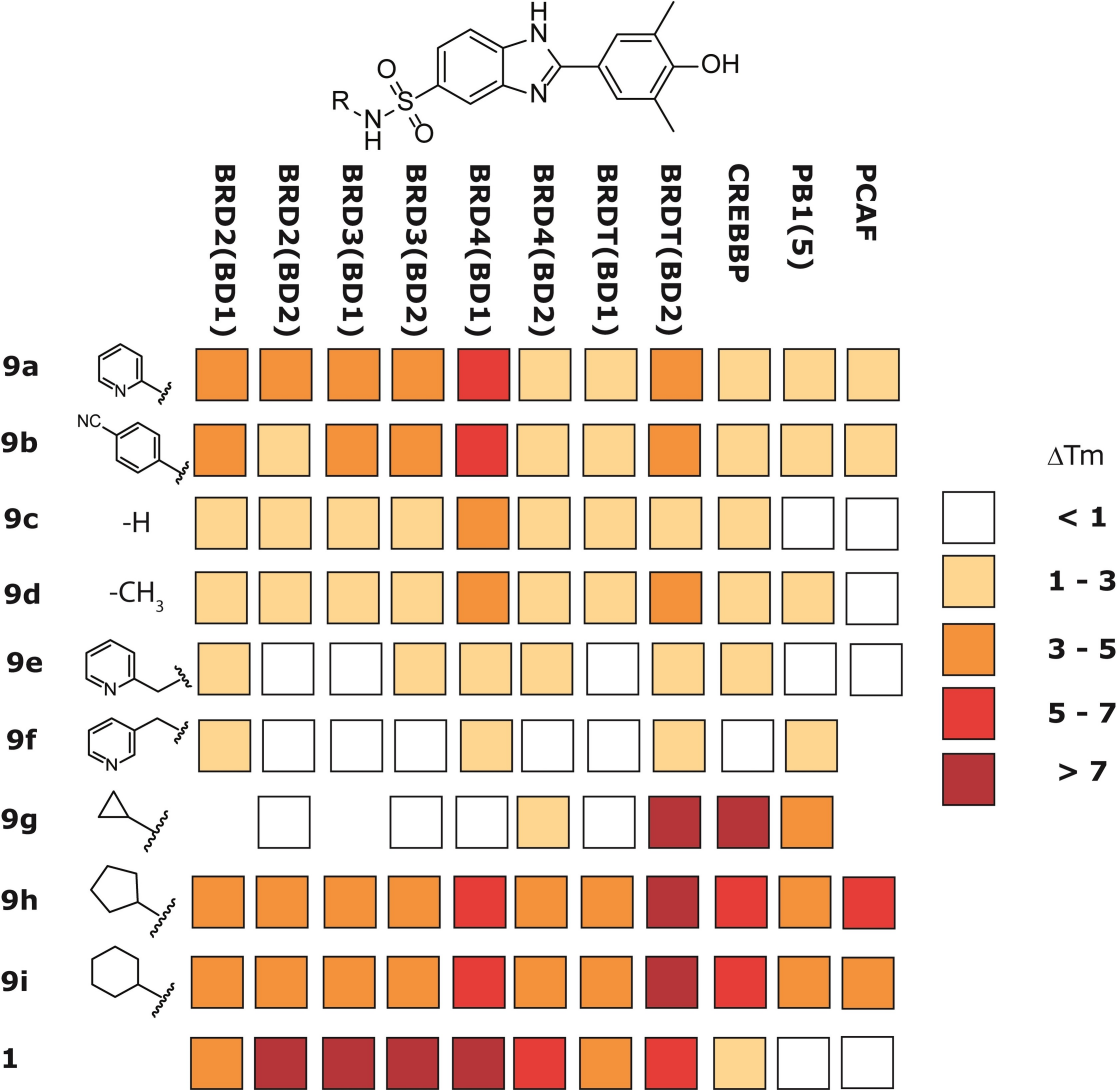
Results of ΔT_m_ screening of compounds **9 a**–**i** at 10 μM against BD1 and BD2 of BET proteins (BRD2, BRD3, BRD4 and BRDT) and three selected non‐BET bromodomain‐containing proteins (CREBBP, PB1 and PCAF).

**Scheme 1:**
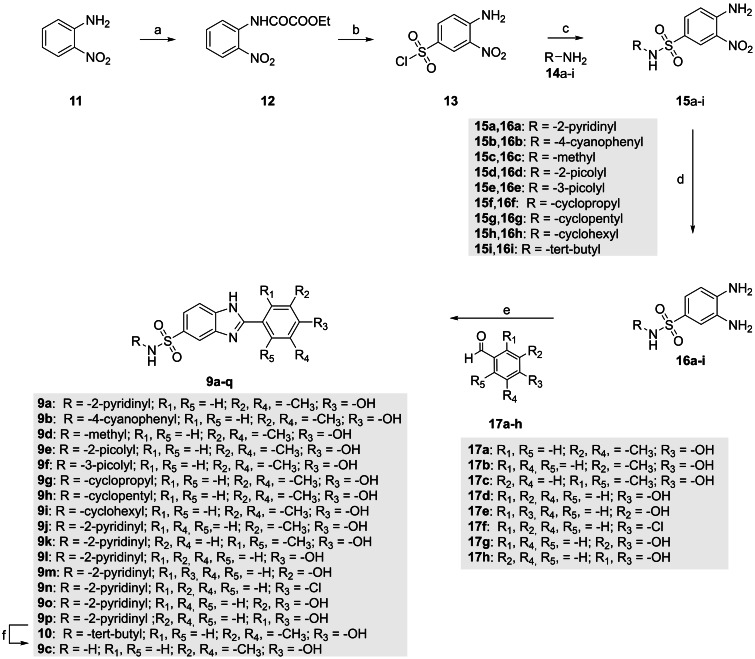
Synthesis of compounds **9 a**–**p**. *Reagents and conditions*: (a) Ethyl chlorooxoacetate, Et_2_O, r. t., 18 h (98 %); (b) ClSO_3_H, 80 °C, 3 h (99 %); (c) pyridine, 0 °C to r. t. for compounds **15 a**,**b** (35–40 %) or dry THF, r. t., 18 h for compounds **15 c**–**i**, (60–85 %); (d) Zn dust, AcOH, 4 h for compounds **16 a**,**b** (64–84 %) or H_2_ (1 atm, balloon), Pd/C (10 wt% on activated carbon), EtOH, 18 h for compounds **16 c**–**i** (89–97 %); (e) **17 a**–**h**, Na_2_S_2_O_5_, dry DMF, 80 °C, 18 h (54–85 %); (f) DCM/TFA (1 : 1), r. t. 18 h (74 %).

First, we synthesized and evaluated compounds **9 a** and **9 b** (Figure 4 and Scheme 1), which feature the benzimidazole moiety substituted with the 3,5‐dimethyl‐4‐phenolic group and a pyridine‐ (**9 a**) or a p‐cyanophenyl‐ sulfonamide (**9 b**), distinctive of compounds **7 a** and **7 b**, respectively.

The binding profile of these compounds was preliminary evaluated using a protein stability shift assay (ΔT_m_) against BET BD1/BD2 domains as well as against three representatives of diverse BRD families (CREBBP, PB1 and PCAF), in order to evaluate off‐target interactions (Figure 4). ΔT_m_ has emerged as a rapid and cost effective early screening method for the identification of BRD ligands and has been shown to correlate quite well with binding constants determined by other direct biophysical methods.[^3a^, ^21^] The compounds were tested at 10 μM fixed dose and the pan‐BET inhibitor **1** was used as a reference compound.^[22]^ The results obtained from this screening supported our design strategy. Indeed, compounds **9 a** and **9 b** are responsible for an increase in the melting temperature of all BET proteins while showing a certain degree of selectivity for the first bromodomain of BRD4.

These encouraging results led us to a preliminary investigation of the structure−activity relationship (SAR) on the sulfonamide nitrogen (compounds **9 c**–**i**, Figure 4). In accordance with the SAR of the azobenzene MS series,^[13]^ the removal of the aromatic moiety from the sulfonamide nitrogen was detrimental both for affinity and selectivity. In fact, the primary sulfonamide (**9 c**) and the methyl sulfonamide (**9 d**) showed a definitely lower affinity than **9 a** and **9 b** toward all the BET domains investigated. The detachment of the pyridine ring from the sulfonamide function, as in the picolyl derivatives **9 e** and **9 f**, resulted in complete loss of activity. On the other hand, the substitution of the pyridine moiety with an aliphatic ring shifted the activity also to non‐BET proteins. The insertion of a cyclopentyl or a cyclohexyl group (**9 h** and **9 i**, respectively) yielded compounds with an affinity toward BETs similar to **9 a** and **9 b** but with a strong binding to BD2 of BRDT and other non‐BET bromodomain‐containing proteins. It is worth to note that the substitution of an aromatic moiety with a cyclopropyl group (**9 g**) resulted in strong off‐target binding together with a partial loss of affinity toward BETs, while retaining binding only towards the BD2 of BRDT.

### Physiochemical properties of 2‐aryl‐benzimidazole‐6‐sulfonamides ligands

Proved our design strategy, in order to select the best derivative for further development, we evaluated key properties of benzimidazole compounds **9 a** and **9 b** that could affect pharmacokinetics. Results are reported in Table 1. Azobenzene derivatives **7 a** and **7 b** were also evaluated for comparison.

**Table 1:** Physiochemical Properties **of 7 a**, **7 b**, **9 a**, **9 b**.

#	CYPs and UGTs “dual‐activity” microsomal stability^[a]^	Solubility^[b]^	Pampa P_app_ [cm/s]	cLogP^[c]^
**7 a**	7.8±0.5	100 μM	1.03×10^−8^	2.50
**7 b**	7.5±1.0	50 μM	1.10×10^−6^	3.93
**9 a**	1.0±0.3	100 μM	1.07×10^−7^	2.82
**9 b**	16.3±0.5	50 μM	9.09×10^−8^	3.35

[a] Microsomal stability of compounds in the presence of alamethicin, NADPH and UDPGA cofactors. Value expressed as the percentage of the parent compound turnover. [b] Highest concentration of the compound where no precipitate was detected. [c] Calculated with SWISSADME (http://www.swissadme.ch/).

Determination of metabolic properties of bioactive molecules is one of the most important steps during the drug development process. The CYPs play an important role in drug oxidative metabolism and they are capable of converting molecules to more polar metabolites using NADPH as the cofactor.^[23]^ The UGT superfamily of enzymes catalyzes the conjugation of *d*‐glucuronic acid by transferring a glucuronic acid moiety from the cofactor uridine 5′‐diphosphoglucuronic acid (UDPGA) to substrates containing an accepting group.^[23]^ It is more common that glucuronidation occurs after xenobiotics are metabolized by phase I enzymes such as CYPs. This metabolic reaction forms water‐soluble compounds that readily excreted via urine or bile.

In this study, the CYPs and UGTs “dual‐activity” microsomal stability assay was carried out to follow the loss of the test compounds over time under CYP‐ and UGT‐mediated metabolic pathways. In detail, the compounds were added to human liver microsomes in the presence of alamethicin, NADPH and UDPGA as cofactors.^[24]^ Alamethicin, a pore‐forming peptide, was used to activate UGTs in human liver microsomes. Testosterone was used as positive control while the negative control was prepared by incubation of the compounds without cofactors up to 60 min. The negative control is essential to detect problems such as non‐specific protein binding or heat instability. Notably, all compounds showed no binding to proteins and high stability in absence of cofactors.

Results summarized in Table 1 indicate that compound **9 a** was very stable, showing a percentage of the parent compound turnover of 1.0±0.3 %, significantly lower than other compounds tested. Unpredictably,^[25]^ the *p*‐cyanophenyl substituent (**9 b**) led to poor metabolic stability, even lower than the related azobenzene analogue **7 b**.

Next, using nephelometric measurements, we determined the solubility profile of these compounds in aqueous solutions with 1 % DMSO. All compounds displayed good solubility profiles at 50 μM; however, when tested at 100 μM, only compounds **9 a** and **7 a** were still soluble.

Membrane permeability was estimated by the well‐validated parallel artificial membrane permeability assay (PAMPA) technique.[^17^, ^26^] To establish and validate our in‐house assay, the highly permeable propranolol (*P_app_* =1.91×10^−6^ cm/s) and the poor permeable furosemide (*P_app_* =1.01×10^−8^ cm/s) were used as positive and negative controls, respectively. Compounds **7 a** and **7 b** were evaluated for comparison. Data reported in Table 1 indicated that the two azo‐benzene compounds showed respectively the lowest (1.03×10^−8^ cm/s) and the highest (1.10×10^−6^ cm/s) P_*app*_ value among the selected compounds. On the other hand, benzimidazole compounds **9 a** and **9 b** were comparable in terms of apparent permeability, both showing values (1.07×10^−7^ and 9.09×10^−8^ cm/s, respectively) that were higher than that of compound **7 a**. As compound **7 a** showed good cellular efficacy, it can be speculated that also our benzimidazole derivatives could have an acceptable cellular permeability.

Finally, the calculation of cLogP proved that **9 a** showed also a good cLogP value of 2.82, comparable to the one of **7 a** and lower than **9 b**. This is a highly desirable feature as a compound with a cLogP lower than 3 has less chance to give undesired side effects *in vivo*.^[27]^

Taken together, these results supported our hypothesis of bioisosterically replacing azobenzene with benzimidazole and indicated **9 a** as the most attractive compound.

### Further structure−activity relationship studies

**Figure 5:**
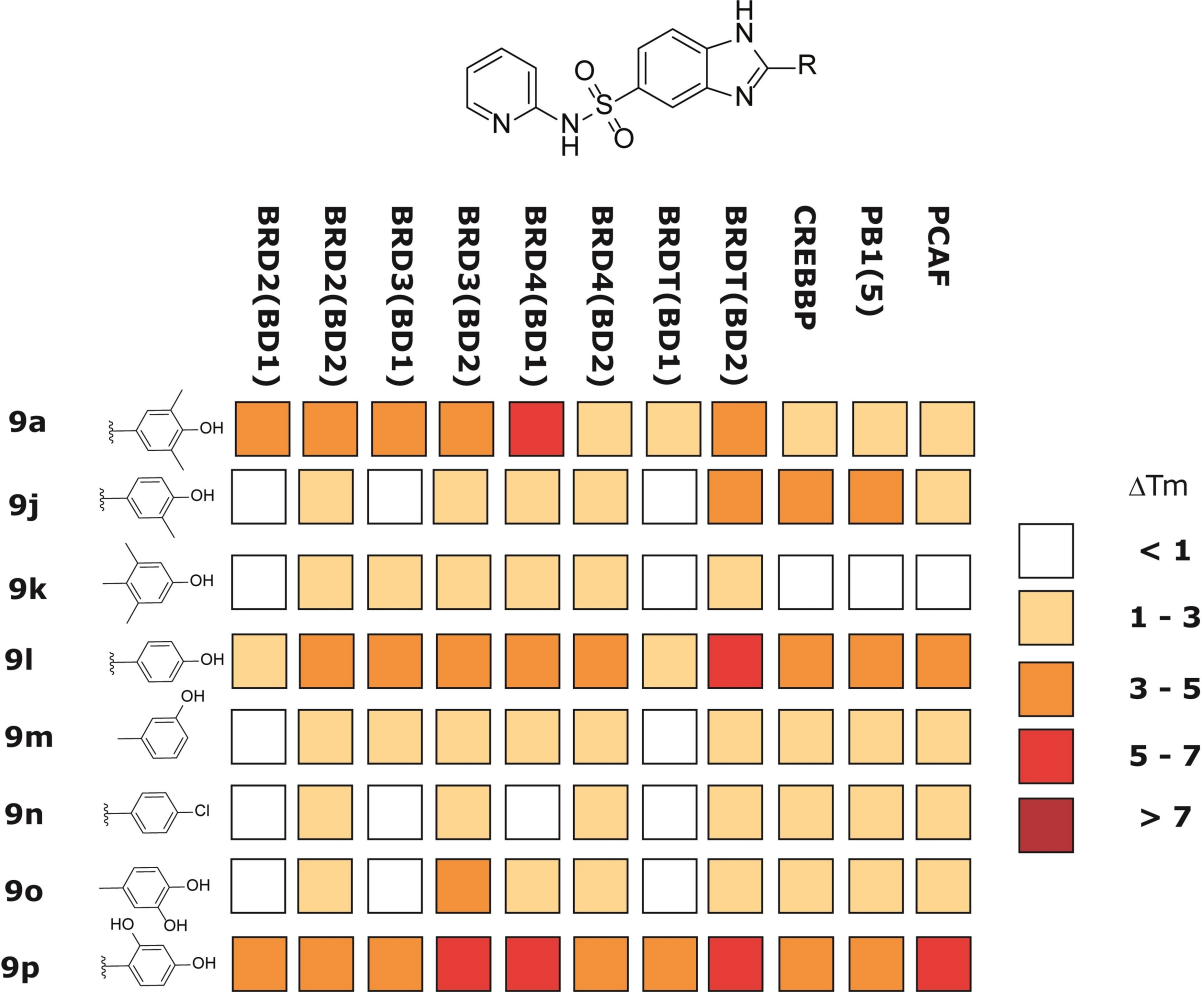
ΔT_m_ results for compounds **9 a**, **9 j**–**p** at 10 μM against BD1 and BD2 of BET proteins (BRD2, BRD3, BRD4 and BRDT) and three selected non‐BET bromodomain‐containing proteins (CREBBP, PB1 and PCAF).

In a second round of structure‐activity studies, we kept the pyridine sulfonamide moiety of **9 a** and explored the effect of structural modifications on the phenyl ring in position 2 of the benzimidazole (compounds **9 j**–**p**, Figure 5 and Scheme 1). At this stage, we explored the effect of substitution patterns on phenol moiety to get insights on the structure requirement for this warhead.

ΔT_m_ confirmed that the 3,5‐dimethyl‐4‐phenolic substitution is responsible of the activity profile of compound **9 a** and that all the other modifications negatively affected both activity and/or selectivity. This is consistent with previous observations that the 2,6‐dimethylphenol moiety mimics the KAc group.[^19b^, ^28^] Indeed, removal of the 5‐methyl (**9 j**) as well as the formal shift of methyl groups to the 2,6 positions (**9 k**) resulted in a marked loss of activity. The unsubstituted phenol derivative (**9 l**) is still active but significantly less selective. In addition, the presence and the correct position of the phenolic substitution are both important for the activity. For example, shifting to the 3‐position (**9 m**) or its substitution with a chlorine (**9 n**) resulted in almost completely inactive products. Moreover, the catechol substitution is not tolerated (**9 o**) while the 2,4‐diphenolic substitution (**9 p**) furnished an active but non‐selective compound.

### Binding validation of 9 a to BD1 and selectivity

**Figure 6:**
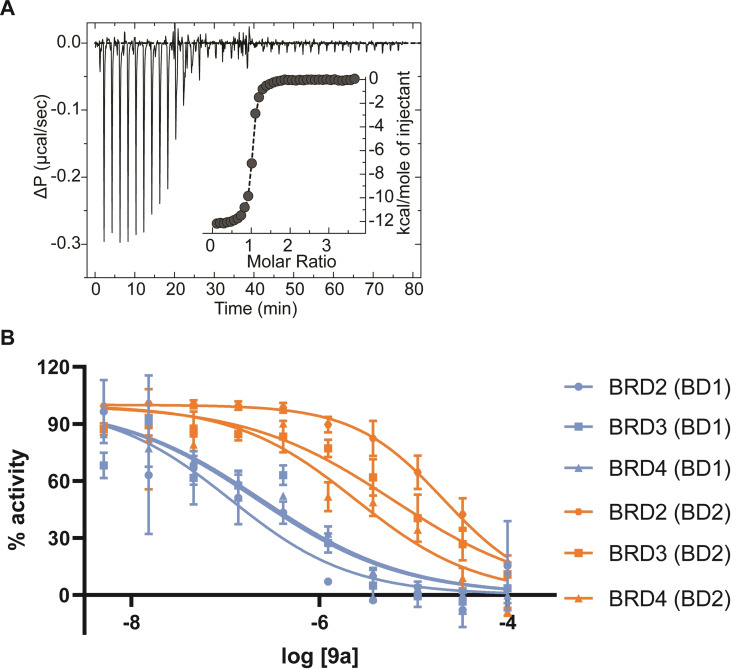
(a) ITC binding curve of **9 a** to BRD4 BD1; (K_D_=70.4±3.7 nM) (b) TR‐FRET curves of **9 a** with BD1 and BD2 domains of BET proteins (BRD2, BRD3, BRD4). The compound was tested in 11‐concentration IC_50_ mode with 3‐fold serial dilutions starting from a concentration of 100 μM. Data were analysed using GraphPad Prism software (version 8.0) for curve fitting, using a sigmoidal concentration−response with a variable slope equation.

To quantify the affinity for BRD4 BD1 protein, isothermal titration calorimetry (ITC) was used. This biophysical method confirmed the binding of **9 a** (Figure 6), showing a K_D_ value of 70 nM, which is comparable to the one of the azobenzene series.^[13]^

To define the selectivity profile of **9 a** we employed a time‐resolved fluorescence resonance energy transfer (TR‐FRET) displacement binding assay using an AlexaFluor647 dye conjugated to **1**, as previously described.^[29]^ The curves (Figure 6) and the IC_50_ values (Table 2) showed that compound **9 a** is at least 150‐fold selective for the BD1 (IC_50_=0.126 μM) domain of BRD2 over BD2 (IC_50_=22 μM). This good BD1/BD2 selectivity profile was confirmed also for BRD3 and BRD4, even if at a lesser extent (40‐ and 10‐fold respectively).

**Table 2:** Selectivity profile of **9 a**.

Target protein	**9 a** [IC_50_ μM]	BD1 selectivity index
BRD2(BD1)	0.126±0.019	170
BRD2(BD2)	22±8	
BRD3(BD1)	0.18±0.02	40
BRD3(BD2)	7.0±3.0	
BRD4(BD1)	0.252±0.041	10
BRD4(BD2)	2.2±1.0	

If compared with the results reported for **7 a** and **7 b**, compound **9 a** displayed a better selectivity profile towards all BD1 of BETs. In fact **7 a** and **7 b**, despite the good BD1 activity profile, showed almost no selectivity between each BDs domain of BRD3 and BRD2.[^10^, ^13^, ^14^]

### Effect on cell viability and protein expression regulated by BRD

**Figure 7:**
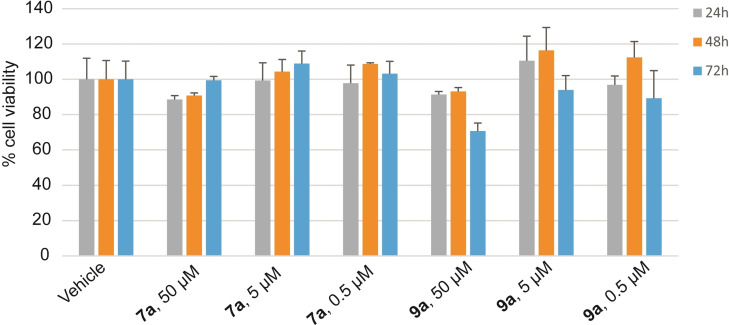
Cell toxicity of compounds **9 a** and **7 a** in HeLa cell line. Cells were treated for 24, 48, and 72 h with compounds **7 a** and **9 a** at the concentrations of 50, 5, and 0.5 μM. Cell viability was assessed by measuring the mitochondrial‐dependent reduction of MTT to formazan. Data are reported as mean±SD of at least three independent experiments.

The good preliminary data obtained for compound **9 a** combining different techniques prompted us to investigate the effect of this compound in human cells. First, to determine the proper concentration to be used for the evaluation of BET inhibitory activity in cellular contest, the cellular toxicity of compound **9 a** was assessed in HeLa cells, using compound **7 a** as a reference (Figure 7).

Cells were incubated with different compound concentrations (50 μM, 5 μM and 0.5 μM) for different times (24, 48 and 72 h) and cellular viability was determined performing an MTT (3‐(4,5‐dimethylthiazol‐2‐yl)‐2,5‐diphenyl‐2*H*‐tetrazolium bromide) assay. Results obtained clearly showed a survival of the cells at all the tested concentrations and times and only a slight decrease of cell viability (around 30 %) was detected after treatment with **9 a** at the highest concentration (50 μM) for 72 h.

Thereafter we evaluated the ability of compound **9 a** to inhibit BET protein activity in cells measuring the protein levels of c‐Myc, a well‐established BET transcriptional target.^[30]^

**Figure 8:**
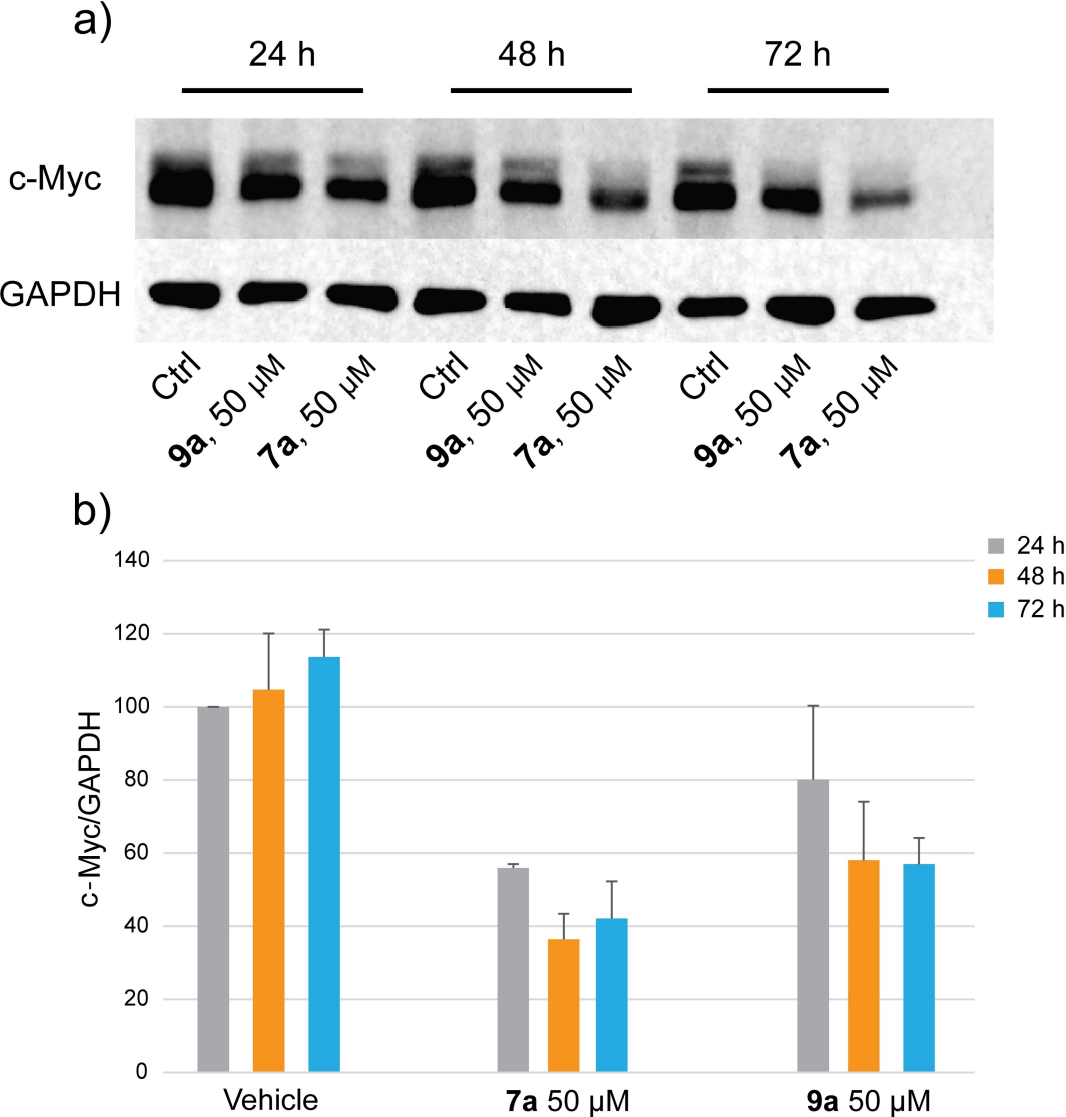
Western blot images (a) and densitometric analysis (b) of lysates derived from HeLa cells treated with compound **9 a** at 50 μM for 24, 48 and 72 h on the levels of c‐Myc. GADPH was used to check for equal loading. Compound **7 a** (50 μM) was used as a reference compound. Signals were detected using the ImageQuant LAS 4000 (GE Healthcare, Waukesha, WI) digital imaging system and quantified by ImageQuantTL software.

HeLa cells were incubated with 50 μM of **9 a** and **7 a** for 24, 48 and 72 h. Cells were lysed and immunoblotted with a c‐Myc specific antibody. GAPDH was used for normalization and for checking equal loading. As shown in Figure 8, even if lower than related compound **7 a**, derivative **9 a** induced a reduction (from 20 % to 40 %) of the c‐Myc expression levels at all the tested times.

### Chemistry

The compounds **9 a**–**p** were prepared as depicted in Scheme 1. Key intermediates for the preparation of target compounds **9 a**–**p** are the 3,4‐diaminobenzenesulfonamides **16 a**–**i** which were synthetized as previously reported by us.^[31]^ Briefly, treatment with ethyl chlorooxoacetate of the 2‐nitroaniline **11** in diethyl ether (Et_2_O) furnished the protected amino compound **12**. The reaction of the latter with the chlorosulfonic acid at 80 °C, yielded the unprotected sulfonyl chloride **13** after an aqueous workup. The subsequent reaction with the appropriate amines (**14 a**–**i**), gave the corresponding *N*‐substituted‐4‐amino‐3‐nitrobenzenesulfonamides (**15 a**–**i**). Noteworthy, the preparation of *N*‐aryl sufonamides **15 a**,**b** in approximately 40 % yield required the use of pyridine as a solvent at 0 °C. On the other hand, preparation of *N*‐alkyl sulfonamides **15 c**–**i** proceeded in good yields (60–85 %) using dry THF as solvent at room temperature. From nitro derivatives **15 a**–**i**, zinc dust reduction in acetic acid (AcOH) or palladium‐catalysed hydrogenation furnished the corresponding 3,4‐diaminobenzenesulfonamides **16 a**,**b** and **16 c**–**i**, respectively. Finally, cyclocondensation‐dehydrogenation of the di‐amino derivatives with commercially available aldehydes **17 a**–**h** in dry DMF at 80 °C afforded the benzimidazole‐based compounds **9 a**, **9 b**, **9 d**–**9 p** and **10**. Deprotection under acidic conditions (DCM/TFA 1 : 1) of the intermediate **10** furnished the primary sulfonamide **9 c**.

## Conclusion

In this manuscript, we reported the design, synthesis, and biological evaluation of a series of benzimidazole‐6‐sulfonamides as BET ligands. Starting from **7 a** and **7 b**, ligands that exhibited a preference for BD1 of BET family members, the novel active compounds have been developed by the bioisosteric replacement of the unfavorable azobenzene moiety, that could give rise to toxic metabolites in particular in hypoxic tumor microenvironments, with a benzimidazole ring.

The most promising compound (**9 a**) showed good potency in binding BET proteins and a good degree of selectivity towards BD1 of all BETs with respect to the parent compounds. **9 a** is soluble, cell‐permeable and features *in vitro* metabolic stability. Moreover, it is able to reduce the level of c‐Myc transcription in HeLa cells, without showing significant cytotoxicity. Taken together, these results confirmed the benzimidazole as useful bioisostere of azobenzene moiety and endorse benzimidazole‐6‐sulfonamide as a viable chemical template to obtain compounds with improved selectivity towards the first bromodomains of BET family proteins.

## Experimental Section

**Chemistry**. *General directions*. All chemicals, purchased from Merck KGaA and Fluorochem Ltd., were of the highest purity. All solvents were reagent grade and, when necessary, were purified and dried by standard methods. All reactions requiring anhydrous conditions were conducted under a positive atmosphere of nitrogen in oven‐dried glassware. Standard syringe techniques were used for anhydrous addition of liquids. Reactions were routinely monitored by TLC performed on aluminum‐backed silica gel plates (Merck KGaA, Alufolien Kieselgel 60 F254) with spots visualized by UV light (λ=254, 365 nm) or using a KMnO_4_ alkaline solution. Solvents were removed using a rotary evaporator operating at a reduced pressure of ∼10 Torr. Organic solutions were dried over anhydrous Na_2_SO_4_. Analytical high performance liquid chromatography (HPLC) was performed on a Shimadzu SPD 20A UV/VIS detector (λ=220 and 254 nm) using C‐18 column Phenomenex Synergi Fusion – RP 80 A (75×4.60 mm; 4 μm) at 25 °C using a mobile phase A (water+0.1 % TFA) and B (ACN+0.1 % TFA) at a flow rate of 1 mL/min. ^1^H spectra were recorded at 400 MHz on a Bruker Ascend 400 spectrometer while ^13^C NMR spectra were obtained by distortionless enhancement by polarization transfer quaternary (DEPTQ) spectroscopy on the same spectrometer. Chemical shifts are reported in δ (ppm) relative to the internal reference tetramethylsilane (TMS). Due to the existence of tautomers, some ^1^H and ^13^C NMR signals could not be detected for some of the prepared benzimidazoles so only the distinct signals are reported. Low resolution mass spectra were recorded on a Finnigan LCQ DECA TermoQuest mass spectrometer in electrospray positive and negative ionization modes (ESI‐MS). High resolution mass spectra were recorded on a ThermoFisher Scientific Orbitrap XL mass spectrometer in electrospray positive ionization modes (ESI‐MS). All tested compounds possessed a purity of at least 95 % established by HPLC unless otherwise noted. Compounds **12** and **13** were prepared as previously described by us.^[31]^

**2‐(4‐hydroxy‐3,5‐dimethylphenyl)‐*N*‐(pyridin‐2‐yl)‐1*H*‐benzo[*d*]imidazole‐6‐sulfonamide (9 a)**. To a solution of 3,4‐diamino‐*N*‐(pyridin‐2‐yl)benzenesulfonamide **16 a** (200 mg, 0.76 mmol) in dry DMF (6.0 mL), 4‐hydroxy‐3,5‐dimethylbenzaldehyde **17 a** (114 mg, 0.76 mmol) and Na_2_S_2_O_5_ (188 mg, 0.99 mmol) were added, and the resulting mixture was heated at 80 °C for 18 h. After cooling at room temperature, water was added. The brown precipitate formed was filtered and washed with water. Compound **9 a** (255 mg, 85 %) was obtained as light‐yellow solid after recrystallization from EtOH. ^1^H NMR (400 MHz, DMSO‐*d_6_*) *δ* 8.97 (s, 1H, exchangeable with D_2_O), 8.07–8.00 (m, 2H), 7.78 (s, 2H), 7.73–7.68 (m, 2H), 7.68–7.63 (m, 1H), 7.18 (d, *J*=8.6 Hz, 1H), 6.90–6.83 (m, 1H), 2.26 (s, 6H).^13^C NMR (101 MHz, DMSO‐*d*_6_) δ 156.11, 154.44, 139.92, 127.30, 124.82, 120.69, 119.28, 113.39, 16.69. HRMS (ESI): *m/z* [M+H]^+^ calcd. for C_20_H_18_N_4_O_3_S+H^+^: 395.1172. Found: 395.1157.

***N*** **‐(4‐cyanophenyl)‐2‐(4‐hydroxy‐3,5‐dimethylphenyl)‐1*H*‐benzo[*d*]imidazole‐6‐sulfonamide (9 b)**. Compound **9 b** (86 mg, 66 %) was obtained as a white solid from derivative **16 b** (89 mg, 0.31 mmol) and 4‐hydroxy‐3,5‐dimethylbenzaldehyde **17 a** (47 mg, 0.31 mmol) according to the procedure described for **9 a**. ^1^H NMR (400 MHz, DMSO‐*d_6_*) *δ* 10.99 (s, 1H, exchangeable with D_2_O), 8.99 (s, 1H, exchangeable with D_2_O), 8.00–7.97 (m, 1H), 7.77 (s, 2H), 7.72–7.62 (m, 4H), 7.27 (d, *J*=8.8 Hz, 2H), 2.26 (s, 6H).^13^C NMR (101 MHz, DMSO‐*d*_6_) *δ* 156.40, 154.66, 142.48, 133.65, 132.48, 127.45, 124.89, 120.70, 118.70, 118.41, 105.21, 16.68. HRMS (ESI): *m/z* [M+H]^+^ calcd. for C_22_H_18_N_4_O_3_S+H^+^: 419.1172. Found: 419.1161.

**2‐(4‐hydroxy‐3,5‐dimethylphenyl)‐1*H*‐benzo[*d*]imidazole‐6‐sulfonamide (9 c)**. *N*‐(*tert*‐butyl)‐2‐(4‐hydroxy‐3,5‐dimethylphenyl)‐1H‐benzo[*d*]imidazole‐6‐sulfonamide **10** (134 mg, 0.36 mmol) was dissolved in 4 mL of DCM/TFA (1 : 1) and the mixture was stirred at room temperature. After 18 h, the solvent was evaporated, and the title compound (85 mg, 74 %) was obtained as a light‐brown solid after crystallization from EtOH. ^1^H NMR (400 MHz, DMSO‐*d*_6_) *δ* 12.98 (s, 1H, exchangeable with D_2_O), 8.87 (s, 1H, exchangeable with D_2_O), 8.06–7.87 (m, 1H), 7.79 (s, 2H), 7.74–7.56 (m, 2H), 7.30–7.21 (m, 2H, exchangeable with D_2_O), 2.26 (s, 6H). ^13^C NMR (101 MHz, DMSO‐*d*_6_) *δ* 155.72, 155.62, 154.88, 154.37, 146.02, 143.16, 137.55, 137.45, 137.06, 134.14, 127.10, 124.72, 120.29, 119.57, 119.21, 118.16, 116.11, 111.02, 109.10, 16.70. HRMS (ESI): *m/z* [M+H]^+^ calcd. for C_15_H_15_N_3_O_3_S+H^+^: 318.0907. Found: 318.0899.

**2‐(4‐hydroxy‐3,5‐dimethylphenyl)‐*N*‐methyl‐1*H*‐benzo[*d*]imidazole‐6‐sulfonamide (9 d)**. Compound **9 d** (60 mg, 73 %) was obtained as a white solid from derivative **16 c** (50 mg, 0.25 mmol) and 4‐hydroxy‐3,5‐dimethylbenzaldehyde **17 a** (38 mg, 0.25 mmol) according to the procedure described for **9 a**. ^1^H NMR (400 MHz, DMSO‐*d*_6_) *δ* 13.02 (s, 1H, exchangeable with D_2_O), 8.98 (s, 1H, exchangeable with D_2_O), 7.97–7.86 (m, 1H), 7.79 (s, 2H), 7.68 (d, *J*=8.5 Hz, 1H), 7.57 (dd, *J*=8.5, 1.7 Hz, 1H), 7.36–7.28 (m, 1H, exchangeable with D_2_O), 2.40 (d, *J*=4.8 Hz, 3H), 2.27 (s, 6H). ^13^C NMR (101 MHz, DMSO‐*d*_6_) *δ* 155.75, 132.12, 127.15, 124.72, 120.20, 28.74, 16.70. HRMS (ESI): *m/z* [M+H]^+^ calcd. for C_16_H_17_N_3_O_3_S+H^+^: 332.1063. Found: 332.1052.

**2‐(4‐hydroxy‐3,5‐dimethylphenyl)‐*N*‐(pyridin‐2‐ylmethyl)‐1*H*‐benzo[*d*]imidazole‐6‐sulfonamide (9 e)**. Compound **9 e** (245 mg, 56 %) was obtained as a light‐yellow solid from derivative **16 d** (299 mg, 1.07 mmol) and 4‐hydroxy‐3,5‐dimethylbenzaldehyde **17 a** (161 mg, 1.07 mmol) according to the procedure described for **9 a**. ^1^H NMR (400 MHz, DMSO‐*d*_6_) *δ* 13.01 (s, 1H, exchangeable with D_2_O), 8.88 (s, 1H, exchangeable with D_2_O), 8.43–8.39 (m, 1H), 8.15–8.08 (m, 1H, exchangeable with D_2_O), 8.04–7.85 (m, 1H), 7.80 (s, 2H), 7.74–7.67 (m, 1H), 7.65–7.55 (m, 2H), 7.37 (d, *J*=7.9 Hz, 1H), 7.25–7.18 (m, 1H), 4.05 (d, *J*=5.8 Hz, 2H), 2.27 (s, 6H).^13^C NMR (101 MHz, DMSO‐*d*_6_) *δ* 157.30, 155.72, 148.65, 136.60, 133.32, 127.13, 124.70, 122.27, 121.57, 120.19, 48.03, 16.69. HRMS (ESI): *m/z* [M+H]^+^ calcd. for C_21_H_20_N_4_O_3_S+H^+^: 409.1329. Found: 409.1313.

**2‐(4‐hydroxy‐3,5‐dimethylphenyl)‐*N*‐(pyridin‐3‐ylmethyl)‐1*H*‐benzo[*d*]imidazole‐6‐sulfonamide (9 f)**. Compound **9 f** (68 mg, 72 %) was obtained as a light‐yellow solid from derivative **16 e** (64 mg, 0.23 mmol) and 4‐hydroxy‐3,5‐dimethylbenzaldehyde **17 a** (34 mg, 0.23 mmol) according to the procedure described for **9 a**. ^1^H NMR (400 MHz, DMSO‐*d*_6_) *δ* 13.02 (s, 1H, exchangeable with D_2_O), 8.89 (s, 1H, exchangeable with D_2_O), 8.43–8.41 (m, 1H), 8.41–8.38 (m, 1H), 8.12 (t, *J*=6.3 Hz, 1H, exchangeable with D_2_O), 7.94 (s, 1H), 7.80 (s, 2H), 7.70–7.57 (m, 3H), 7.28 (dd, *J*=7.9, 4.8 Hz, 1H), 4.02 (d, *J*=6.3 Hz, 2H), 2.27 (s, 6H). ^13^C NMR (101 MHz, DMSO‐*d*_6_) *δ* 155.74, 148.83, 148.25, 135.33, 133.39, 127.14, 124.71, 123.24, 120.17, 120.11, 43.76, 16.69. HRMS (ESI): *m/z* [M+H]^+^ calcd. for C_21_H_20_N_4_O_3_S+H^+^: 409.1329. Found: 409.1316.

***N*** **‐cyclopropyl‐2‐(4‐hydroxy‐3,5‐dimethylphenyl)‐1*H*‐benzo[*d*]imidazole‐6‐sulfonamide (9 g)**. Compound **9 g** (63 mg, 63 %) was obtained as a light‐yellow solid from derivative **16 f** (64 mg, 0.28 mmol) and 4‐hydroxy‐3,5‐dimethylbenzaldehyde **17 a** (42 mg, 0.28 mmol) according to the procedure described for **9 a**. ^1^H NMR (400 MHz, DMSO‐*d_6_*) *δ* 13.02 (s, 1H, exchangeable with D_2_O), 8.88 (s, 1H, exchangeable with D_2_O), 8.01–7.87 (m, 1H), 7.85–7.75 (m, 3H, 1H exchangeable with D_2_O), 7.74–7.65 (m, 1H), 7.61 (dd, *J*=8.4, 1.8 Hz, 1H), 2.27 (s, 6H), 2.11–2.03 (m, 1H), 0.49–0.41 (m, 2H), 0.42–0.36 (m, 2H). ^13^C NMR (101 MHz, DMSO‐*d_6_*) *δ* 155.73, 133.21, 127.14, 124.72, 120.20, 24.15, 16.70, 5.07. HRMS (ESI): *m/z* [M+H]^+^ calcd. for C_18_H_19_N_3_O_3_S+H^+^: 358.1220. Found: 358.1208.

***N*** **‐cyclopentyl‐2‐(4‐hydroxy‐3,5‐dimethylphenyl)‐1*H*‐benzo[*d*]imidazole‐6‐sulfonamide (9 h)**. Compound **9 h** (342 mg, 58 %) was obtained as a yellow solid from derivative **16 g** (390 mg, 1.53 mmol) and 4‐hydroxy‐3,5‐dimethylbenzaldehyde **17 a** (230 mg, 1.53 mmol) according to the procedure described for **9 a**. ^1^H NMR (400 MHz, DMSO‐*d*_6_) *δ* 9.19 (s, 1H, exchangeable with D_2_O), 8.03–7.97 (m, 1H), 7.82 (s, 2H), 7.77 (d, *J*=8.5 Hz, 1H), 7.72 (dd, *J*=8.5, 1.7 Hz, 1H), 7.68–7.64 (m, 1H exchangeable with D_2_O), 3.45–3.35 (m, 1H), 2.28 (s, 6H), 1.60–1.49 (m, 4H), 1.39–1.26 (m, 4H). ^13^C NMR (101 MHz, DMSO‐*d*_6_) *δ* 156.90, 153.72, 136.05, 127.69, 125.06, 121.44, 117.51, 114.46, 113.29, 54.47, 32.42, 22.77, 16.68. HRMS (ESI): *m/z* [M+H]^+^ calcd. for C_20_H_23_N_3_O_3_S+H^+^: 386.1533. Found: 386.1524.

***N*** **‐cyclohexyl‐2‐(4‐hydroxy‐3,5‐dimethylphenyl)‐1*H*‐benzo[*d*]imidazole‐6‐sulfonamide (9 i)**. Compound **9 i** (160 mg, 54 %) was obtained as a yellow solid from derivative **16 h** (200 mg, 0.74 mmol) and 4‐hydroxy‐3,5‐dimethylbenzaldehyde **17 a** (111 mg, 0.74 mmol) according to the procedure described for **9 a**. ^1^H NMR (400 MHz, DMSO‐*d*_6_) *δ* 13.00 (s, 1H, exchangeable with D_2_O), 8.88 (s, 1H, exchangeable with D_2_O), 8.03–7.83 (m, 1H), 7.79 (s, 2H), 7.73–7.58 (m, 2H, 1H exchangeable with D_2_O), 7.55–7.46 (m, 1H), 2.97–2.82 (m, 1H), 2.27 (s, 6H), 1.57–1.39 (m, 5H), 1.19–0.95 (m, 5H). ^13^C NMR (101 MHz, DMSO‐*d*_6_) *δ* 155.71, 135.27, 127.12, 124.71, 120.23, 52.01, 33.18, 24.87, 24.33, 16.70. HRMS (ESI): *m/z* [M+H]^+^ calcd. for C_21_H_25_N_3_O_3_S+H^+^: 400.1689. Found: 400.1675.

**2‐(4‐hydroxy‐3‐methylphenyl)‐*N*‐(pyridin‐2‐yl)‐1*H*‐benzo[*d*]imidazole‐6‐sulfonamide (9 j)**. Compound **9 j** (130 mg, 54 %) was obtained as a light‐yellow solid from derivative **16 a** (167 mg, 0.63 mmol) and 4‐hydroxy‐3‐methylbenzaldehyde **17 b** (86 mg, 0.63 mmol) according to the procedure described for **9 a**. ^1^H NMR (400 MHz, DMSO‐*d*_6_) *δ* 10.39 (s, 1H, exchangeable with D_2_O), 8.13–8.08 (m, 1H), 8.03–7.98 (m, 1H), 7.94 (d, *J*=2.3 Hz, 1H), 7.87 (dd, *J*=8.4, 2.3 Hz, 1H), 7.82 (dd, *J*=8.5, 1.7 Hz, 1H), 7.79–7.68 (m, 2H), 7.21 (d, *J*=8.5 Hz, 1H), 7.00 (d, *J*=8.5 Hz, 1H), 6.91–6.83 (m, 1H), 2.23 (s, 3H). ^13^C NMR (101 MHz, DMSO‐*d*_6_) *δ* 159.42, 153.40, 153.11, 140.46, 130.07, 126.88, 125.22, 121.91, 115.82, 115.67, 114.30, 114.27, 113.23, 15.97. HRMS (ESI): *m/z* [M+H]^+^ calcd. for C_19_H_16_N_4_O_3_S+H^+^: 381.1016. Found: 381.1000.

**2‐(4‐hydroxy‐2,6‐dimethylphenyl)‐*N*‐(pyridin‐2‐yl)‐1*H*‐benzo[*d*]imidazole‐6‐sulfonamide (9 k)**. Compound **9 k** (190 mg, 67 %) was obtained as a pale‐yellow solid from derivative **16 a** (190 mg, 0.72 mmol) and 4‐hydroxy‐2,6‐dimethylbenzaldehyde **17 c** (108 mg, 0.72 mmol) according to the procedure described for **9 a**. ^1^H NMR (400 MHz, DMSO‐*d*_6_) *δ* 12.83 (s, 1H, exchangeable with D_2_O), 9.60 (s, 1H, exchangeable with D_2_O), 8.19–8.15 (m, 1H), 8.08–8.00 (m, 1H), 7.77–7.65 (m, 3H, 1H exchangeable with D_2_O), 7.59 (d, *J*=8.5 Hz, 1H), 7.24–7.18 (m, 1H), 6.92–6.82 (m, 1H), 6.58 (s, 2H), 2.01 (s, 6H). ^13^C NMR (101 MHz, DMSO‐*d*_6_) *δ* 158.00, 142.62, 121.73, 120.55, 119.65, 118.75, 117.79, 114.23, 111.41, 19.93. HRMS (ESI): *m/z* [M+H]^+^ calcd. for C_20_H_18_N_4_O_3_S+H^+^: 395.1172. Found: 395.1163.

**2‐(4‐hydroxyphenyl)‐*N*‐(pyridin‐2‐yl)‐1*H*‐benzo[*d*]imidazole‐6‐sulfonamide (9 l)**. Compound **9 l** (181 mg, 65 %) was obtained as a white solid from derivative **16 a** (200 mg, 0.76 mmol) and 4‐hydroxybenzaldehyde **17 d** (93 mg, 0.76 mmol) according to the procedure described for **9 a**. ^1^H NMR (400 MHz, DMSO‐*d*_6_) *δ* 13.04 (s, 1H, exchangeable with D_2_O), 10.06 (s, 1H, exchangeable with D_2_O), 8.10–8.05 (m, 1H), 8.03–7.96 (m, 4H), 7.71–7.64 (m, 2H), 7.61–7.56 (m, 1H, exchangeable with D_2_O), 7.17 (d, *J*=8.6 Hz, 1H), 6.92 (d, *J*=8.7 Hz, 2H), 6.89–6.82 (m, 1H). ^13^C NMR (101 MHz, DMSO‐*d*_6_) *δ* 159.73, 128.55, 120.27, 115.80. HRMS (ESI): *m/z* [M+H]^+^ calcd. for C_18_H_14_N_4_O_3_S+H^+^: 367.0859. Found: 367.0848.

**2‐(3‐hydroxyphenyl)‐*N*‐(pyridin‐2‐yl)‐1*H*‐benzo[*d*]imidazole‐6‐sulfonamide (9 m)**. Compound **9 m** (270 mg, 65 %) was obtained as a white solid from derivative **16 a** (300 mg, 1.13 mmol) and 3‐hydroxybenzaldehyde **17 e** (138 mg, 1.13 mmol) according to the procedure described for **9 a**. ^1^H NMR (400 MHz, DMSO‐*d_6_*) *δ* 9.91 (s, 1H, exchangeable with D_2_O), 8.19–8.11 (m, 1H), 8.04–7.98 (m, 1H), 7.80 (dd, *J*=8.6, 1.7 Hz, 1H), 7.77–7.74 (m, 1H), 7.73–7.66 (m, 1H), 7.62–7.55 (m, 2H), 7.45–7.37 (m, 1H), 7.20 (d, *J*=8.6 Hz, 1H), 7.01–6.98 (m, 1H), 6.90–6.83 (m, 1H).^13^C NMR (101 MHz, DMSO‐*d*_6_) *δ* 157.92, 153.49, 152.99, 140.24, 130.39, 128.86, 121.55, 118.53, 117.87, 113.87, 113.58. HRMS (ESI): *m/z* [M+H]^+^ calcd. for C_18_H_14_N_4_O_3_S+H^+^: 367.0859. Found: 367.0847.

**2‐(4‐chlorophenyl)‐*N*‐(pyridin‐2‐yl)‐1*H*‐benzo[*d*]imidazole‐6‐sulfonamide (9 n)**. Compound **9 n** (136 mg, 52 %) was obtained as a pale‐yellow solid from derivative **16 a** (180 mg, 0.68 mmol) and 4‐chlorobenzaldehyde **17 f** (96 mg, 0.68 mmol) according to the procedure described for **9 a**. ^1^H NMR (400 MHz, DMSO‐*d*_6_) *δ* 8.18 (d, *J*=8.6 Hz, 2H), 8.13–8.12 (m, 1H), 8.04–8.01 (m, 1H), 7.77–7.73 (m, 2H), 7.72–7.69 (m, 1H), 7.67 (d, *J*=8.6 Hz, 2H), 7.22–7.16 (m, 1H), 6.91–6.84 (m, 1H). ^13^C NMR (101 MHz, DMSO‐*d*_6_) *δ* 152.88, 139.94, 135.36, 129.23, 128.54, 128.03, 121.02, 113.36. HRMS (ESI): *m/z* [M+H]^+^ calcd. for C_18_H_13_ClN_4_O_2_S+H^+^: 385.0521. Found: 385.0509.

**2‐(3,4‐dihydroxyphenyl)‐*N*‐(pyridin‐2‐yl)‐1*H*‐benzo[*d*]imidazole‐6‐sulfonamide (9 o)**. Compound **9 o** (255 mg, 59 %) was obtained as a pale‐yellow solid from derivative **16 a** (300 mg, 1.14 mmol) and 3,4‐dihydroxybenzaldehyde **17 g** (157 mg, 1.14 mmol) according to the procedure described for **9 a**. ^1^H NMR (400 MHz, DMSO‐*d*_6_) *δ* 10.01 (s, 1H, exchangeable with D_2_O), 9.52 (s, 1H, exchangeable with D_2_O), 8.12–8.07 (m, 1H), 8.04–7.98 (m, 1H), 7.81 (dd, *J*=8.5, 1.7 Hz, 1H), 7.78–7.67 (m, 2H), 7.58 (d, *J*=2.2 Hz, 1H), 7.51 (dd, *J*=8.3, 2.2 Hz, 1H), 7.20 (d, *J*=8.6 Hz, 1H), 6.97 (d, *J*=8.3 Hz, 1H), 6.91–6.83 (m, 1H). ^13^C NMR (101 MHz, DMSO‐*d*_6_) *δ* 153.45, 153.09, 149.75, 145.95, 140.43, 121.86, 119.82, 116.12, 115.71, 114.68, 114.35, 113.71, 113.29. HRMS (ESI): *m/z* [M+H]^+^ calcd. for C_18_H_14_N_4_O_4_S+H^+^: 383.0809. Found: 383.0796.

**2‐(2,4‐dihydroxyphenyl)‐*N*‐(pyridin‐2‐yl)‐1*H*‐benzo[*d*]imidazole‐6‐sulfonamide (9 p)**. Compound **9 p** (260 mg, 60 %) was obtained as a light‐yellow solid from derivative **16 a** (300 mg, 1.14 mmol) and 2,4‐dihydroxybenzaldehyde **17 h** (157 mg, 1.14 mmol) according to the procedure described for **9 a**. ^1^H NMR (400 MHz, DMSO‐*d*_6_) 10.26 (s, 1H, exchangeable with D_2_O), 8.15–8.10 (m, 1H), 8.04–7.98 (m, 1H), 7.87 (d, *J*=8.6 Hz, 1H), 7.77 (d, *J*=8.6 Hz, 1H), 7.74–7.67 (m, 3H, 1H exchangeable with D_2_O), 7.19 (d, *J=*8.6 Hz, 1H), 6.89–6.83 (m, 1H), 6.51–6.43 (m, 2H). ^13^C NMR (101 MHz, DMSO‐*d*_6_) *δ* 162.03, 159.80, 140.27, 128.77, 121.43, 113.63, 108.28, 103.02. HRMS (ESI): *m/z* [M+H]^+^ calcd. for C_18_H_14_N_4_O_4_S+H^+^: 383.0809. Found: 383.0794.

***N*** **‐(*tert*‐butyl)‐2‐(4‐hydroxy‐3,5‐dimethylphenyl)‐1*H*‐benzo[*d*]imidazole‐6‐sulfonamide (10)**. Compound **10** (294 mg, 64 %) was obtained as a light‐yellow solid from derivative **16 i** (300 mg, 1.23 mmol) and 4‐hydroxy‐3,5‐dimethylbenzaldehyde **17 a** (185 mg, 1.23 mmol) according to the procedure described for **9 a**. ^1^H NMR (400 MHz, DMSO‐*d*_6_) *δ* 12.95 (s, 1H, exchangeable with D_2_O), 8.85 (s, 1H, exchangeable with D_2_O), 8.00–7.92 (m, 1H), 7.79 (s, 2H), 7.65–7.61 (m, 2H, 1H, exchangeable with D_2_O), 7.44–7.40 (m, 1H), 2.27 (s, 6H), 1.08 (s, 9H). MS (ESI) *m/z*: 374 (M+H)^+^.

**4‐amino‐3‐nitro‐*N*‐(pyridin‐2‐yl)benzenesulfonamide (15 a)**. To a cooled stirred solution of 4‐amino‐3‐nitrobenzenesulfonyl chloride **13** (1.41 g, 5.98 mmol) in dry pyridine (6 mL), 2‐aminopyridine **14 a** (506 mg, 5.38 mmol) was added portion wise, under N_2_ atmosphere. The reaction was kept at 0 °C until disappearance of the starting material (monitored by TLC). Then, water (10 mL) was added: the resulting solid was filtered and washed with water to afford the title compound (633 mg, 40 %) as an orange solid. ^1^H NMR (400 MHz, DMSO‐*d*_6_) *δ* 8.49–8.39 (m, 1H), 8.09–8.00 (m, 1H), 7.94 (s, 2H, exchangeable with D_2_O), 7.78–7.69 (m, 2H), 7.13–7.09 (m, 2H), 6.91–6.83 (m, 1H). MS (ESI) *m/z*: 295 (M+H)^+^.

**4‐amino‐*N*‐(4‐cyanophenyl)‐3‐nitrobenzenesulfonamide (15 b)**. Compound **15 b** (273 mg, 35 %) was obtained as an orange solid from derivative **13** (638 mg, 2.69 mmol) and 4‐aminobenzonitrile **14 b** (290 mg, 2.45 mmol) according to the procedure described for **15 a**. ^1^H NMR (400 MHz, DMSO‐*d_6_*) *δ* 10.92 (s, 1H, exchangeable with D_2_O), 8.40 (d, *J*=2.3 Hz, 1H), 8.05 (s, 2H, exchangeable with D_2_O), 7.71 (d, *J*=8.8 Hz, 2H), 7.67 (dd, *J*=9.0, 2.3 Hz, 1H), 7.24 (d, *J*=8.8 Hz, 2H), 7.08 (d, *J*=9.0 Hz, 1H). MS (ESI) *m/z*: 319 (M+H)^+^.

**4‐amino‐*N*‐methyl‐3‐nitrobenzenesulfonamide (15 c)**. To a cooled stirred solution of **13** (380 mg, 1.61 mmol) in dry THF (20 mL), was added dropwise, under N_2_ atmosphere, methylamine **14 c** (2 M solution in THF, 3.22 mL, 6.44 mmol) and the reaction was stirred at room temperature for 18 h. Then, the reaction mixture was concentrated under reduced pressure, taken up with water (50 mL) and extracted with EtOAc (3×20 mL). The combined organic phases were washed with brine (10 mL), dried, filtered and evaporated under reduced pressure. The title compound (260 mg, 70 %) was obtained as a pale‐yellow solid after crystallization from EtOH. ^1^H NMR (400 MHz, DMSO‐*d*_6_) *δ* 8.36–8.29 (m, 1H), 7.99 (s, 2H, exchangeable with D_2_O), 7.66 (dd, *J*=9.0, 2.2 Hz, 1H), 7.38–7.34 (m, 1H, exchangeable with D_2_O), 7.14 (d, *J*=9.0 Hz, 1H), 2.39 (d, *J*=4.8 Hz, 3H). MS (ESI) *m/z*: 232 (M+H)^+^.

**4‐amino‐3‐nitro‐*N*‐(pyridin‐2‐ylmethyl)benzenesulfonamide (15 d)**. Compound **15 d** (600 mg, 60 %) was obtained as a yellow solid from derivative **13** (767 mg, 3.24 mmol) and 2‐picolylamine **14 d** (1.33 mL, 12.93 mmol) according to the procedure described for **15 c**. ^1^H NMR (400 MHz, DMSO‐*d*_6_) *δ* 8.40–8.38 (m, 1H), 8.26 (d, *J*=2.2 Hz, 1H), 8.21–8.14 (m, 1H, exchangeable with D_2_O), 7.95 (s, 2H, exchangeable with D_2_O), 7.72–7.61 (m, 2H), 7.33 (d, *J*=7.9 Hz, 1H), 7.23–7.18 (m, 1H), 7.06 (d, *J*=9.0 Hz, 1H), 4.09 (s, 2H). MS (ESI) *m/z*: 309 (M+H)^+^.

**4‐amino‐3‐nitro‐*N*‐(pyridin‐3‐ylmethyl)benzenesulfonamide (15 e)**. Compound **15 e** (566 mg, 62 %) was obtained as a yellow solid from derivative **13** (700 mg, 2.96 mmol) and the 3‐picolylamine **14 e** (1.20 mL, 11.83 mmol) according to the procedure described for **15 c**. ^1^H NMR (400 MHz, DMSO‐*d*_6_) *δ* 8.42–8.37 (m, 1H), 8.28 (d, *J*=2.2 Hz, 1H), 8.16 (s, 1H, exchangeable with D_2_O), 7.97 (s, 2H, exchangeable with D_2_O), 7.66–7.61 (m, 2H), 7.30–7.24 (m, 1H), 7.07 (d, *J*=9.0 Hz, 1H), 4.03 (s, 2H). MS (ESI) *m/z*: 309 (M+H)^+^.

**4‐amino‐*N*‐cyclopropyl‐3‐nitrobenzenesulfonamide (15 f)**. Compound **15 f** (450 mg, 85 %) was obtained as a yellow solid from derivative **13** (487 mg, 2.06 mmol) and cyclopropylamine **14 f** (0.57 mL, 8.25 mmol) according to the procedure described for **15 c**. ^1^H NMR (400 MHz, DMSO‐*d*_6_) *δ* 8.37 (d, *J*=2.2 Hz, 1H), 8.00 (s, 2H, exchangeable with D_2_O), 7.83–7.77 (m, 1H, exchangeable with D_2_O), 7.68 (dd, *J*=9.0, 2.2 Hz, 1H), 7.15 (d, *J=*9.0 Hz, 1H), 2.16–2.08 (m, 1H), 0.53–0.46 (m, 2H), 0.39–0.34 (m, 2H). MS (ESI) *m/z*: 258 (M+H)^+^.

**4‐amino‐*N*‐cyclopentyl‐3‐nitrobenzenesulfonamide (15 g)**. Compound **15 g** (393 mg, 70 %) was obtained as an orange solid from derivative **13** (466 mg, 1.97 mmol) and cyclopentylamine **14 g** (0.78 mL, 7.90 mmol) according to the procedure described for **15 c**. ^1^H NMR (400 MHz, DMSO‐*d*_6_) *δ* 8.36 (d, *J*=2.2 Hz, 1H), 7.97 (s, 2H, exchangeable with D_2_O), 7.68 (dd, *J*=9.0, 2.2 Hz, 1H), 7.57–7.52 (m, 1H, exchangeable with D_2_O), 7.13 (d, *J*=9.0 Hz, 1H), 3.38–3.35 (m, 1H), 1.66–1.48 (m, 4H), 1.42–1.25 (m, 4H). MS (ESI) *m/z*: 286 (M+H)^+^.

**4‐amino‐*N*‐cyclohexyl‐3‐nitrobenzenesulfonamide (15 h)**. Compound **15 h** (956 mg, 63 %) was obtained as a yellow solid from derivative **13** (1.20 g, 5.07 mmol) and cyclohexylamine **14 h** (2.32 mL, 20.28 mmol) according to the procedure described for **15 c**. ^1^H NMR (400 MHz, DMSO‐*d*_6_) *δ* 8.36 (d, *J*=2.2 Hz, 1H), 7.96 (s, 2H, exchangeable with D_2_O), 7.70 (dd, *J*=9.0, 2.2 Hz, 1H), 7.59–7.54 (m, 1H, exchangeable with D_2_O), 7.12 (d, *J*=9.0 Hz, 1H), 2.94–2.85 (m, 1H), 1.62–1.52 (m, 5H), 1.19–1.09 (m, 5H). MS (ESI) *m/z*: 300 (M+H)^+^.

**4‐amino‐*N*‐(*tert*‐butyl)‐3‐nitrobenzenesulfonamide (15 i)**. Compound **15 i** (353 mg, 73 %) was obtained as an orange solid from derivative **13** (420 mg, 1.77 mmol) and the *tert*‐butylamine **14 i** (0.75 mL, 7.09 mmol) according to the procedure described for **15 c**. ^1^H NMR (400 MHz, DMSO‐*d*_6_) *δ* 8.38 (d, *J*=2.2 Hz, 1H), 7.95 (s, 2H, exchangeable with D_2_O), 7.70 (dd, *J*=9.0, 2.2 Hz, 1H), 7.44 (s, 1H, exchangeable with D_2_O), 7.11 (d, *J*=9.0 Hz, 1H), 1.10 (s, 9H). MS (ESI) *m/z*: 274 (M+H)^+^.

**3,4‐diamino‐*N*‐(pyridin‐2‐yl)benzenesulfonamide (16 a)**. To a solution of compound **15 a** (440 mg, 1.50 mmol) in 7.2 mL of glacial AcOH, Zn dust (977 mg, 15.0 mmol) was added portion wise. The reaction mixture was stirred at room temperature until disappearance of the starting material (monitored by TLC). Then, the insoluble salts were filtered, and the filtrate was concentrated under reduced pressure. The crude material was taken up with water (20 mL), and the aqueous phase was extracted with EtOAc (3×15 mL). The organic phases were collected and washed with saturated solution of NaHCO_3_ (3×15 mL) and brine (15 mL), dried, filtered and concentrated in vacuo to obtain the title compound (254 mg, 64 %) as a light‐brown solid. ^1^H NMR (400 MHz, DMSO‐*d_6_*) *δ* 10.86 (s, 1H, exchangeable with D_2_O), 8.13–8.04 (m, 1H), 7.68–7.58 (m, 1H), 7.07 (d, *J*=8.5 Hz, 1H), 7.02–6.97 (m, 1H), 6.96–6.85 (m, 2H), 6.48 (d, *J*=8.2 Hz, 1H), 5.23 (brs, 2H, exchangeable with D_2_O), 4.82 (brs, 2H, exchangeable with D_2_O). MS (ESI) *m/z*: 265 (M+H)^+^.

**3,4‐diamino‐*N*‐(4‐cyanophenyl)benzenesulfonamide (16 b)**. Compound **16 b** was obtained as a pale‐yellow solid (75 mg, 84 %) from derivative **15 b** (100 mg, 0.31 mmol) according to the procedure described for **16 a**. ^1^H NMR (400 MHz, DMSO‐*d_6_*) *δ* 10.59 (s, 1H, exchangeable with D_2_O), 7.65 (d, *J*=8.4 Hz, 2H), 7.18 (d, *J=*8.4 Hz, 2H), 6.95–6.86 (m, 2H), 6.50 (d, *J*=8.1 Hz, 1H), 5.36 (brs, 2H, exchangeable with D_2_O), 4.89 (brs, 2H, exchangeable with D_2_O). MS (ESI) *m/z*: 289 (M+H)^+^.

**3,4‐diamino‐*N*‐methylbenzenesulfonamide (16 c)**. Pd/C (10 wt% on activated carbon, 0.1 equiv) was added to a solution of **15 c** (100 mg, 0.43 mmol) in EtOAc (4.3 mL) and the reaction was stirred under H_2_ (1 atm, balloon) for 18 h. The reaction mixture was filtered and concentrated to give the title compound (80 mg, 92 %) as a yellow solid. ^1^H NMR (400 MHz, DMSO‐*d*_6_) *δ* 6.90 (d, *J*=2.2 Hz, 1H), 6.83–6.78 (m, 2H, 1H exchangeable with D_2_O), 6.55 (d, *J*=8.1 Hz, 1H), 5.19 (brs, 2H, exchangeable with D_2_O), 4.83 (brs, 2H, exchangeable with D_2_O), 2.32 (d, *J*=5.2 Hz, 3H). MS (ESI) *m/z*: 202 (M+H)^+^.

**3,4‐diamino‐*N*‐(pyridin‐2‐ylmethyl)benzenesulfonamide (16 d)**. Compound **16 d** (250 mg, 92 %) was obtained as a light‐brown solid from derivative **15 d** (300 mg, 0.97 mmol) according to the procedure described for **16 c**. ^1^H NMR (400 MHz, DMSO‐*d*_6_) *δ* 8.44 (d, *J*=4.7 Hz, 1H), 7.78–7.70 (m, 1H), 7.60 (t, *J*=6.2 Hz, 1H exchangeable with D_2_O), 7.39 (d, *J*=7.6 Hz, 1H), 7.24 (dd, *J*=7.6, 4.7 Hz, 1H), 6.96 (d, *J*=2.4 Hz, 1H), 6.86 (dd, *J*=8.1, 2.4 Hz, 1H), 6.55 (d, *J*=8.1 Hz, 1H), 5.22 (brs, 2H, exchangeable with D_2_O), 4.84 (brs, 2H, exchangeable with D_2_O), 3.96 (d, *J*=6.2 Hz, 2H). MS (ESI) *m/z*: 279 (M+H)^+^.

**3,4‐diamino‐*N*‐(pyridin‐3‐ylmethyl)benzenesulfonamide (16 e)**. Compound **16 e** (85 mg, 94 %) was obtained as a yellow solid from derivative **15 e** (100 mg, 0.32 mmol) according to the procedure described for **16 c**. ^1^H NMR (400 MHz, DMSO‐*d*_6_) *δ* 8.46–8.36 (m, 1H), 7.69–7.57 (m, 2H), 7.34–7.26 (m, 1H), 6.95 (d, *J*=2.2 Hz, 1H), 6.86 (dd, *J*=8.2, 2.2 Hz, 1H), 6.55 (d, *J*=8.2 Hz, 1H), 5.23 (brs, 2H, exchangeable with D_2_O), 4.84 (brs, 2H, exchangeable with D_2_O), 3.90 (d, *J*=6.3 Hz, 2H). MS (ESI) *m/z*: 279 (M+H)^+^.

**3,4‐diamino‐*N*‐cyclopropylbenzenesulfonamide (16 f)**. Compound **16 f** (343 mg, 97 %) was obtained as brown oil from derivative **15 f** (400 mg, 1.55 mmol) according to the procedure described for **16 c**. ^1^H NMR (400 MHz, DMSO‐*d*_6_) *δ* 7.34–7.29 (m, 1H, exchangeable with D_2_O), 6.94 (d, *J*=2.1 Hz, 1H), 6.85 (dd, *J*=8.2, 2.1 Hz, 1H), 6.55 (d, *J*=8.2 Hz, 1H), 5.20 (brs, 2H, exchangeable with D_2_O), 4.82 (brs, 2H, exchangeable with D_2_O), 2.04–1.99 (m, 1H), 0.46–0.32 (m, 4H). MS (ESI) *m/z*: 228 (M+H)^+^.

**3,4‐diamino‐*N*‐cyclopentylbenzenesulfonamide (16 g)**. Compound **16 g** (320 mg, 92 %) was obtained as an orange solid from derivative **15 g** (387 mg, 1.36 mmol) according to the procedure described for **16 c**. ^1^H NMR (400 MHz, DMSO‐*d*_6_) *δ* 7.00–6.96 (m, 1H, exchangeable with D_2_O), 6.92 (d, *J*=2.2 Hz, 1H), 6.83 (dd, *J*=8.1, 2.2 Hz, 1H), 6.53 (d, *J*=8.1 Hz, 1H), 5.16 (brs, 2H, exchangeable with D_2_O), 4.83 (brs, 2H, exchangeable with D_2_O), 3.29–3.21 (m, 1H), 1.63–1.46 (m, 4H), 1.40–1.24 (m, 4H). MS (ESI) *m/z*: 256 (M+H)^+^.

**3,4‐diamino‐*N*‐cyclohexylbenzenesulfonamide (16 h)**. Compound **16 h** (420 mg, 94 %) was obtained as a light‐brown solid from derivative **15 h** (496 mg, 1.66 mmol) according to the procedure described for **16 c**. ^1^H NMR (400 MHz, DMSO‐*d*_6_) *δ* 7.01–6.96 (m, 1H, exchangeable with D_2_O), 6.92 (d, *J*=2.2 Hz, 1H), 6.83 (dd, *J*=8.1, 2.2 Hz, 1H), 6.52 (d, *J*=8.1 Hz, 1H), 5.14 (brs, 2H, exchangeable with D_2_O), 4.80 (brs, 2H, exchangeable with D_2_O), 2.84–2.76 (m, 1H), 1.62–1.39 (m, 5H), 1.14–0.94 (m, 5H). MS (ESI) *m/z*: 270 (M+H)^+^.

**3,4‐diamino‐*N*‐(*tert*‐butyl)benzenesulfonamide (16 i)**. Compound **16 i** (223 mg, 89 %) was obtained as an orange solid from derivative **15 i** (283 mg, 1.03 mmol) according to the procedure described for **16 c**. ^1^H NMR (400 MHz, DMSO‐ *d*_6_) δ 6.96 (d, *J*=2.1 Hz, 1H), 6.90–6.84 (m, 2H, 1H exchangeable with D_2_O), 6.53 (d, *J*=8.2 Hz, 1H), 5.13 (brs, 2H, exchangeable with D_2_O), 4.79 (brs, 2H, exchangeable with D_2_O), 1.08 (s, 9H). MS (ESI) *m/z*: 244 (M+H)^+^.

**Solubility determination**. Solubility of the compounds was determined using Nepheloskan Ascent® (Labsystems). The experiments were performed at room temperature in 96‐well plates in a final volume of 300 μL. Each compound was tested in quadruplicate at the concentrations of 50 and 100 μM in PBS with 1 % DMSO. The measurements were performed at 4 different time points (T0, 30 min, 60 min, 90 min) from the preparation of the samples. Data obtained were compared to control (PBS with 1 % DMSO) and the ratio sample/control was determined for each compound. Compounds are considered soluble if the ratio is≤3.

**Parallel Artificial Membrane Permeability Assay (PAMPA)**. Donor solution (0.2 mM) was prepared by diluting 20 mM dimethyl sulfoxide (DMSO) compound stock solution using phosphate buffer (pH 7.4, 0.01 M). Filters were coated with 5 μL of a 1 % (w/v) dodecane solution of L‐α‐phosphatidylcholine. Donor solution (150 μL) was added to each well of the filter plate. To each well of the acceptor plate, 300 μL of solution (5 % DMSO in phosphate buffer) was added. Selected compounds were tested in triplicate, propranolol and furosemide were used as positive and negative controls, respectively. The sandwich was incubated for 24 h at room temperature under gentle shaking. After the incubation time, the sandwich plates were separated and 250 μL of the acceptor plate was transferred to a UV quartz microtiter plate and measured by UV spectroscopy, using a Multiskan GO microplate spectrophotometer (Thermo Fisher Scientific) at 250–500 nm at a step of 5 nm. Reference solutions (250 μL) were prepared diluting the sample stock solutions to the same concentration as that with no membrane barrier. The apparent permeability value P_app_ is determined from the ratio r of the absorbance of compound found in the acceptor chamber divided by the theoretical equilibrium absorbance (determined independently), the Faller^[32]^ modification of Sugano^[33]^ equation:

$$P_{app}=-\frac{V_{D}V_{R}}{\left(V_{D}+V_{R}\right)At}\times ln\left(1-r\right)$$

In this equation, *V_R_* is the volume of the acceptor compartment (0.3 cm^3^), *V_D_* is the donor volume (0.15 cm^3^), *A* is the accessible filter area (0.24 cm^2^), and *t* is the incubation time in seconds.

### In Vitro Drug Metabolism Using Liver Microsomes

*Instrumentation and chromatographic conditions*. The metabolic stability of investigated compounds was monitored using a Nexera UHPLC system (Shimadzu, Kyoto, Japan) consisting of a CBM‐20A controller, two LC‐30AD pumps, an SPD‐M20A photo diode array detector, a CTO‐20AC column oven and, a SIL‐30AC autosampler. The chromatographic analyses were accomplished on a Kinetex® Evo C18 column, 150×2.1 mm×2.6 μm (Phenomenex®, Bologna, Italy) maintained at 40 °C. The optimal mobile phase consisted of 0.1 % HCOOH/H_2_O v/v (A) and 0.1 % HCOOH/ACN v/v (B) delivered at constant flow rate of 0.5 mL/min ^−1^. Analysis was performed in gradient elution as follows: 0–8.00 min, 5–95 % B; 8.00–10.00 min, isocratic to 95 % B; then five minutes for column re‐equilibration. Data acquisition was set in the range 190–800 nm and chromatograms were monitored at 254 nm.

*In vitro drug metabolism using human liver microsomes*. 25 μL of 5 mg/mL human (CD‐1) microsomes (Thermo Fisher Scientific, Bremen, Germany) were pre‐incubated with 0.625 μL of 100 μg/mL alamethicin. Then 2.5 μL of sample (5 mM) with 168 μL of 100 mM phosphate buffer (pH 7.4), 4 μL of 500 mM MgCl_2_ were added, the mixture was incubated at 37 °C for 5 min. Optimal UGT activity can be achieved by the addition of MgCl_2_ and alamethicin as pore forming antibiotic. These components allow for the efficient transfer of a glucuronide product and the cofactor uridine 5′‐diphospho‐α‐D‐glucuronic acid (UDPGA) within the microsomal matrix. The reaction has started by adding 50 μL of mix NADPH 20 mM and UDP‐GlcUA 20 mM as cofactors (1 : 1 v/v) and carried out 37 °C for 60 min in a Thermomixer comfort (Eppendorf, Hamburg, Germany). The reaction was stopped by the addition of 200 μL ice‐cold acetonitrile and then samples were centrifuged at 14,000 rpm at 25 °C for 5 min (Eppendorf® microcentrifuge 5424, Hamburg, Germany). The supernatants were collected and injected in UHPLC. The control at 0 min was obtained by addition of ice‐cold acetonitrile immediately after incubation with microsomes. Testosterone was used as positive control while the negative control was prepared by incubation up to 60 min without UDP‐GlcUA and NADPH cofactors.

**Protein Expression and Purification**. Colonies from freshly transformed plasmid DNA in competent *E. coli* BL21(DE3)‐R3‐pRARE2 cells (phage‐resistant derivative of BL21(DE3) strain), with a pRARE plasmid encoding rare codon tRNAs, were grown overnight at 37 °C in 5 mL of Luria‐Bertani medium (LB‐broth, Merck) with 50 μg/mL kanamycin and 34 μg/mL chloramphenicol (start‐up culture). The start‐up culture was diluted 1 : 1000 in fresh medium and cell growth was allowed at 37 °C to an optical density of about 0.5 (OD600) before the temperature was decreased to 18 °C. When the system equilibrated at 18 °C the optical density was about 0.8 (OD600) and protein expression was induced over night at 18 °C with 0.1 mM isopropyl‐β‐*d*‐thiogalactopyranoside (IPTG). The bacteria were harvested by centrifugation (8,700×g for 15 min at 4 °C, JLA 81,000 rotor, on a Beckman Coulter Avanti J‐20 XP centrifuge) and were frozen at −20 °C as pellets for storage. Cells expressing His6‐tagged proteins were re‐suspended in lysis buffer (50 mM HEPES, pH 7.5 at 25 °C, 500 mM NaCl, 5 mM Imidazole, 5 % glycerol and 0.5 mM TCEP (Tris(2‐carboxyethyl)phosphine hydrochloride)) in the presence of protease inhibitor cocktail (1 μL/mL) and lysed using an EmulsiFlex‐C5 high pressure homogenizer (Avestin – Mannheim, Germany) at 4 °C. 0.15 % of PEI (polyethyleneimine) was added for 30 min on ice and the lysate was cleared by centrifugation (16,000×g for 1 h at 4 °C, JA 25.50 rotor, on a Beckman Coulter Avanti J‐20 XP centrifuge) and was applied to a nickel‐nitrilotriacetic acid agarose column (NiNTA, Qiagen Ltd., 5 mL, equilibrated with 20 mL lysis buffer). The column was washed once with 30 mL of lysis buffer then twice with 10 mL of lysis buffer containing 30 mM Imidazole. The protein was eluted using a step elution of Imidazole in lysis buffer (50, 100, 150, 250 mM Imidazole in 50 mM HEPES, pH 7.5 at 25 °C, 500 mM NaCl). All fractions were collected and monitored by SDS‐polyacrylamide gel electrophoresis (BioRad Criterion™ Precast Gels, 4–12 % Bis‐Tris, 1.0 mm, from Bio‐Rad, CA.). After the addition of 10 mM dithiothreitol (DTT), the eluted protein was treated overnight at 4 °C with Tobacco Etch Virus (TEV) protease to remove the hexa‐histidine tag. The protein was further purified with size exclusion chromatography on a Superdex 75 16/60 HiLoad gel filtration column (GE/Amersham Biosciences) on an ÄktaPrime™ plus system (GE/Amersham Biosciences). Samples were monitored by SDS‐polyacrylamide gel electrophoresis and concentrated to 10–40 mg/mL in the gel filtration buffer, 10 mM HEPES pH 7.5, 150 mM NaCl, 0.5 mM TCEP. Samples for isothermal calorimetry were dialysed over night at 4 °C in a D‐Tube™ Dialyser Midi, MWCO 3.5 kDa to a final buffer of 50 mM HEPES, pH 7.4 (at 25 °C), 150 mM NaCl. Protein handling was carried out on ice or in a cold room in all the above steps.

**Protein stability shift assay**. Thermal melting experiments were carried out using an Mx3005p Real Time PCR machine (Stratagene). Proteins were buffered in 10 mM HEPES pH 7.5, 500 mM NaCl and assayed in a 96‐well plate at a final concentration of 2 μM in 20 μL volume. Compounds were added at a final concentration of 10 μM. SYPRO Orange (Molecular Probes) was added as a fluorescence probe at a dilution of 1 : 1000. Excitation and emission filters for the SYPRO‐Orange dye were set to 465 nm and 590 nm, respectively. The temperature was raised with a step of 3 °C per minute from 25 °C to 96 °C and fluorescence readings were taken at each interval. The temperature dependence of the fluorescence during the protein denaturation process was approximated by the equation

$$y\left(T\right)=y_{F}+\frac{y_{U}-y_{F}}{1+e^{\Delta uG_{\left(T\right)}/RT}}$$

where ΔuG_(T)_ is the difference in unfolding free energy between the folded and unfolded state, R is the gas constant and y_F_ and y_U_ are the fluorescence intensity of the probe in the presence of completely folded and unfolded protein, respectively. The baselines of the denatured and native states were approximated by a linear fit. The observed temperature shifts, ΔT_m obs_, were recorded as the difference between the transition midpoints of sample and reference wells containing protein without ligand in the same plate and determined by non‐linear least squares fit.

**Isothermal Titration Calorimetry**. Experiments were carried out on an ITC200 titration microcalorimeter from MicroCal™, LLC (Northampton, MA) with a cell volume of 0.200 mL and a 40 μL microsyringe. All experiments were carried out at 15 °C while stirring at 750 rpm, in ITC buffer (20 mM HEPES pH 7.5 (at 25 °C), 150 mM NaCl). The microsyringe was loaded with a solution of BRD4 BD1 (280 μM in ITC buffer) and the cell was loaded with 25 μM of the compound (**9 a**). Following baseline equilibration an additional delay of 60 s was applied. All titrations were conducted using an initial injection of 0.3 μL followed by 38 identical injections of 1 μL with a duration of 2 s (per injection) and a spacing of 120 s between injections. The heat of dilution was determined by independent titrations (protein into buffer) and was subtracted from the experimental data. The collected data were implicated in the MicroCal™ Origin software supplied with the instrument to yield enthalpies of binding (ΔH) and binding constants (K_B_) as previously described by Wiseman and co‐workers.^[34]^ Thermodynamic parameters were calculated (ΔG=ΔH−TΔS=−RTlnK_B_, where ΔG, ΔH and ΔS are the changes in free energy, enthalpy and entropy of binding respectively). A single binding site model was employed

**TR‐FRET assay**. Compound **9 a** was tested on BD1 and BD2 domains (6×His‐tagged) of each BET protein in a dose‐response format measuring binding competition between the compounds and an AlexaFluor 647 derivative of **1**. Compound was diluted in assay buffer (150 mM HEPES, 150 mM NaCl, 5 % glycerol, 1 mM DTT, and 1 mM CHAPS, pH 7.4), starting from a stock solution of 10 mM (100 % DMSO). The highest concentration tested was 100 μM and from this concentration 11 three‐fold dilutions were prepared. 5 μL of each dilution was transferred into a low volume black 384‐well plate and, taking advance of a Thermo Scientific Multidrop Combi, 2 μL of protein (10 nM), 2 μL of Alexa Fluor 647 derivative of **1** (50 nM) and 1 μL of europium chelate‐labeled anti‐6His antibody (1 nM) were transferred in each well. After an equilibration of 30 min in the dark at room temperature, the binding of the protein to the fluorescent ligand was detected on BMG Labtech Pherastar luminescence plate reader (excitation=337 nm; emission 1=615 nm; emission 2=665 nm; dual wavelength bias dichroic=400 nm, 630 nm). TR‐FRET ratio was calculated using the following equation:

$$ratio=\frac{acceptor\;fluorescence\;at\;665\;\;nm}{donor\;fluorescence\;at\;615\;\;nm}\times 1000$$

Compound was tested in triplicate and data were analyzed using GraphPad Prism software (version 8.0) using the equation log(inhibitor) vs. normalized response‐variable slope.

**Cell Viability Assay**. HeLa cell line was cultured in DMEM supplemented with 10 % (v/v) fetal bovine serum, 100 U/mL penicillin, 100 μg/mL streptomycin, and 2 mM L‐glutamine at 37 °C in a 5 % CO_2_ atmosphere. Cell viability was determined using a 3‐(4,5‐dimethylthiazol‐2‐yl)‐2,5‐diphenyltetrazolium bromide (MTT) assay. A total of 200 μL of cells (5×10^4^ cells/mL for 24 h, 3×10^4^ cells/mL for 48 h, 2.5×10^4^ cells/mL for 72 h) seeded in 96‐well plates were exposed for 24, 48 and 72 h to different concentrations of compounds **7 a** and **9 a** (0.5, 5 and 50 μM) in media containing 1 % DMSO. The mitochondrial dependent reduction of MTT to formazan was used to assess cell viability. Live cells reduce yellow MTT to purple formazan. The formazan was solubilized in DMSO, and absorbance was measured at 550 nm and corrected for 620 nm background. Experiments were performed in quadruplicate and all values are expressed as the percentage of the control containing 1 % DMSO.

**Western‐blot experiments**. HeLa cells were seeded (sub‐confluent) in 6‐well plates and treated with compounds **7 a** and **9 a** at the concentration of 50 μM (1 % DMSO) for 24, 48 and 72 h. After the incubation with the compounds, cells were harvested, washed with 1X PBS and resuspended in 100 μL of RIPA buffer supplemented with a 1X protease inhibitors cocktail and 1 mM PMSF, keeping the samples at 4 °C. Cells were lysed by sonication on ice (1 min, 30 % Amplitude), the samples were centrifuged at 12,000×g for 30 min at 4 °C to separate the lysate from debris. Protein concentration for each sample was determined using Bradford assay and 10 μg of lysate was loaded onto a 10 % acrylamide gel for electrophoretic separation at 200 V. Proteins were then transferred onto a nitrocellulose membrane for 1 h at 75 V. After the transfer, the membrane was blocked with 5 % milk (dissolved in TBS with 0.05 % Tween) for 1 h and then incubated with primary antibodies anti‐c‐Myc (ab32072) and anti‐GAPDH (ab8245) at 4 °C overnight. Membrane was washed with TBS‐Tween 0.05 %, incubated for 1 h at room temperature with secondary antibody and developed using ECL solutions.

## Conflict of interest

The Ciulli laboratory receives or has received sponsored research support from Almirall, Amgen, Amphista Therapeutics, Boehringer Ingelheim, Eisai, Nurix Therapeutics, and Ono Pharmaceutical.

1

## Supporting information

As a service to our authors and readers, this journal provides supporting information supplied by the authors. Such materials are peer reviewed and may be re‐organized for online delivery, but are not copy‐edited or typeset. Technical support issues arising from supporting information (other than missing files) should be addressed to the authors.

Supporting Information

## Data Availability

The data that support the findings of this study are available from the corresponding author upon reasonable request.
