# Supplementary material for: Discovery of Benzo[d]imidazole‐6‐sulfonamides as Bromodomain and Extra‐Terminal Domain (BET) Inhibitors with Selectivity for the First Bromodomain
Source: ChemMedChem. 2022 Sep 15;17(20):e202200343. doi: 10.1002/cmdc.202200343 (PMC9826262; doi:10.1002/cmdc.202200343)
Supplement: Supplementary file 1 — Supporting Information [file CMDC-17-0-s001.pdf]

ChemMedChem

Supporting Information

Discovery of Benzo[*d*]imidazole-6-sulfonamides as Bromodomain and Extra-Terminal Domain (BET) Inhibitors with Selectivity for the First Bromodomain

Alessandra Cipriano+, Ciro Milite+, Alessandra Feoli, Monica Viviano, Giacomo Pepe, Pietro Campiglia, Giuliana Sarno, Sarah Picaud, Satomi Imaide, Nikolai Makukhin, Panagis Filippakopoulos, Alessio Ciulli, Sabrina Castellano,* and Gianluca Sbardella

Table of Contents:

^{1}H-NMR and ^{13}C-NMR spectra of compounds **9a–p** S2–S33

HRMS spectra of compounds **9a–p** S34–S49

HPLC traces of compounds **9a–p** S50–S65

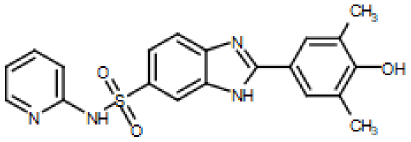

9a

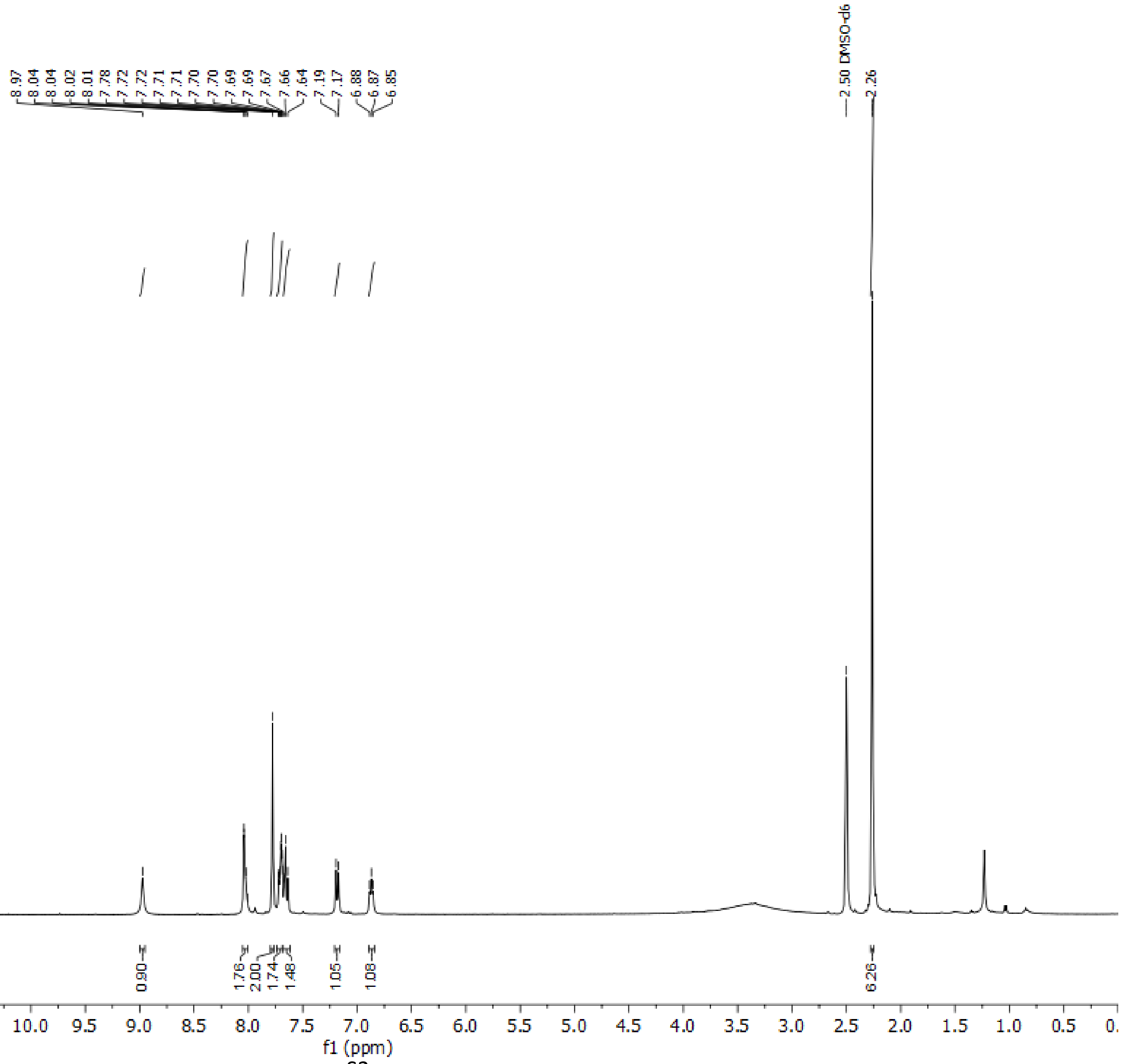

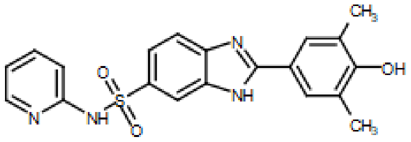
CH3
OH
CH3
N
NH
O
S
O
NH
N

9a

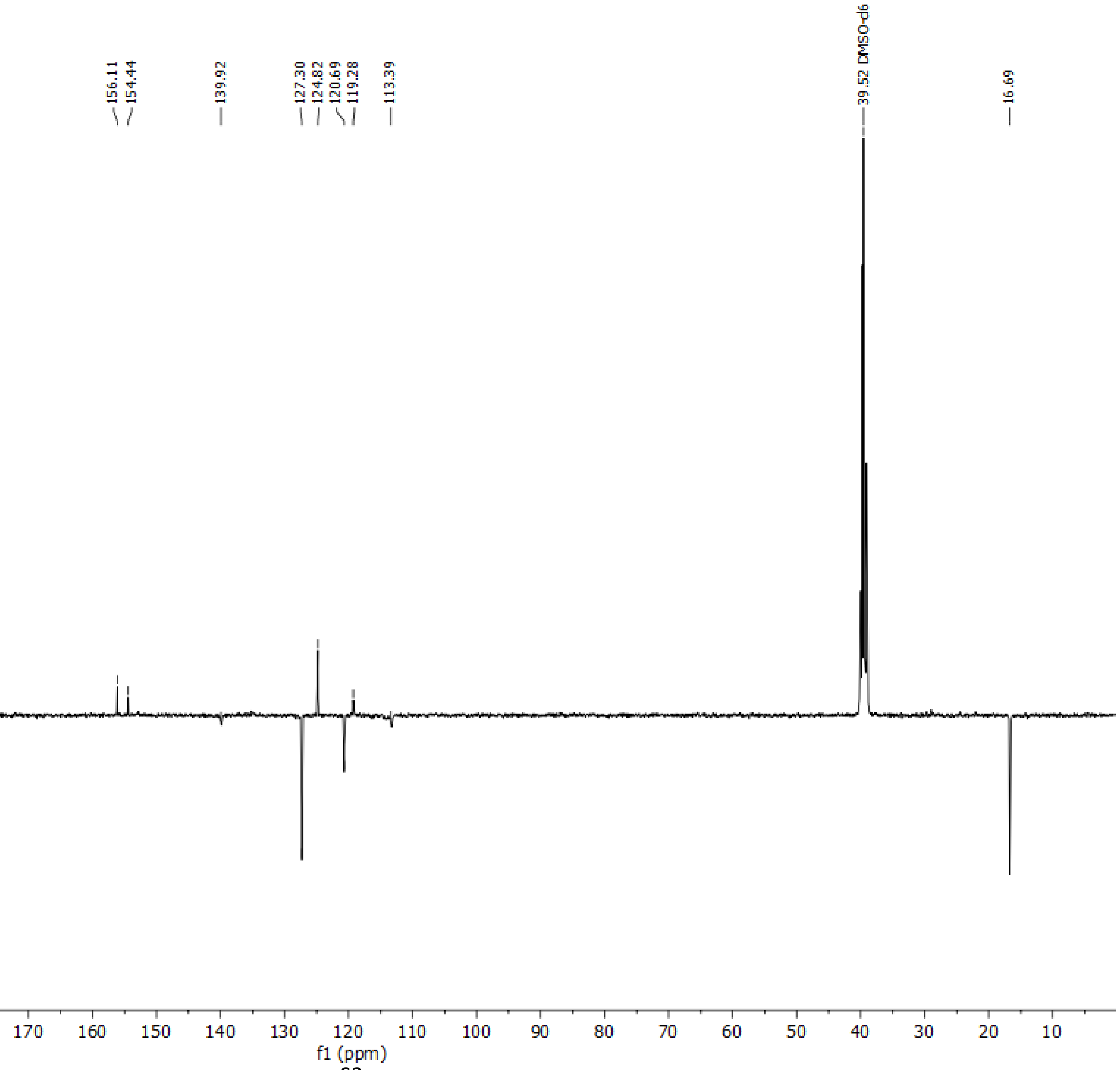
156.11
154.44
139.92
127.30
124.82
120.69
119.28
113.39
39.52 DMSO-d6
16.69
230 220 210 200 190 180 170 160 150 140 130 120 110 100 90 80 70 60 50 40 30 20 10
f1 (ppm)

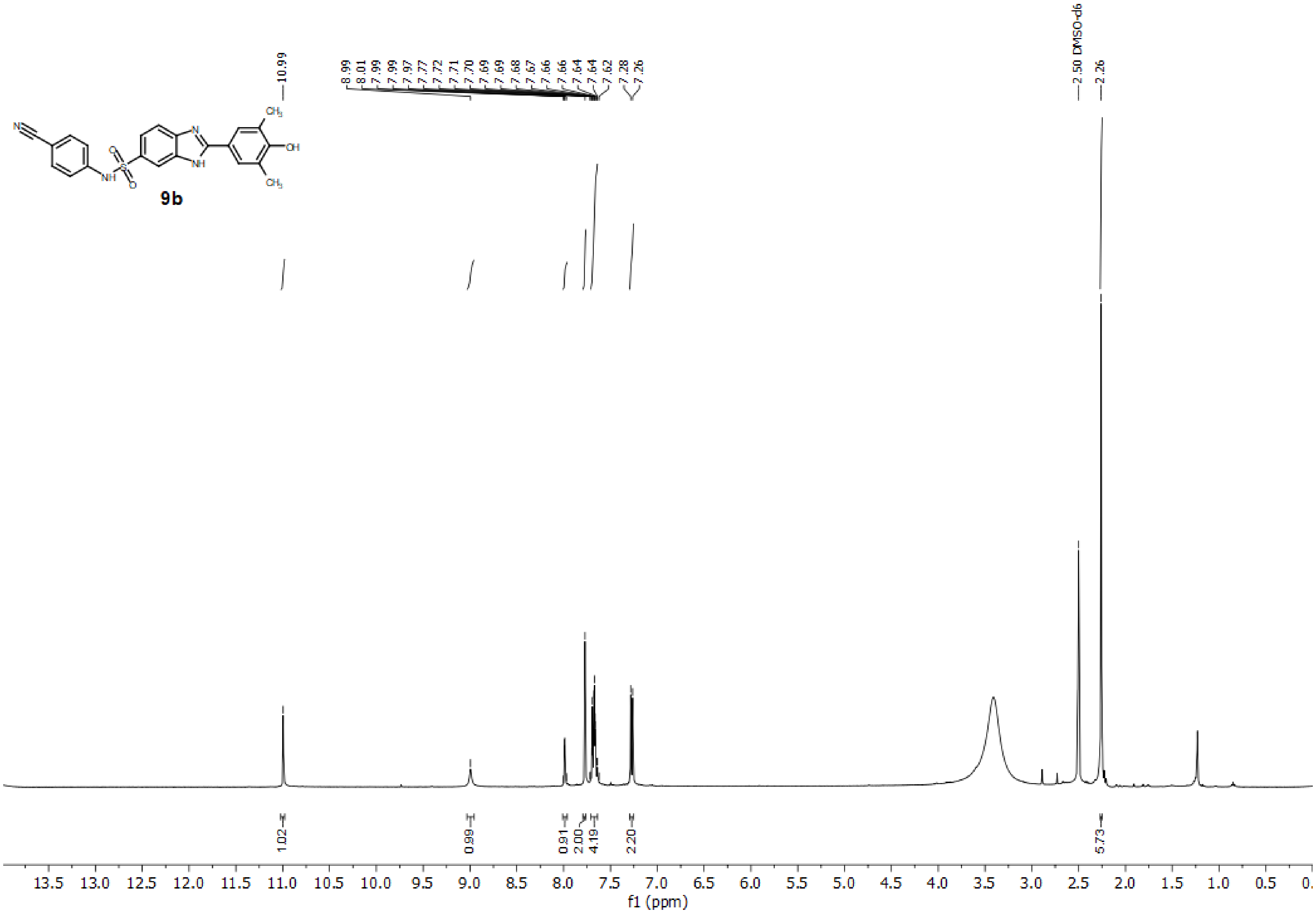
10.99
8.99
8.01
7.99
7.99
7.97
7.77
7.72
7.71
7.70
7.69
7.69
7.68
7.67
7.66
7.66
7.64
7.64
7.62
7.28
7.26
2.50 DMSO-d6
2.26
N
NH
O
S
O
NH
OH
9b
1.02
0.99
0.91
2.00
4.19
2.20
5.73
13.5 13.0 12.5 12.0 11.5 11.0 10.5 10.0 9.5 9.0 8.5 8.0 7.5 7.0 6.5 6.0 5.5 5.0 4.5 4.0 3.5 3.0 2.5 2.0 1.5 1.0 0.5
f1 (ppm)

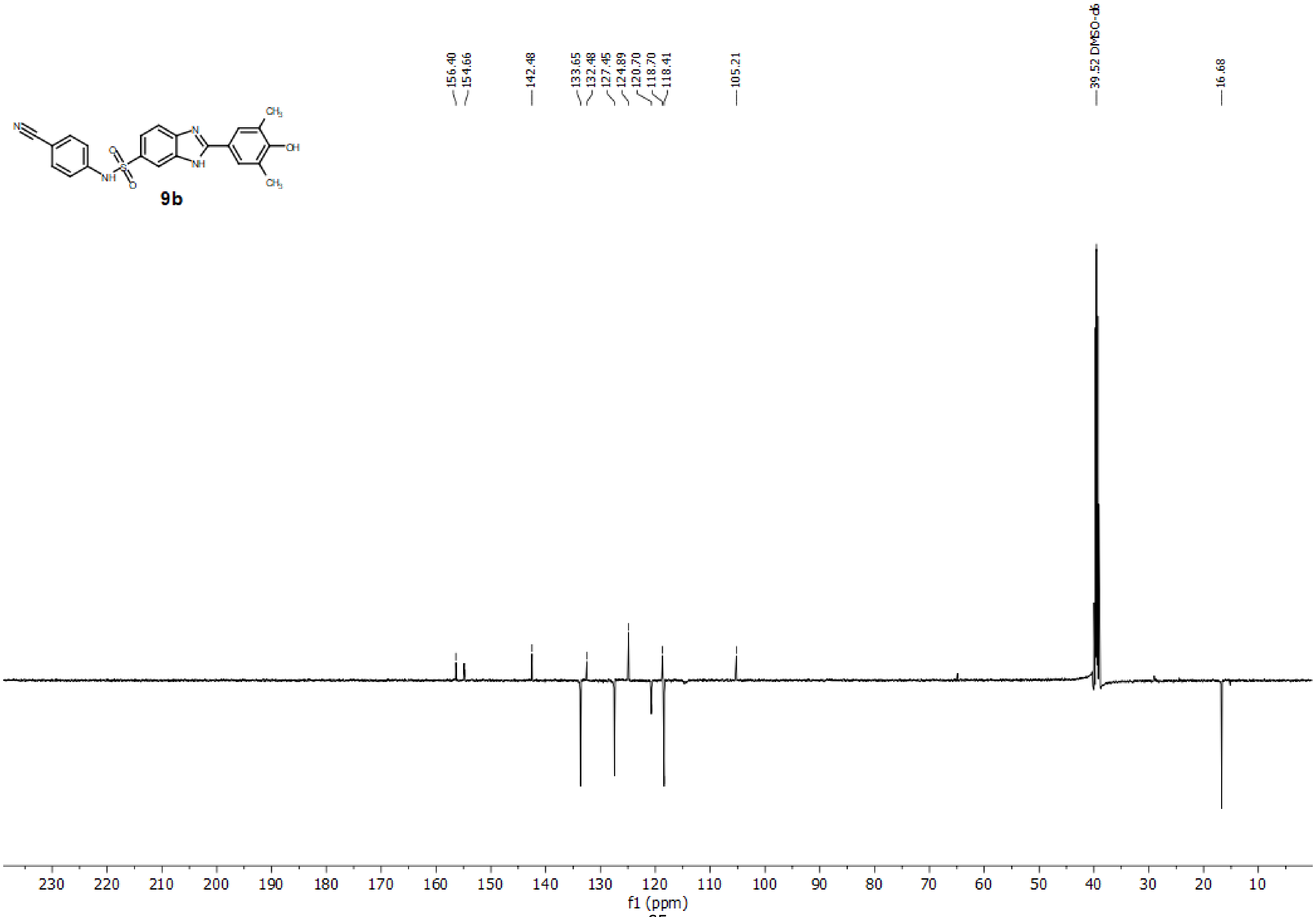
156.40
154.66
142.48
133.65
132.48
127.45
124.89
120.70
118.70
118.41
105.21
39.52 DMSO-d6
16.68
9b
230 220 210 200 190 180 170 160 150 140 130 120 110 100 90 80 70 60 50 40 30 20 10
f1 (ppm)

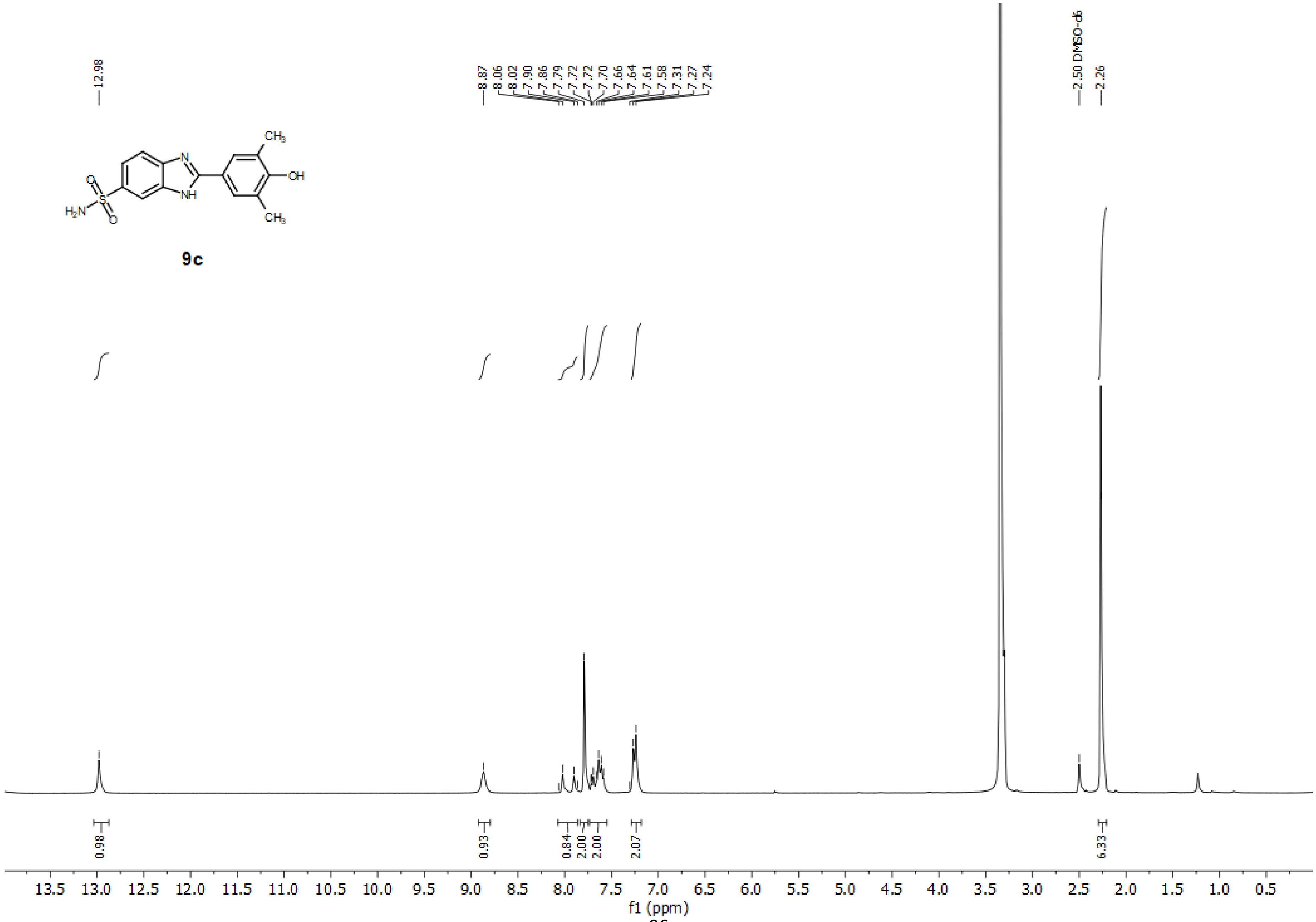

12.98
8.87
8.06
8.02
7.90
7.86
7.79
7.72
7.72
7.70
7.66
7.64
7.61
7.58
7.31
7.27
7.24
2.50 DMSO-d6
2.26
CH3
OH
CH3
N
NH
H2N
S
O
O
9c
0.98
0.93
0.84
2.00
2.00
2.07
6.33
13.5 13.0 12.5 12.0 11.5 11.0 10.5 10.0 9.5 9.0 8.5 8.0 7.5 7.0 6.5 6.0 5.5 5.0 4.5 4.0 3.5 3.0 2.5 2.0 1.5 1.0 0.5
f1 (ppm)

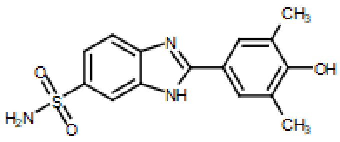

9c

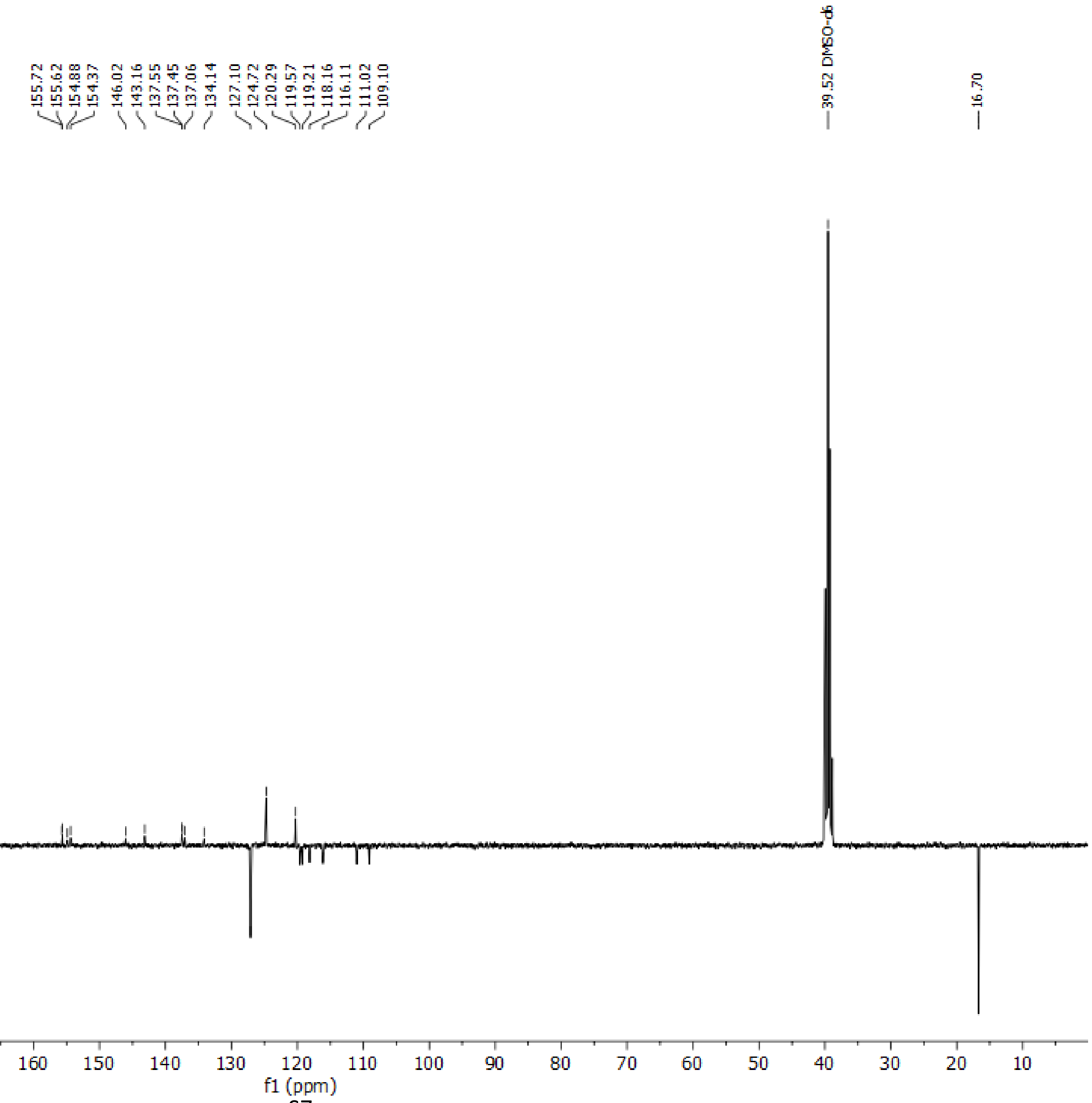
155.72
155.62
154.88
154.37
146.02
143.16
137.55
137.45
137.06
134.14
127.10
124.72
120.29
119.57
119.21
118.16
116.11
111.02
109.10
39.52 DMSO-d6
16.70
230 220 210 200 190 180 170 160 150 140 130 120 110 100 90 80 70 60 50 40 30 20 10
f1 (ppm)

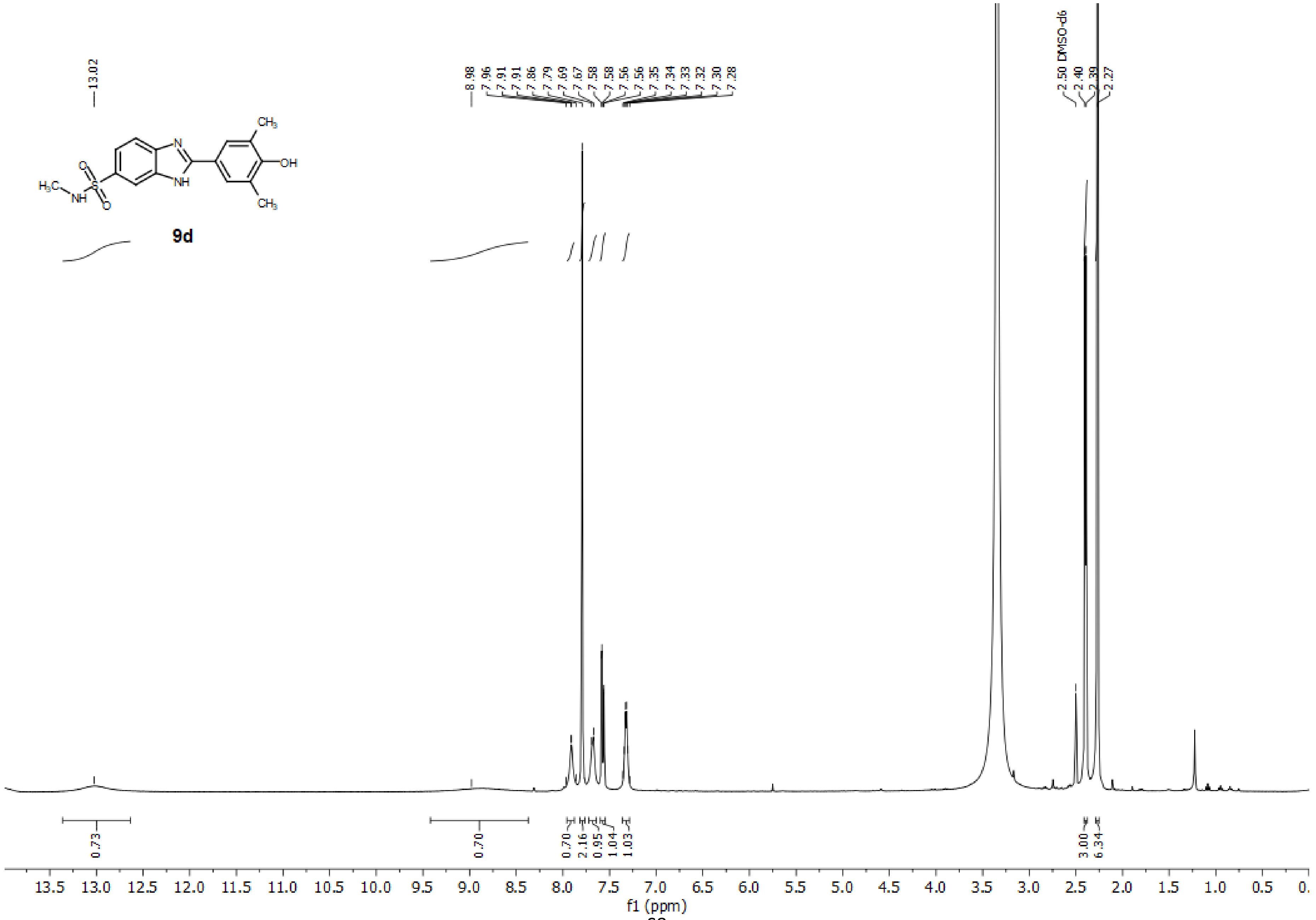

13.02
8.98 7.96 7.91 7.91 7.86 7.79 7.69 7.67 7.58 7.58 7.56 7.56 7.35 7.34 7.33 7.32 7.30 7.28
2.50 DMSO-d6 2.40 2.39 2.27
0.73 0.70 0.70 2.16 0.95 1.04 1.03 3.00 6.34
f1 (ppm)

9d

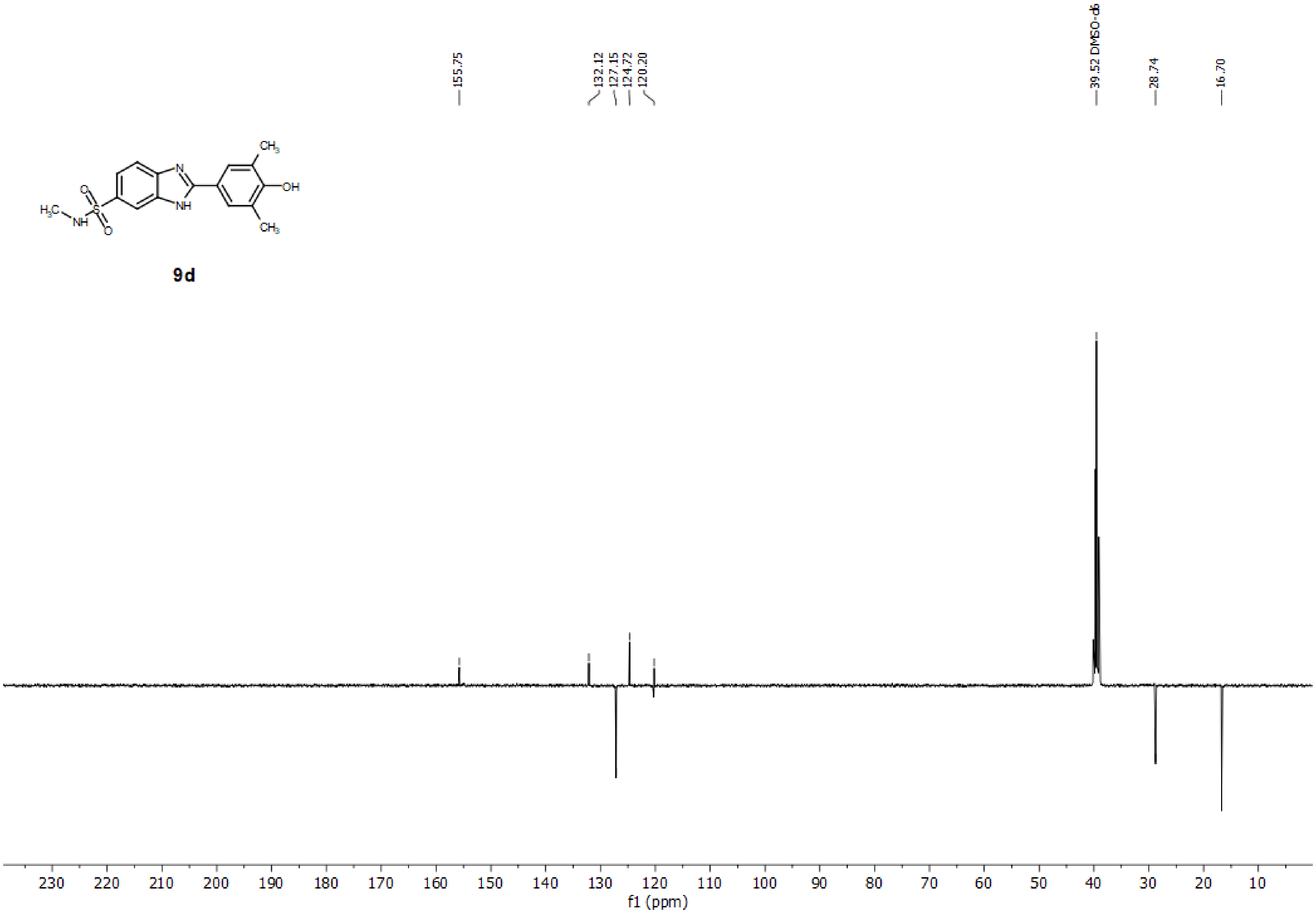
155.75
132.12
127.15
124.72
120.20
39.52 DMSO-d6
28.74
16.70
H3C
NH
O
S
O
N
NH
CH3
OH
CH3
9d
230 220 210 200 190 180 170 160 150 140 130 120 110 100 90 80 70 60 50 40 30 20 10
f1 (ppm)

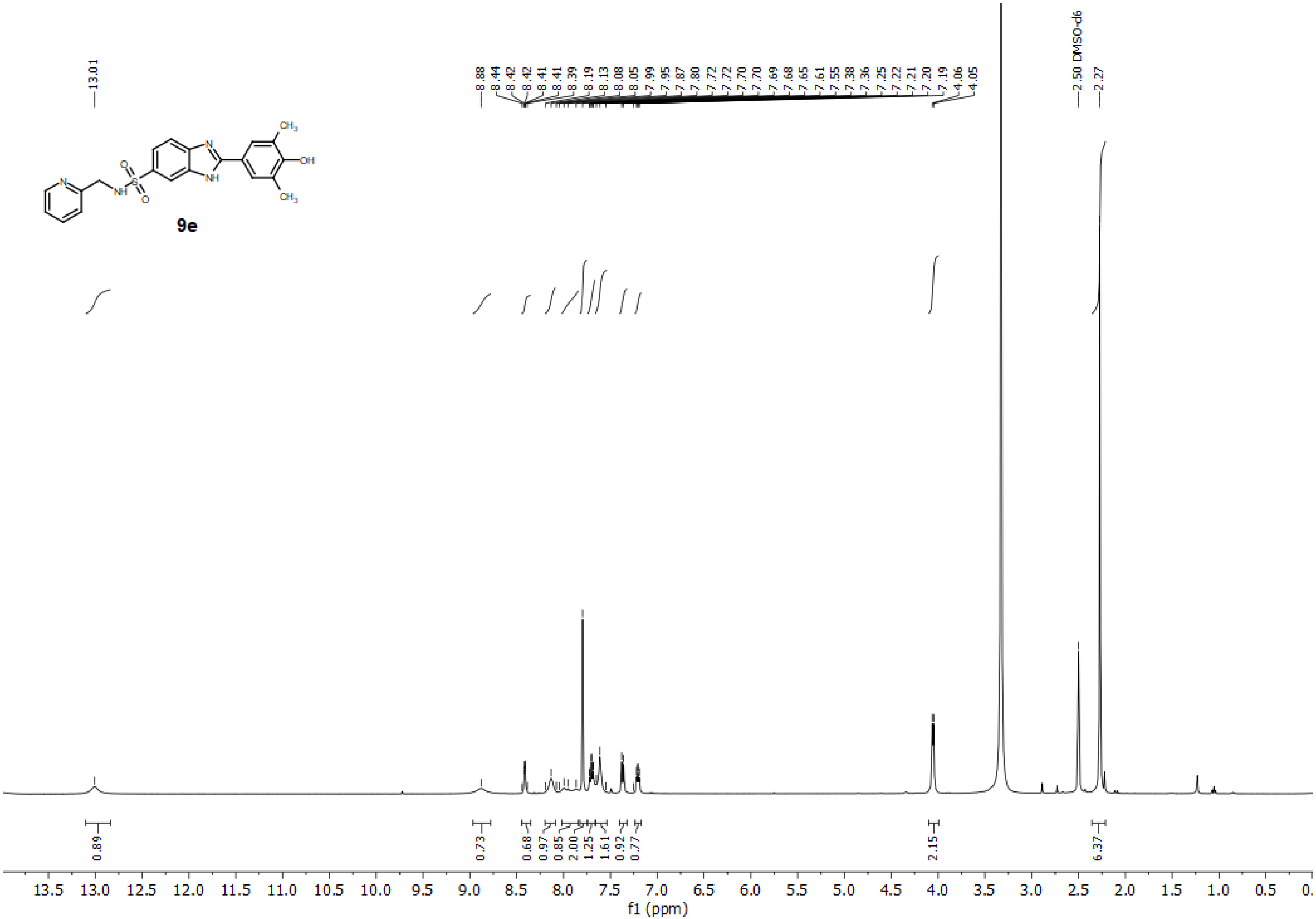
13.01
8.88 8.44 8.42 8.42 8.41 8.41 8.39 8.19 8.13 8.08 8.05 7.99 7.95 7.87 7.80 7.72 7.72 7.70 7.70 7.69 7.68 7.65 7.61 7.55 7.38 7.36 7.25 7.22 7.21 7.20 7.19 4.06 4.05
2.50 DMSO-d6
2.27
9e
0.89 0.73 0.68 0.97 0.85 2.00 1.25 1.61 0.92 0.77 2.15 6.37
13.5 13.0 12.5 12.0 11.5 11.0 10.5 10.0 9.5 9.0 8.5 8.0 7.5 7.0 6.5 6.0 5.5 5.0 4.5 4.0 3.5 3.0 2.5 2.0 1.5 1.0 0.5 0.
f1 (ppm)

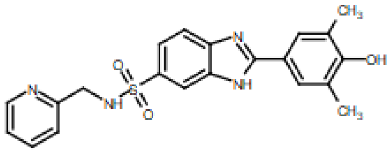

9e

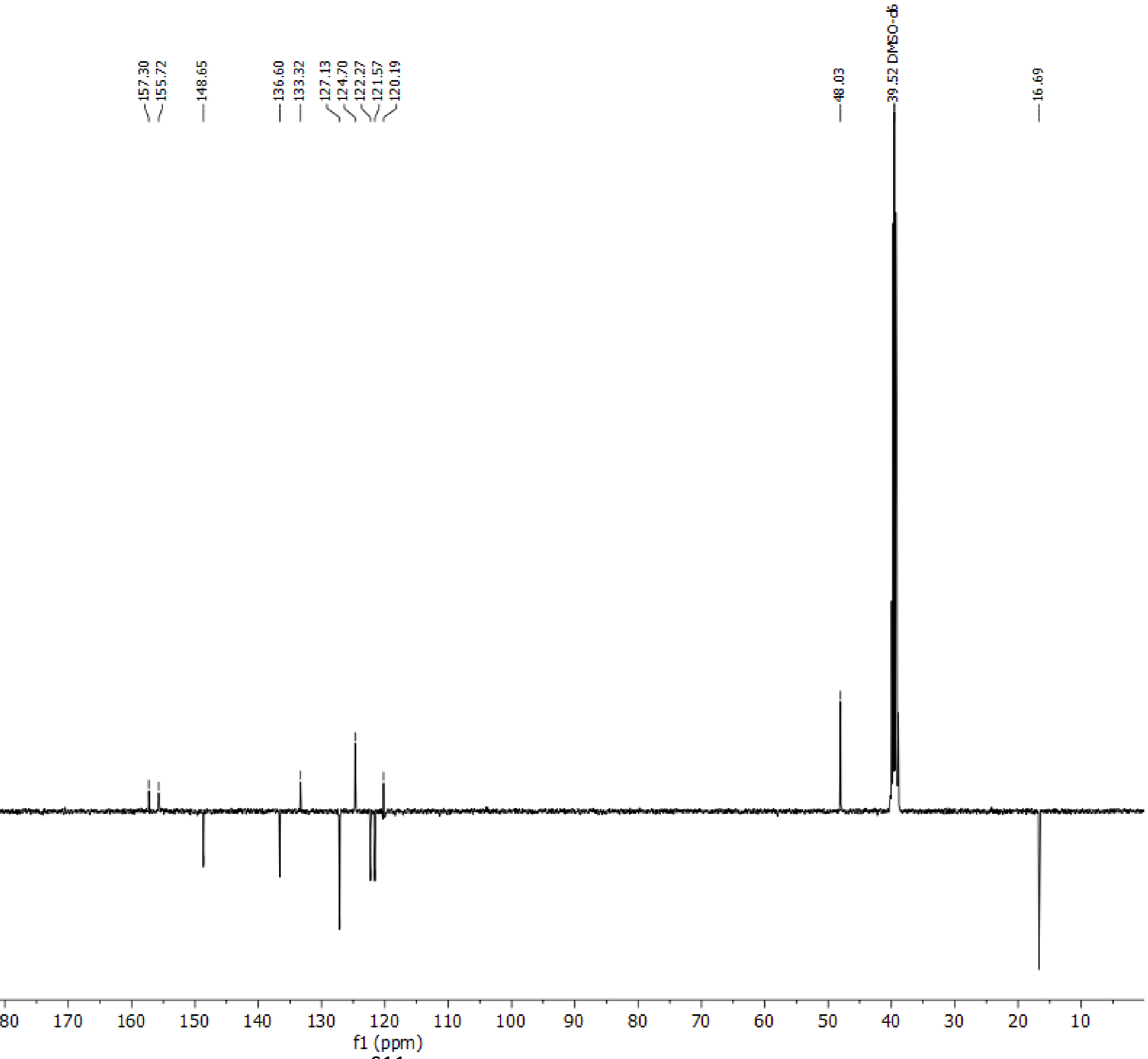

157.30
155.72
148.65
136.60
133.32
127.13
124.70
122.27
121.57
120.19
48.03
39.52 DMSO-d6
16.69
230 220 210 200 190 180 170 160 150 140 130 120 110 100 90 80 70 60 50 40 30 20 10
f1 (ppm)

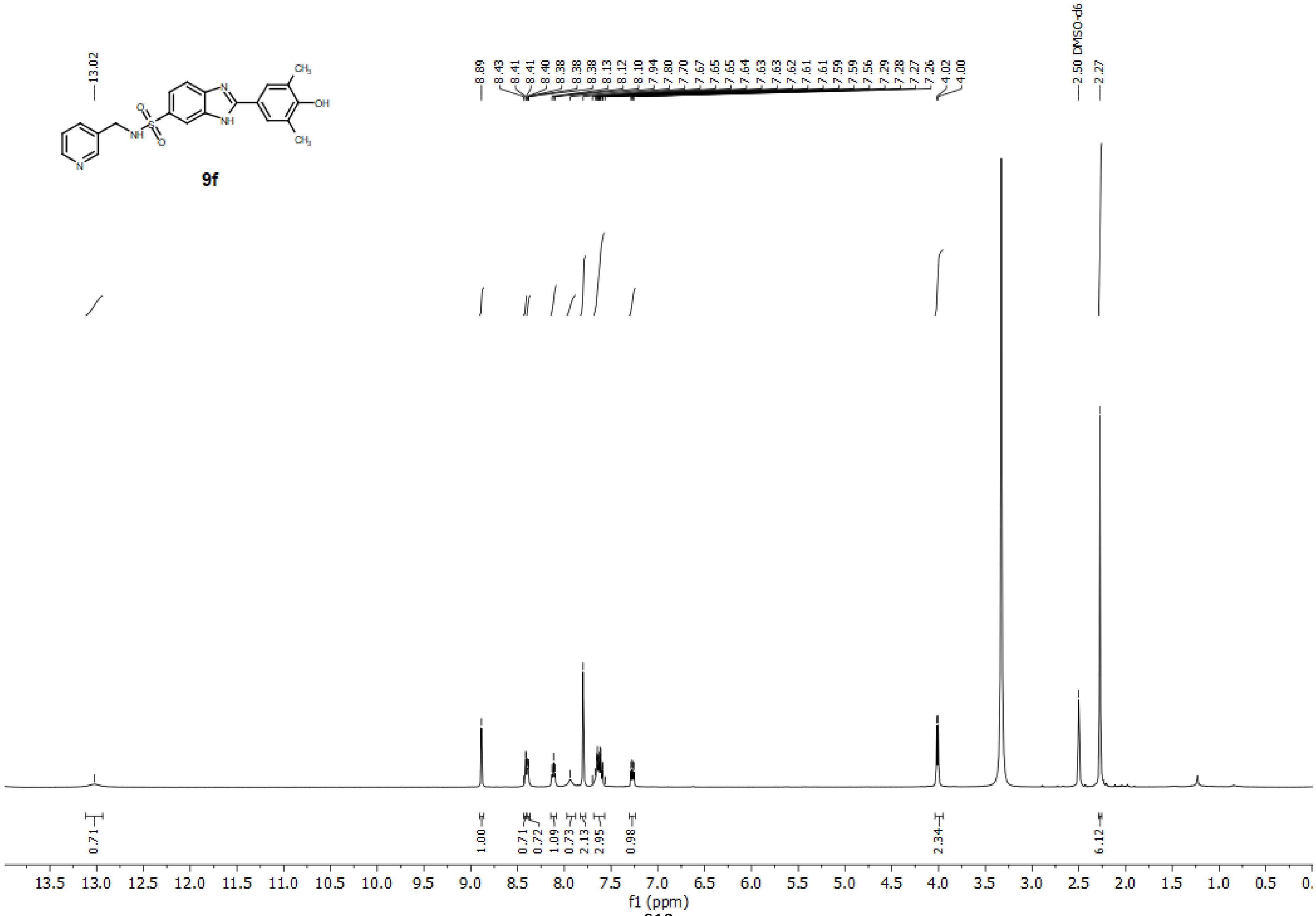

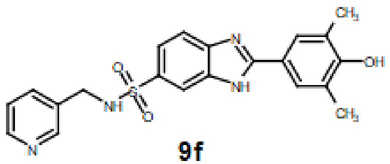

9f

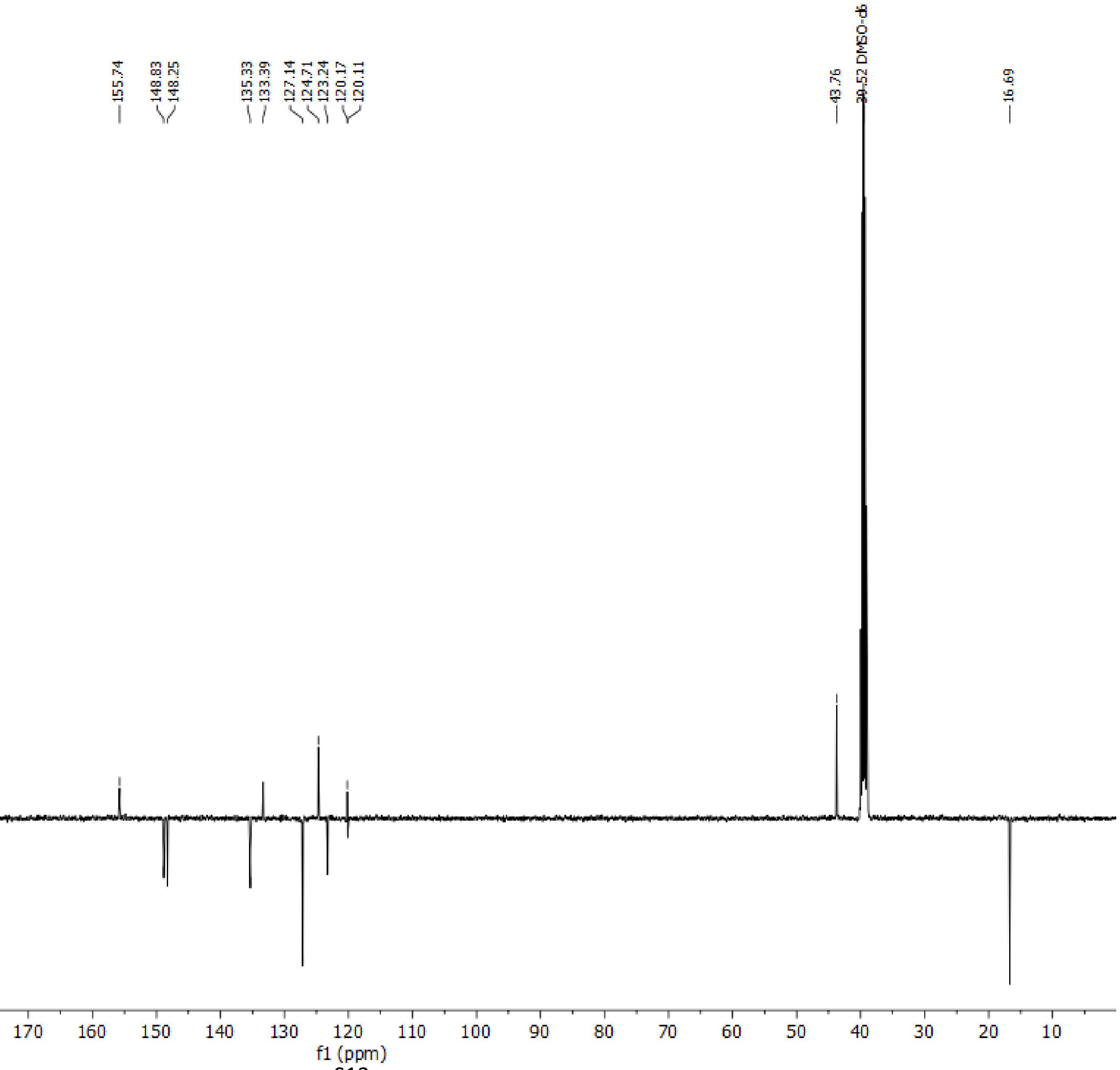
155.74
148.83
148.25
135.33
133.39
127.14
124.71
123.24
120.17
120.11
43.76
39.52 DMSO-d6
16.69
230
220
210
200
190
180
170
160
150
140
130
120
110
100
90
80
70
60
50
40
30
20
10
f1 (ppm)

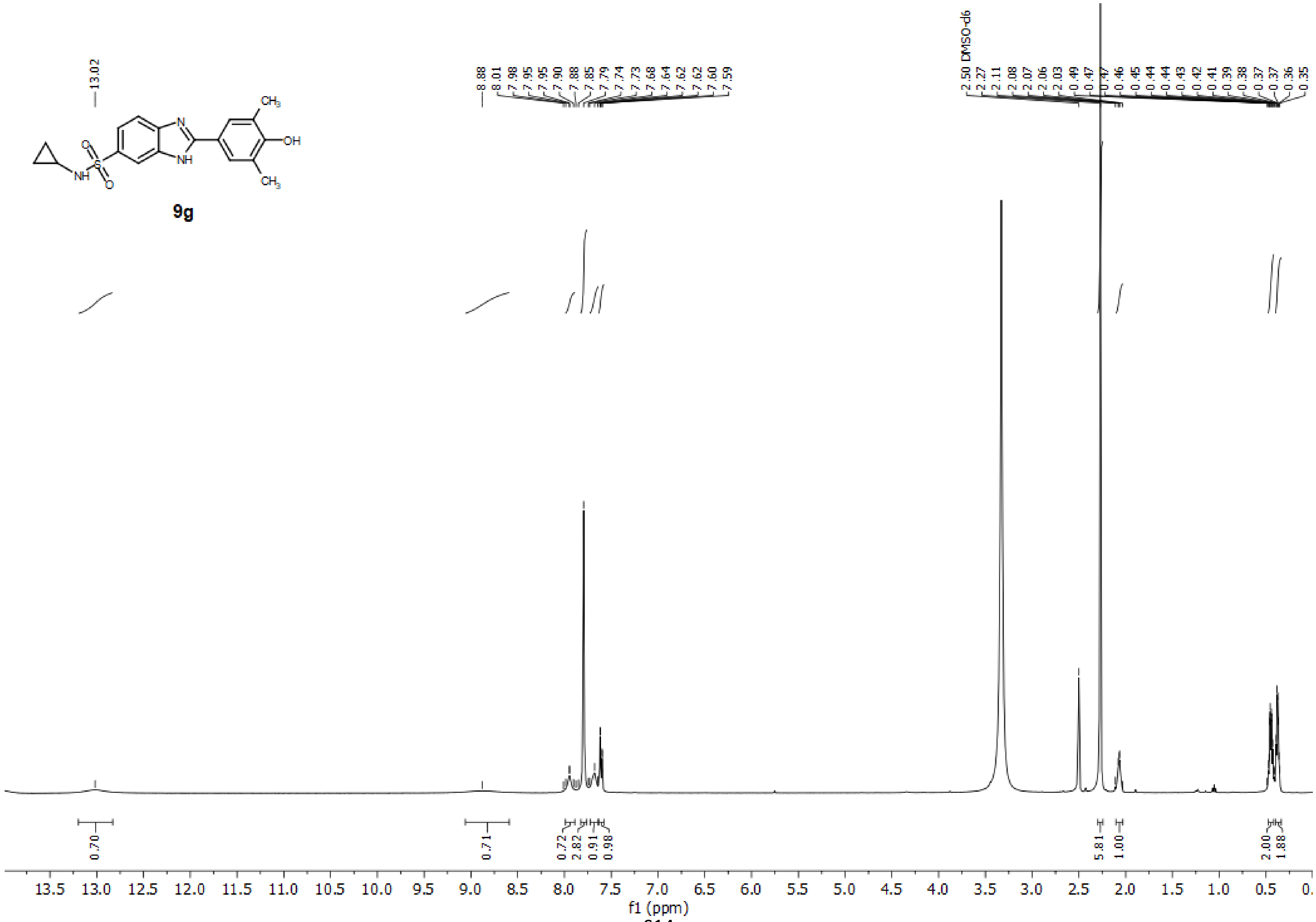
9g
13.02
8.88
8.01
7.98
7.95
7.95
7.90
7.88
7.85
7.79
7.74
7.73
7.68
7.64
7.62
7.62
7.60
7.59
2.50 DMSO-d6
2.27
2.11
2.08
2.07
2.06
2.03
0.49
0.47
0.47
0.46
0.45
0.44
0.44
0.43
0.42
0.41
0.39
0.38
0.37
0.37
0.36
0.35
0.70
0.71
0.72
2.82
0.91
0.98
5.81
1.00
2.00
1.88
f1 (ppm)

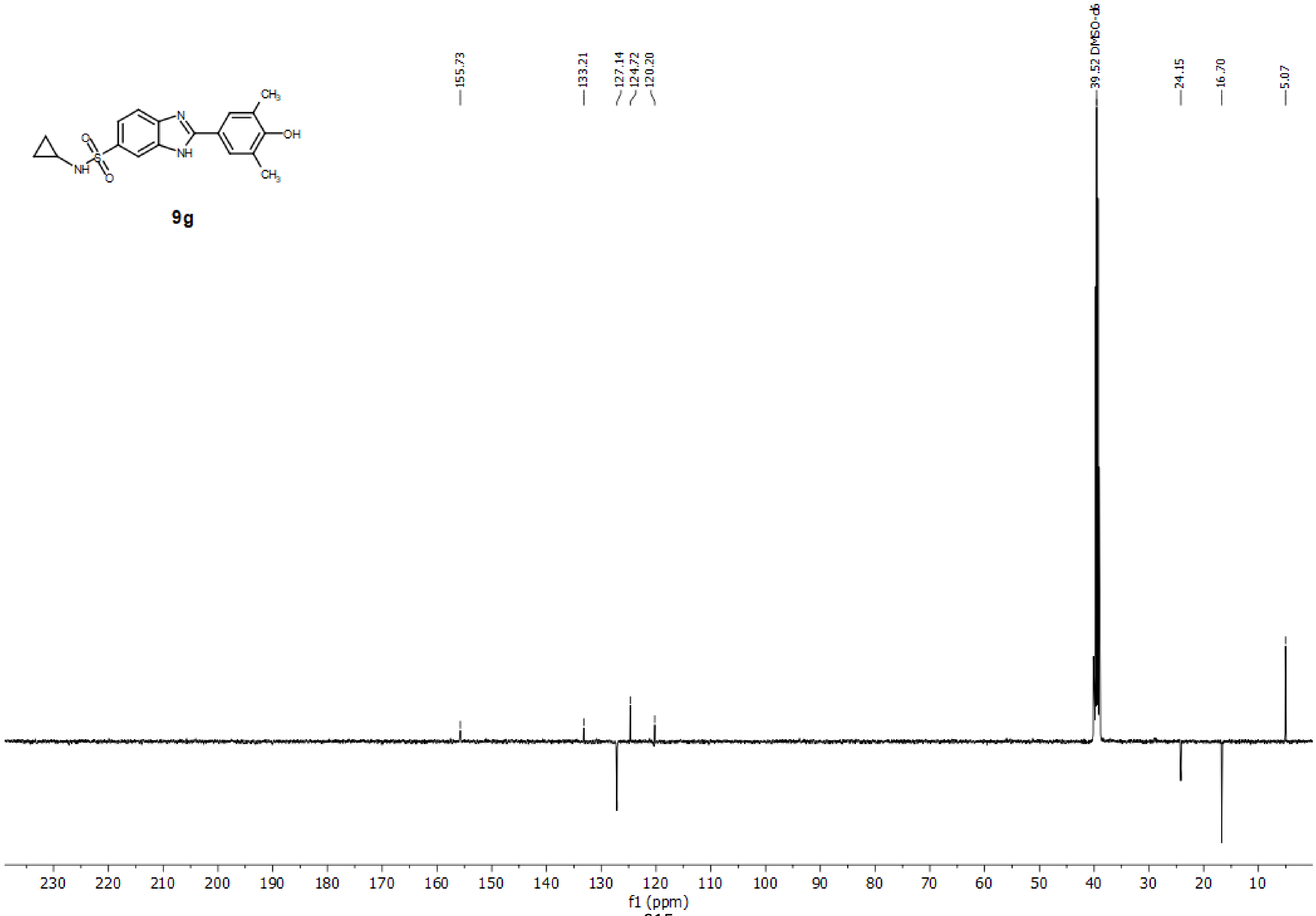

9g
155.73
133.21
127.14
124.72
120.20
39.52 DMSO-d6
24.15
16.70
5.07
230 220 210 200 190 180 170 160 150 140 130 120 110 100 90 80 70 60 50 40 30 20 10
f1 (ppm)

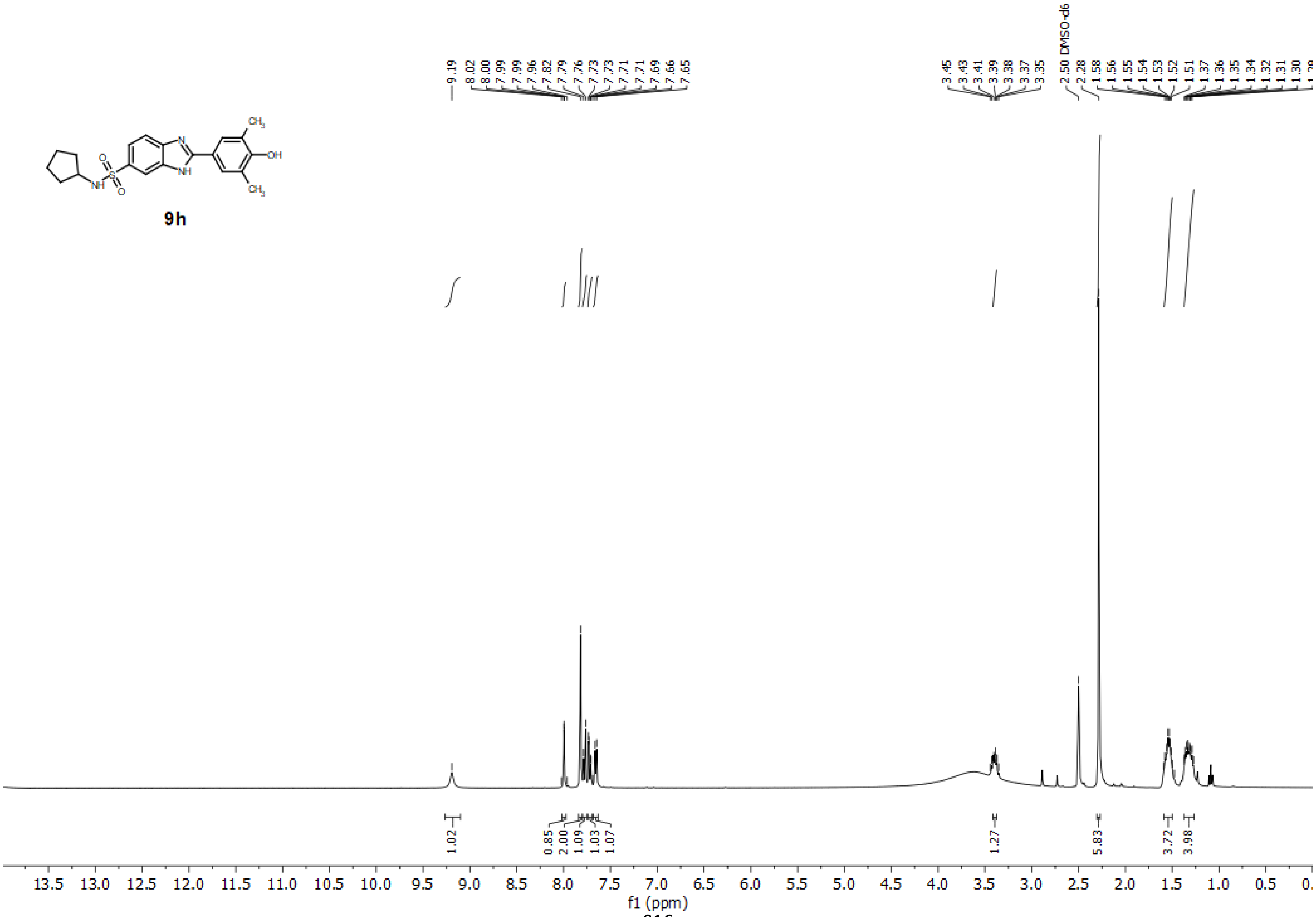
9h
f1 (ppm)

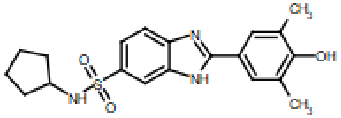
CH3
OH
CH3
NH
N
NH
O
S
O

9h

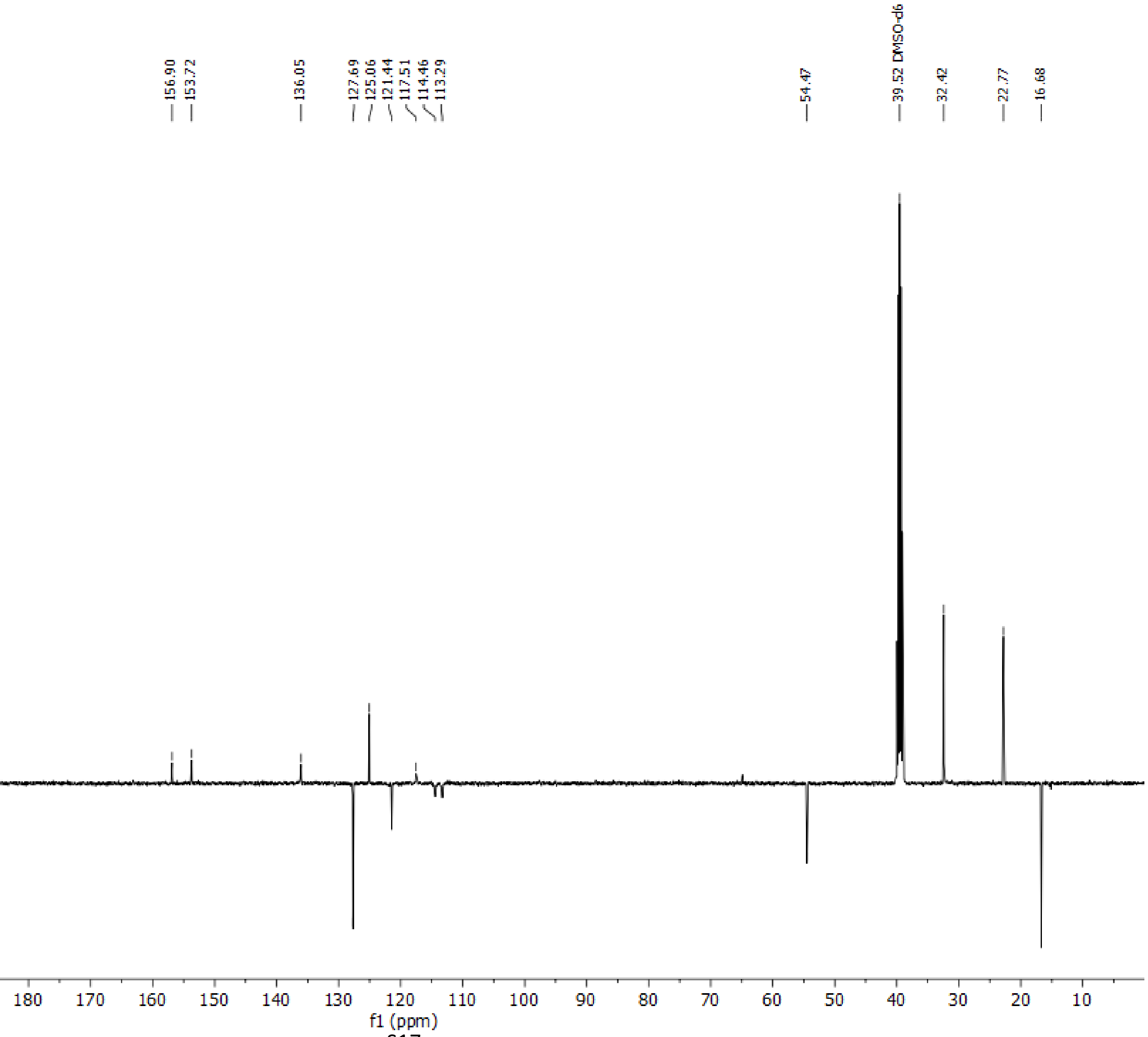
156.90
153.72
136.05
127.69
125.06
121.44
117.51
114.46
113.29
54.47
39.52 DMSO-d6
32.42
22.77
16.68
230
220
210
200
190
180
170
160
150
140
130
120
110
100
90
80
70
60
50
40
30
20
10
f1 (ppm)

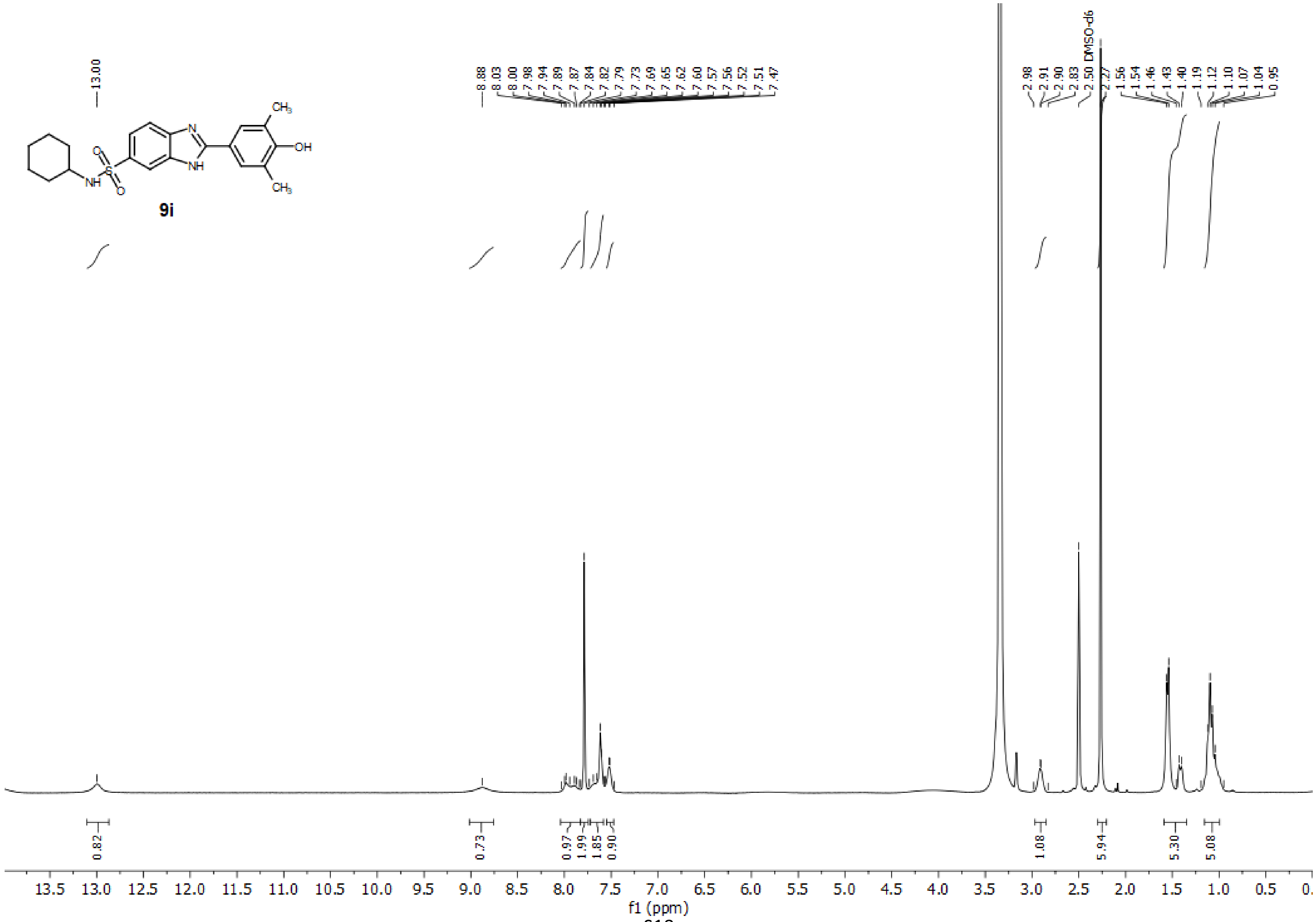

S18

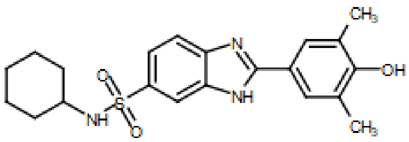

9i

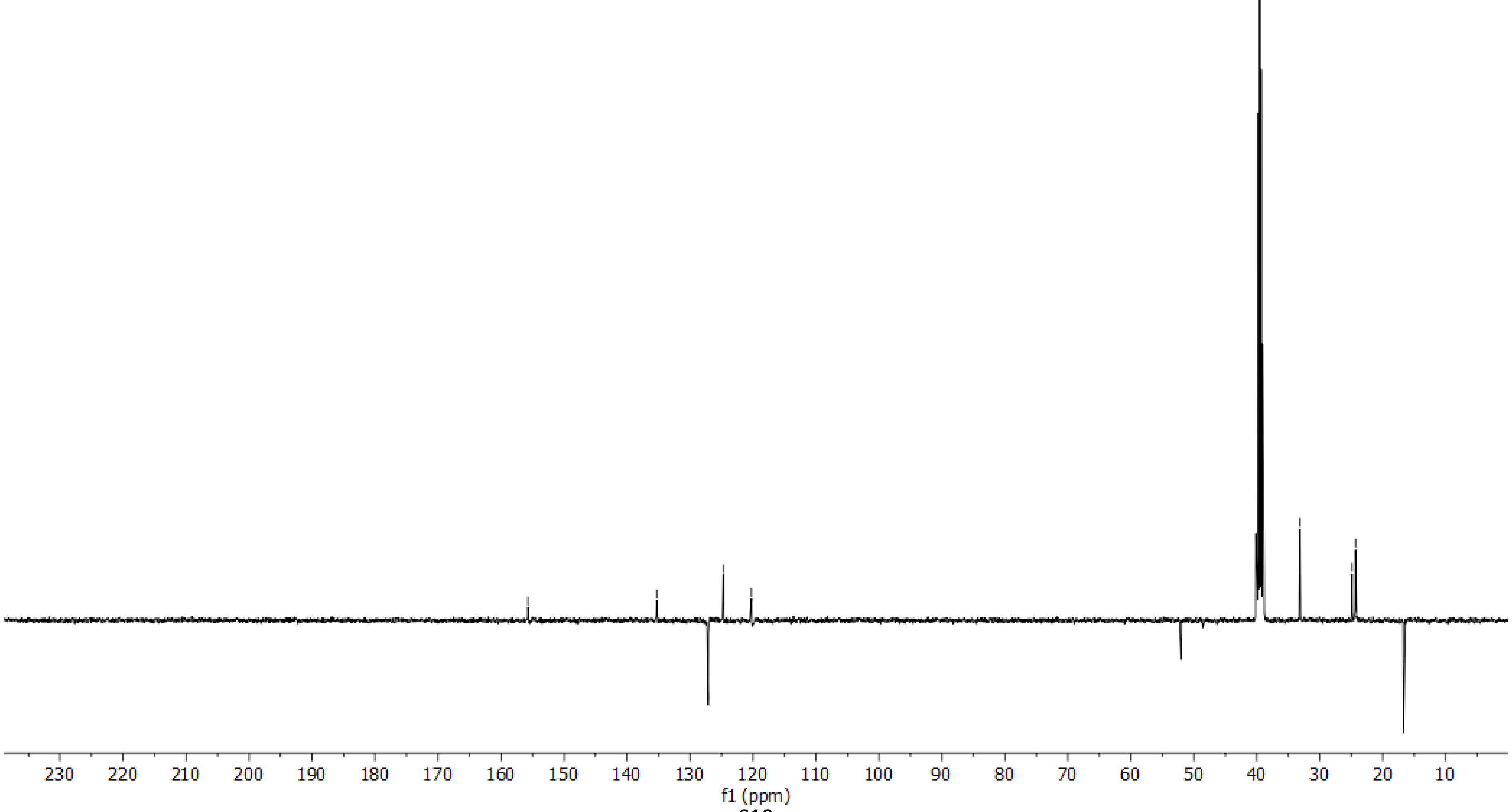
155.71
135.27
127.12
124.71
120.23
52.01
39.52 DMSO-d6
33.18
24.87
24.33
16.70
230 220 210 200 190 180 170 160 150 140 130 120 110 100 90 80 70 60 50 40 30 20 10
f1 (ppm)

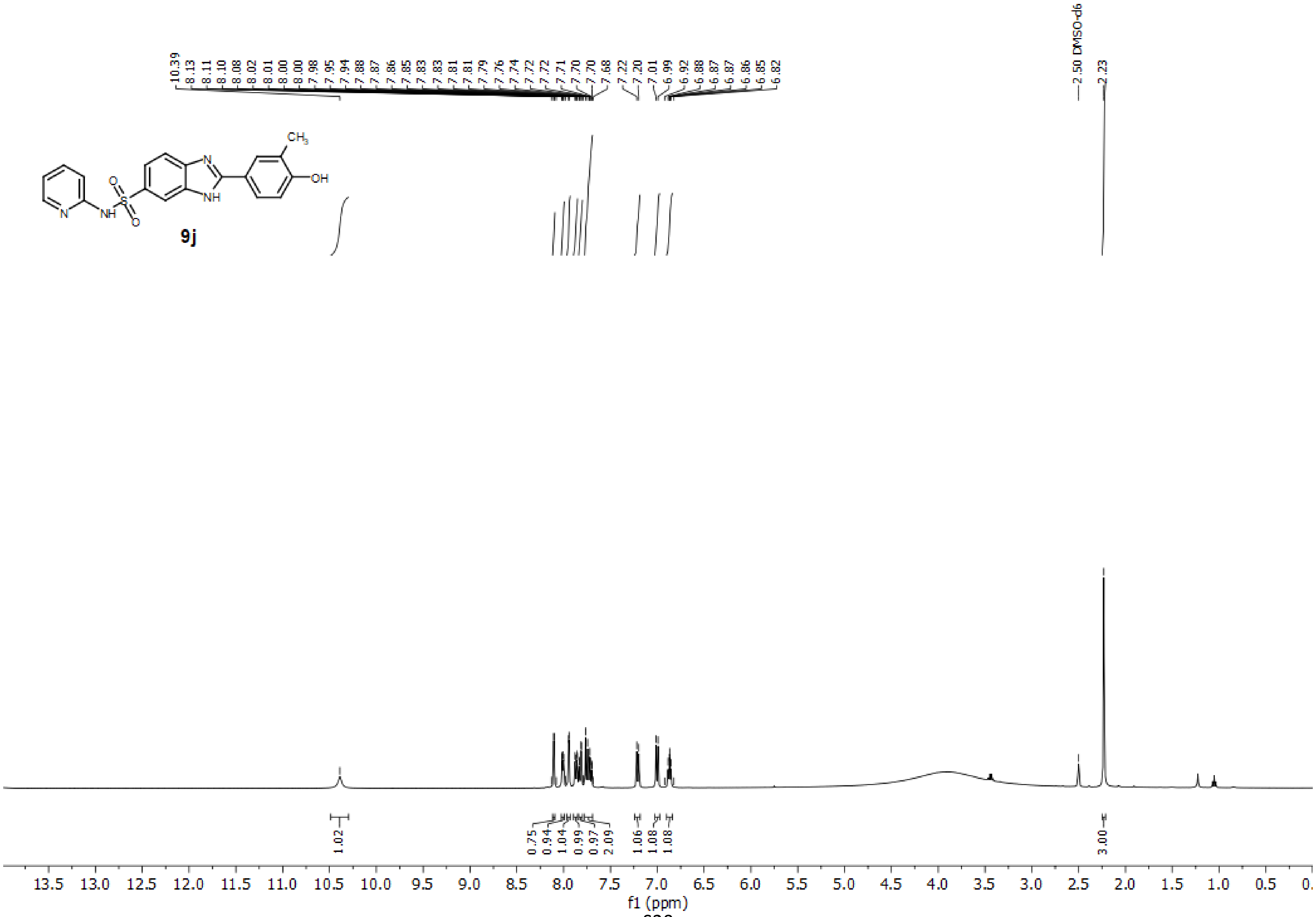
10.39
8.13
8.11
8.10
8.08
8.02
8.01
8.00
8.00
7.98
7.95
7.94
7.88
7.87
7.86
7.85
7.83
7.83
7.81
7.81
7.79
7.76
7.74
7.72
7.72
7.71
7.70
7.70
7.68
7.22
7.20
7.01
6.99
6.92
6.88
6.87
6.87
6.86
6.85
6.82
2.50 DMSO-d6
2.23
CH3
N
NH
O
S
OH
9j
1.02
0.75
0.94
1.04
0.99
0.97
2.09
1.06
1.08
1.08
3.00
13.5 13.0 12.5 12.0 11.5 11.0 10.5 10.0 9.5 9.0 8.5 8.0 7.5 7.0 6.5 6.0 5.5 5.0 4.5 4.0 3.5 3.0 2.5 2.0 1.5 1.0 0.5 0.
f1 (ppm)

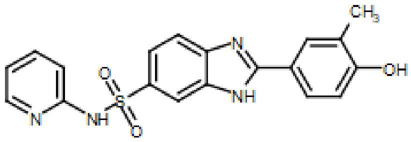
CH3
OH
N
NH
O
S
O
NH
N

9j

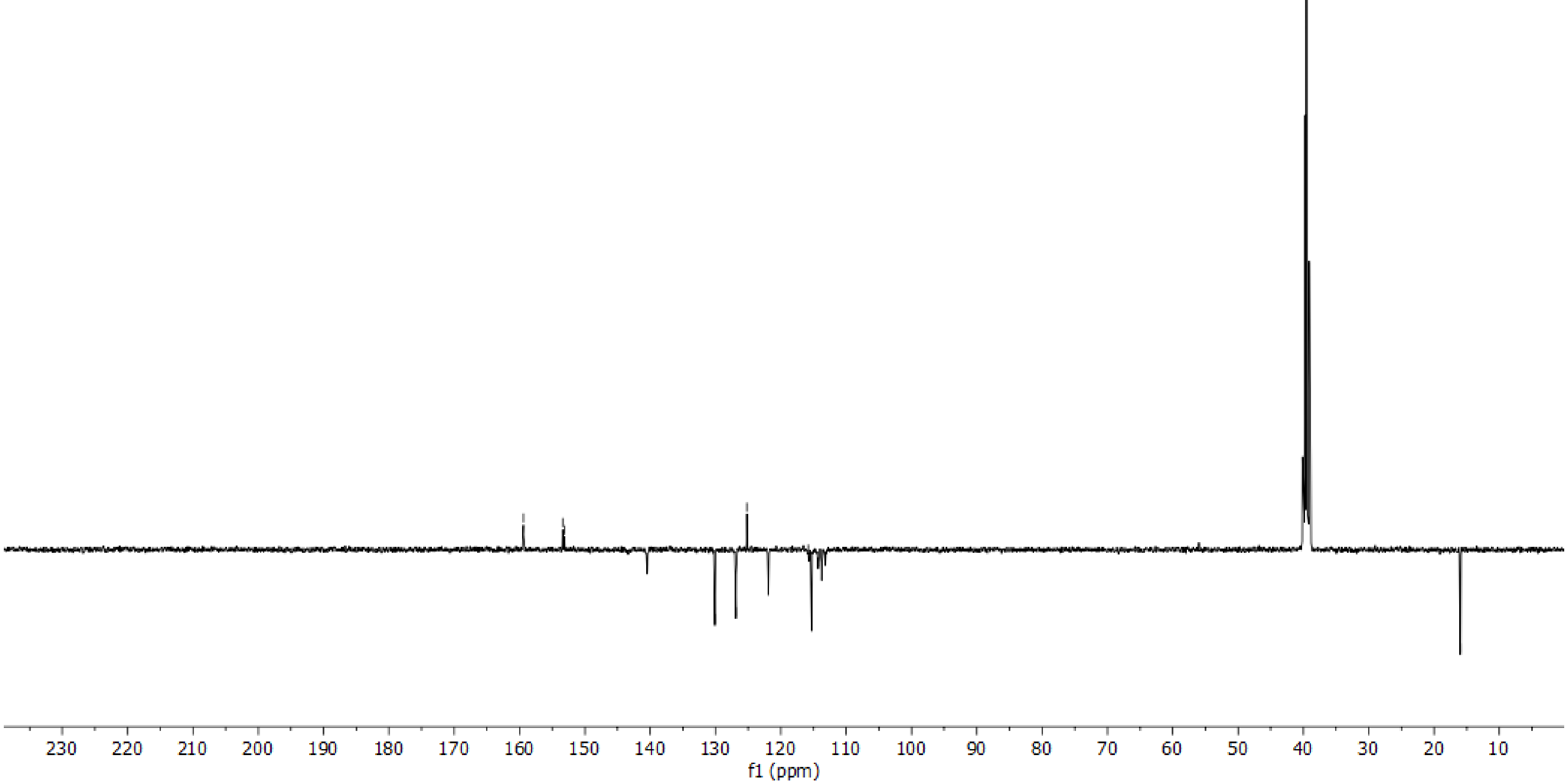
159.42
153.40
153.11
140.46
130.07
126.88
125.22
121.91
115.82
115.67
114.30
114.27
113.23
39.52 DMSO-d6
15.97
230 220 210 200 190 180 170 160 150 140 130 120 110 100 90 80 70 60 50 40 30 20 10
f1 (ppm)

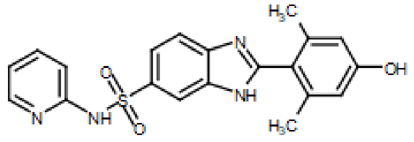
H3C
OH
N
NH
O
S
O
NH
N
H3C

9k

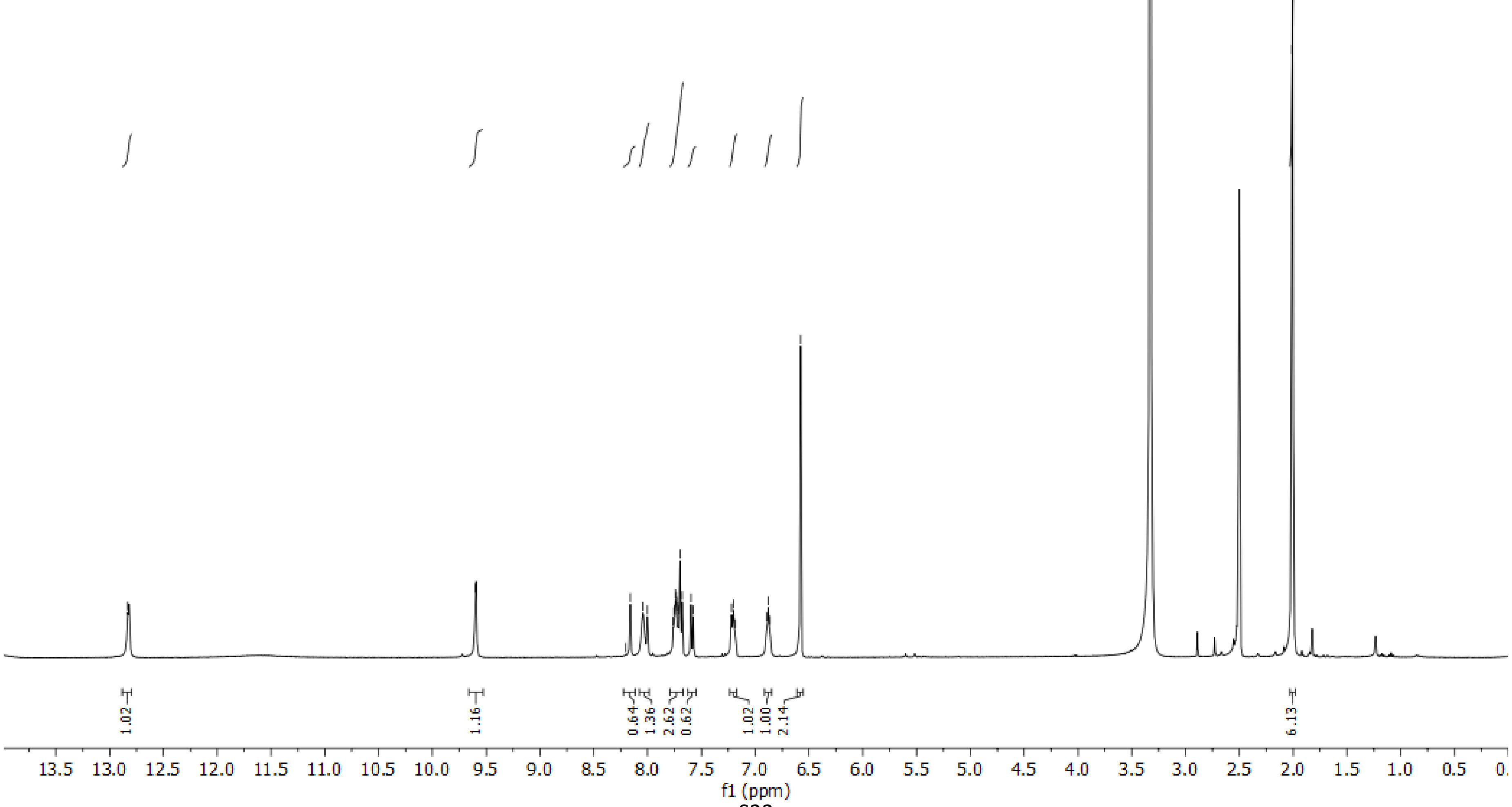
12.83
9.60
8.20
8.16
8.04
8.00
7.76
7.75
7.75
7.74
7.73
7.73
7.72
7.71
7.70
7.68
7.60
7.58
7.22
7.20
7.18
6.89
6.88
6.86
6.58
2.01
1.02
1.16
0.64
1.36
2.62
0.62
1.02
1.00
2.14
6.13
13.5 13.0 12.5 12.0 11.5 11.0 10.5 10.0 9.5 9.0 8.5 8.0 7.5 7.0 6.5 6.0 5.5 5.0 4.5 4.0 3.5 3.0 2.5 2.0 1.5 1.0 0.5 0.
f1 (ppm)

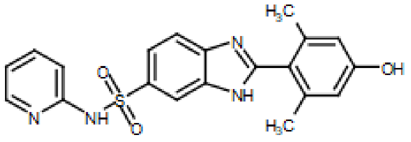

9k

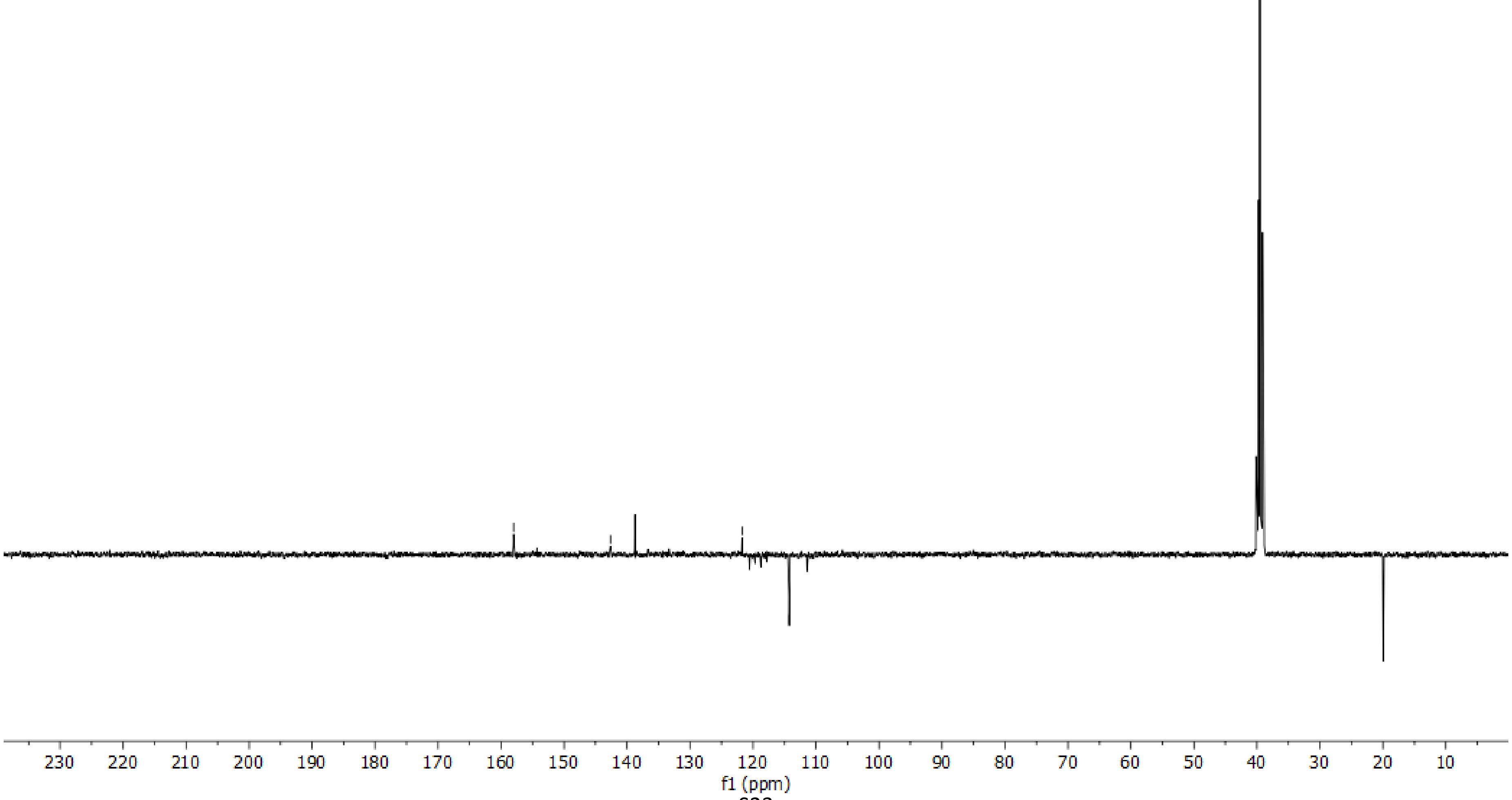
158.00
142.62
121.73
120.55
119.65
118.75
117.79
114.23
111.41
19.93
230 220 210 200 190 180 170 160 150 140 130 120 110 100 90 80 70 60 50 40 30 20 10
f1 (ppm)

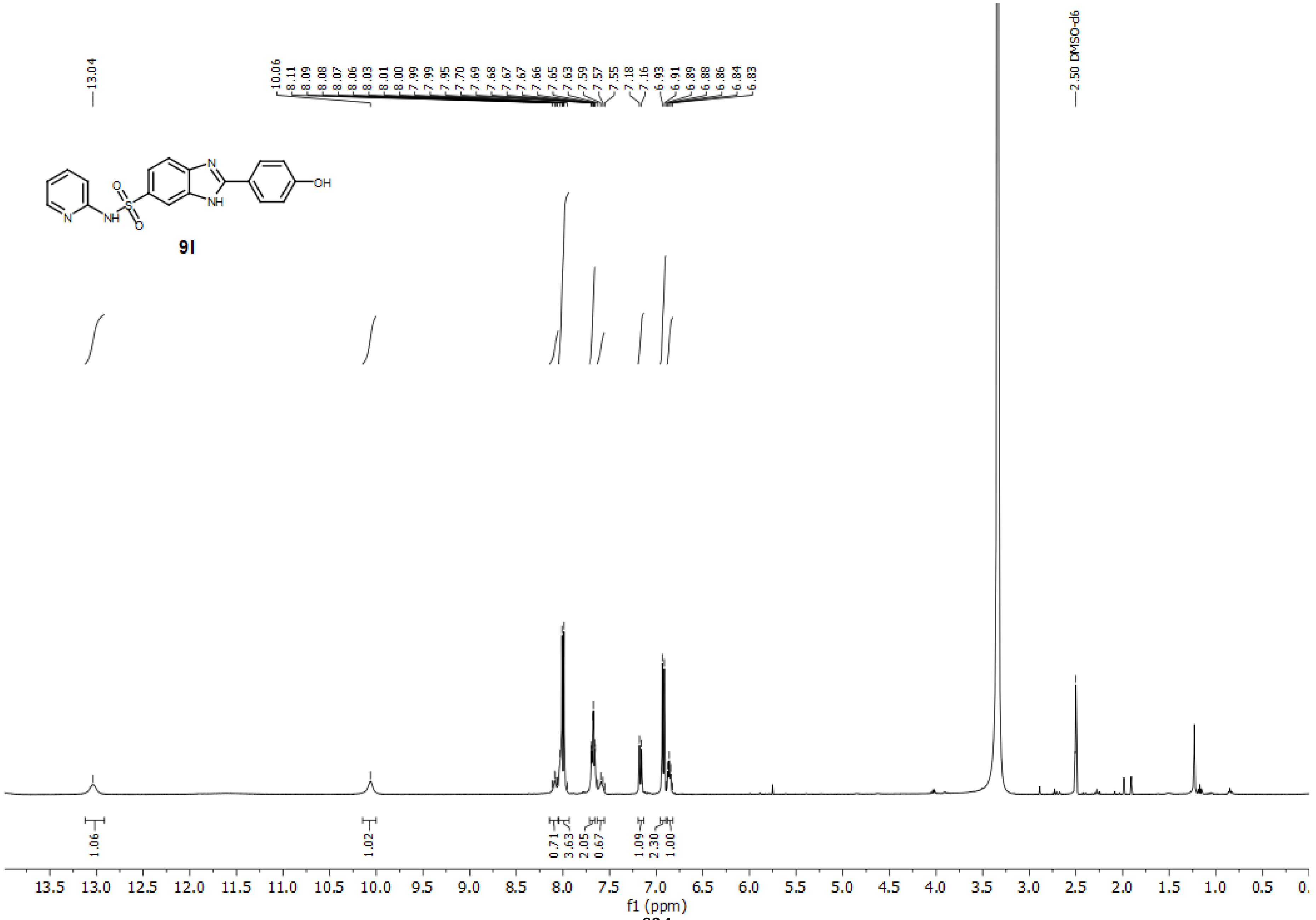

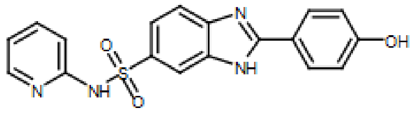

9l

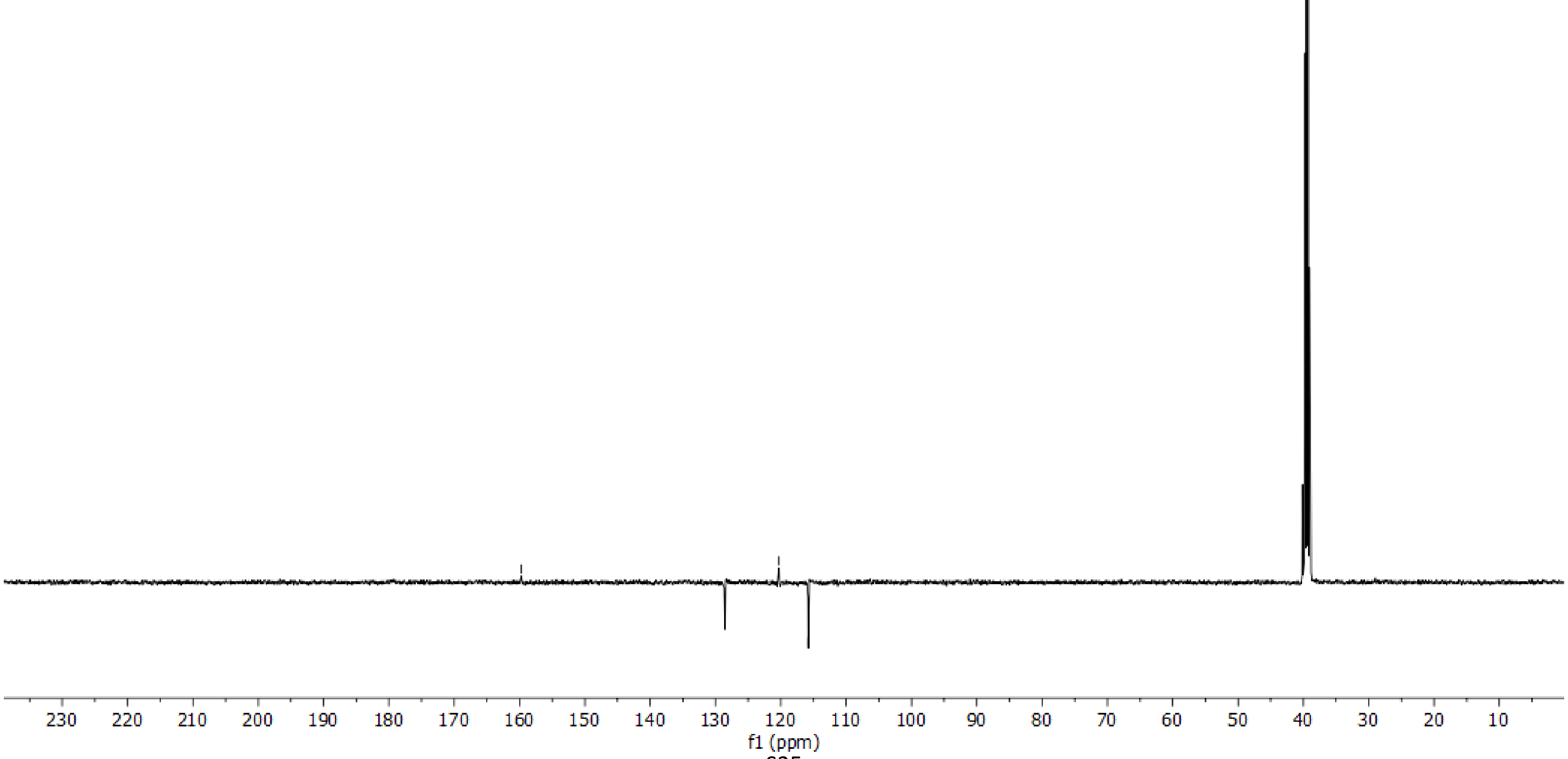

159.73
128.55
120.27
115.80
39.52 DMSO-d6
230
220
210
200
190
180
170
160
150
140
130
120
110
100
90
80
70
60
50
40
30
20
10
f1 (ppm)

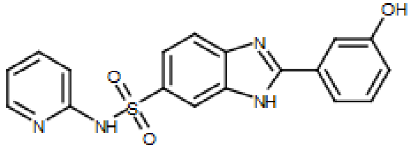

OH
N
NH
O
S
O
NH
N

9m

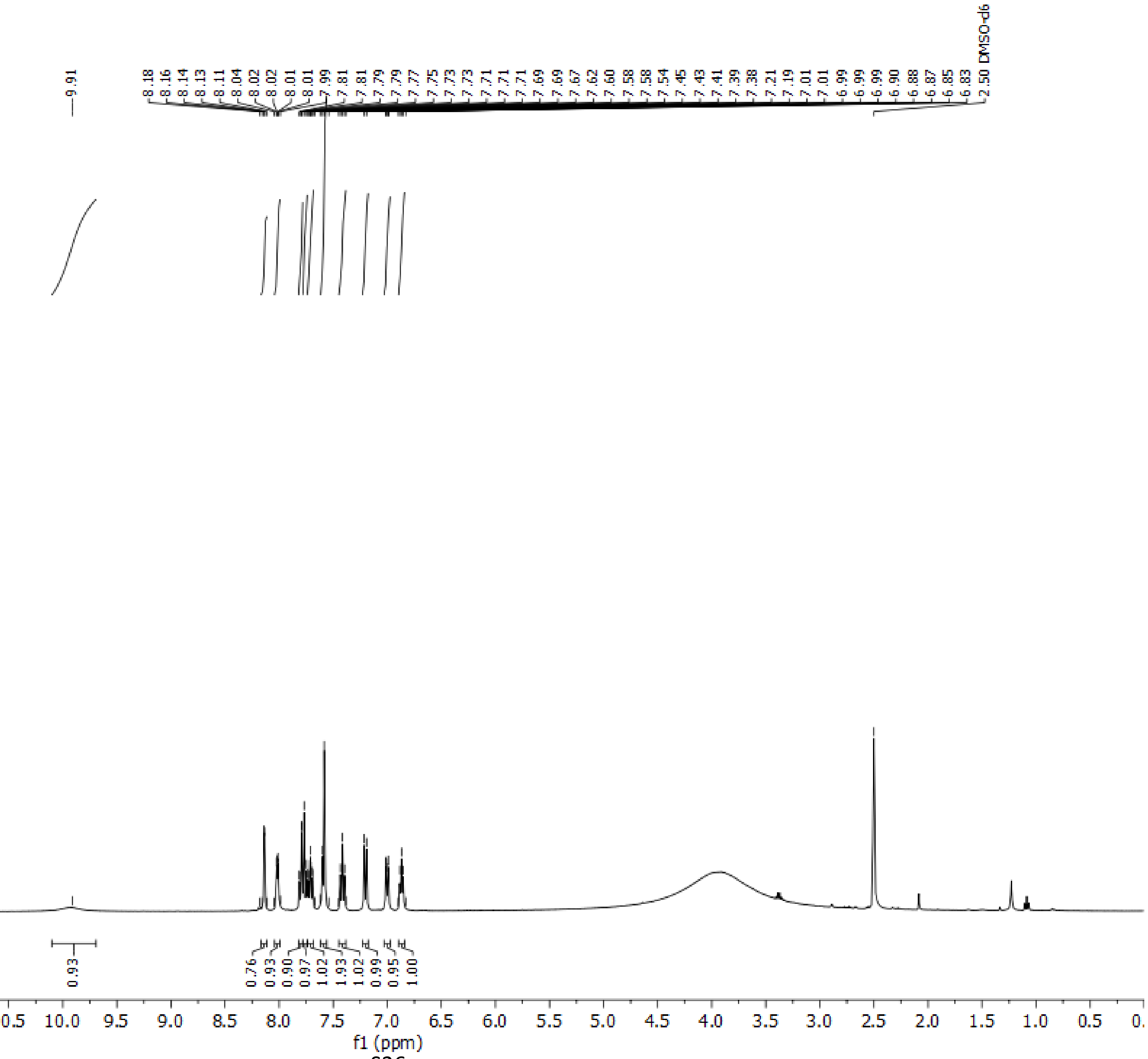

9.91
8.18 8.16 8.14 8.13 8.11 8.04 8.02 8.02 8.01 8.01 7.99 7.81 7.81 7.79 7.79 7.77 7.75 7.73 7.73 7.71 7.71 7.71 7.69 7.69 7.67 7.62 7.60 7.58 7.58 7.54 7.45 7.43 7.41 7.39 7.38 7.21 7.19 7.01 7.01 6.99 6.99 6.99 6.90 6.88 6.87 6.85 6.83
2.50 DMSO-d6
0.93
0.76 0.93 0.90 0.97 1.02 1.93 1.02 0.99 0.95 1.00
13.5 13.0 12.5 12.0 11.5 11.0 10.5 10.0 9.5 9.0 8.5 8.0 7.5 7.0 6.5 6.0 5.5 5.0 4.5 4.0 3.5 3.0 2.5 2.0 1.5 1.0 0.5 0.
f1 (ppm)

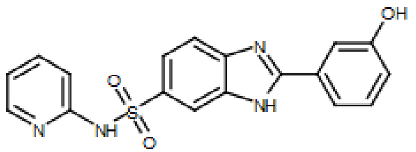

9m

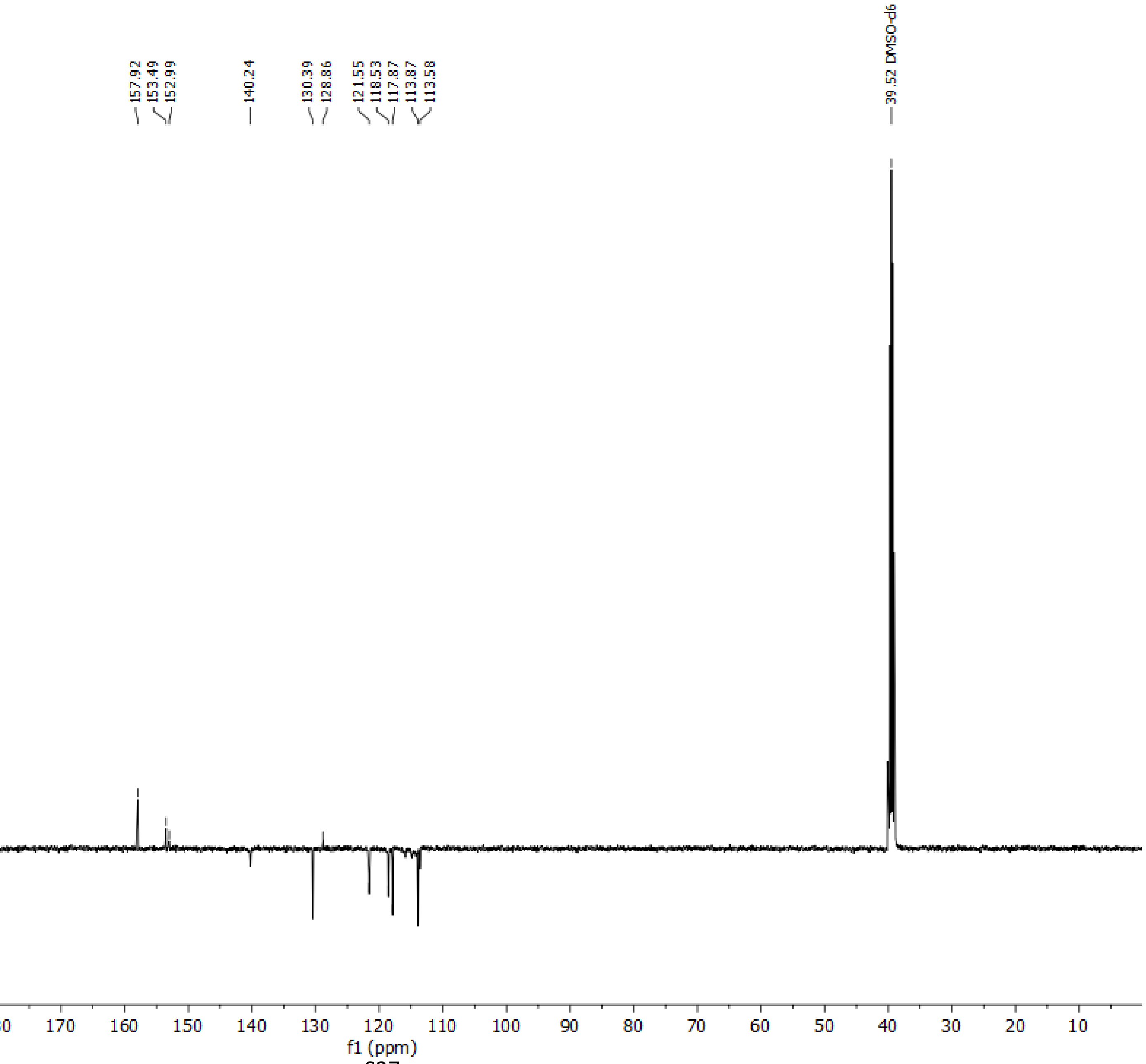
157.92
153.49
152.99
140.24
130.39
128.86
121.55
118.53
117.87
113.87
113.58
39.52 DMSO-d6
230 220 210 200 190 180 170 160 150 140 130 120 110 100 90 80 70 60 50 40 30 20 10
f1 (ppm)

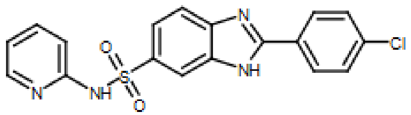

9n

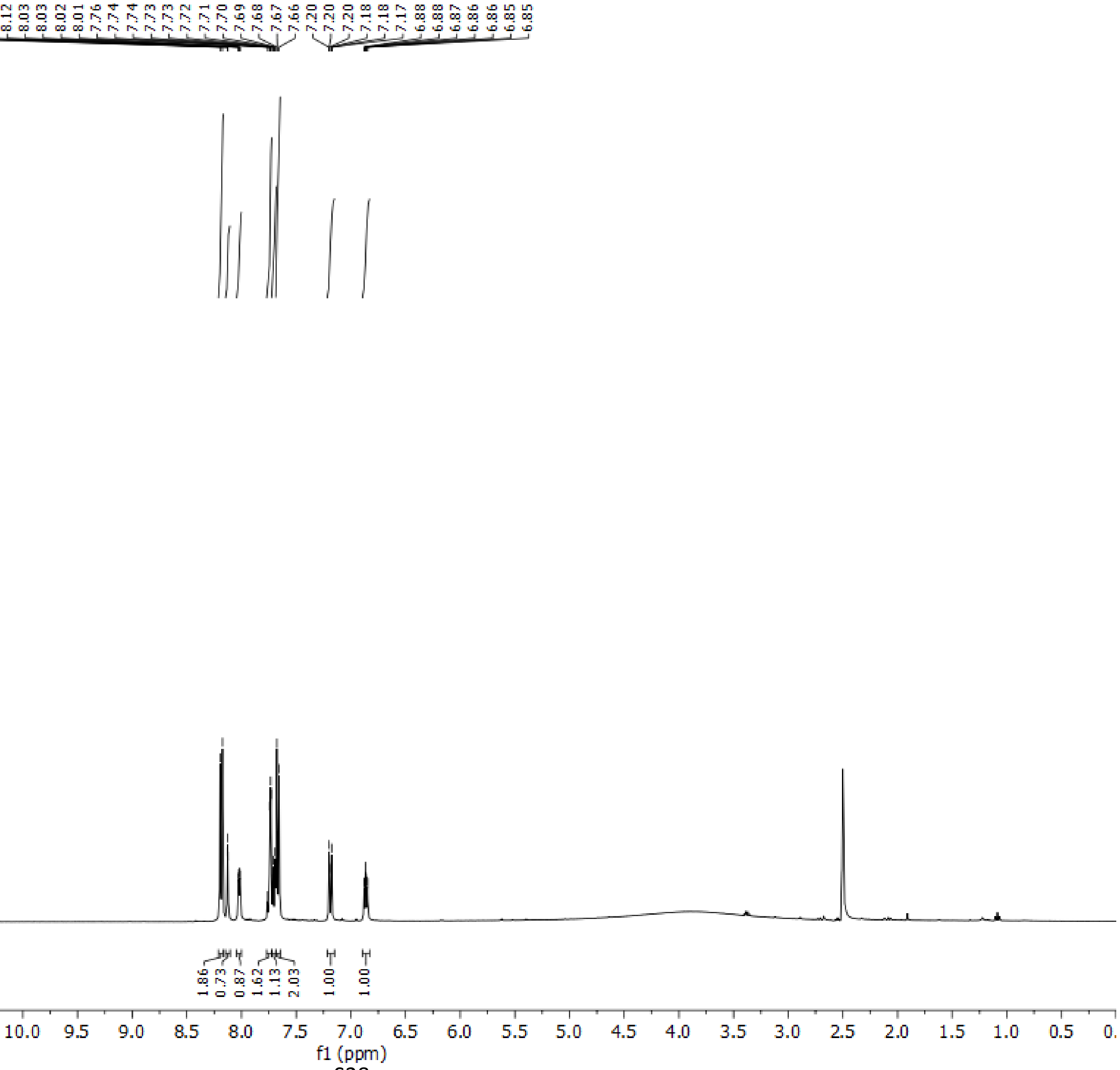
8.20 8.17 8.13 8.13 8.12 8.03 8.03 8.02 8.01 7.76 7.74 7.74 7.73 7.73 7.72 7.71 7.70 7.69 7.68 7.67 7.66 7.20 7.20 7.20 7.18 7.17 6.88 6.88 6.87 6.86 6.86 6.85 6.85
1.86 0.73 0.87 1.62 1.13 2.03 1.00 1.00
13.5 13.0 12.5 12.0 11.5 11.0 10.5 10.0 9.5 9.0 8.5 8.0 7.5 7.0 6.5 6.0 5.5 5.0 4.5 4.0 3.5 3.0 2.5 2.0 1.5 1.0 0.5
f1 (ppm)

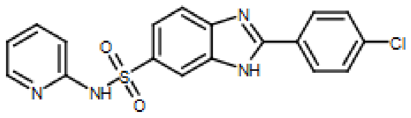

9n

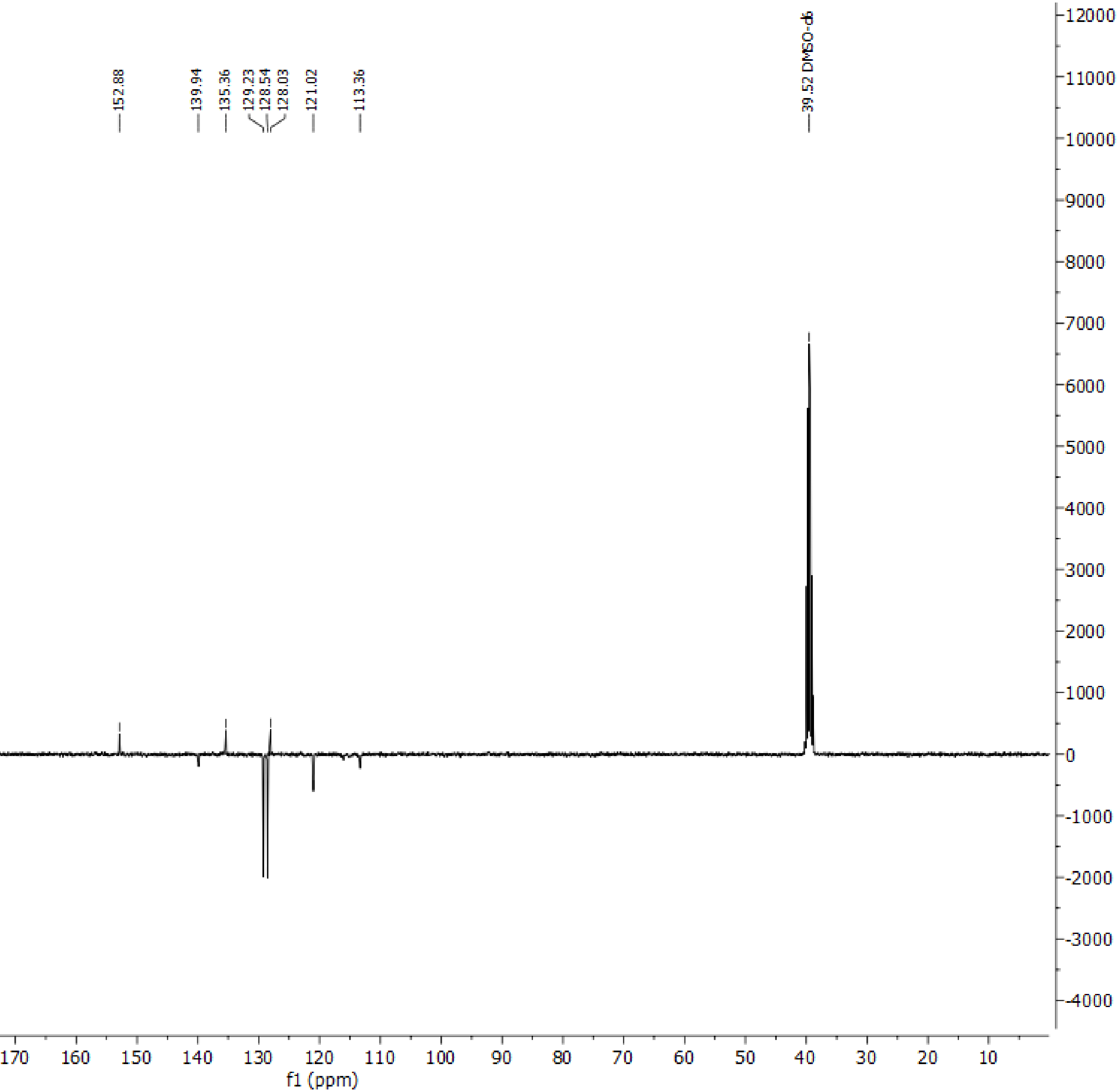

152.88
139.94
135.36
129.23
128.54
128.03
121.02
113.36
39.52 DMSO-d6
12000
11000
10000
9000
8000
7000
6000
5000
4000
3000
2000
1000
0
-1000
-2000
-3000
-4000
230
220
210
200
190
180
170
160
150
140
130
120
110
100
90
80
70
60
50
40
30
20
10
f1 (ppm)

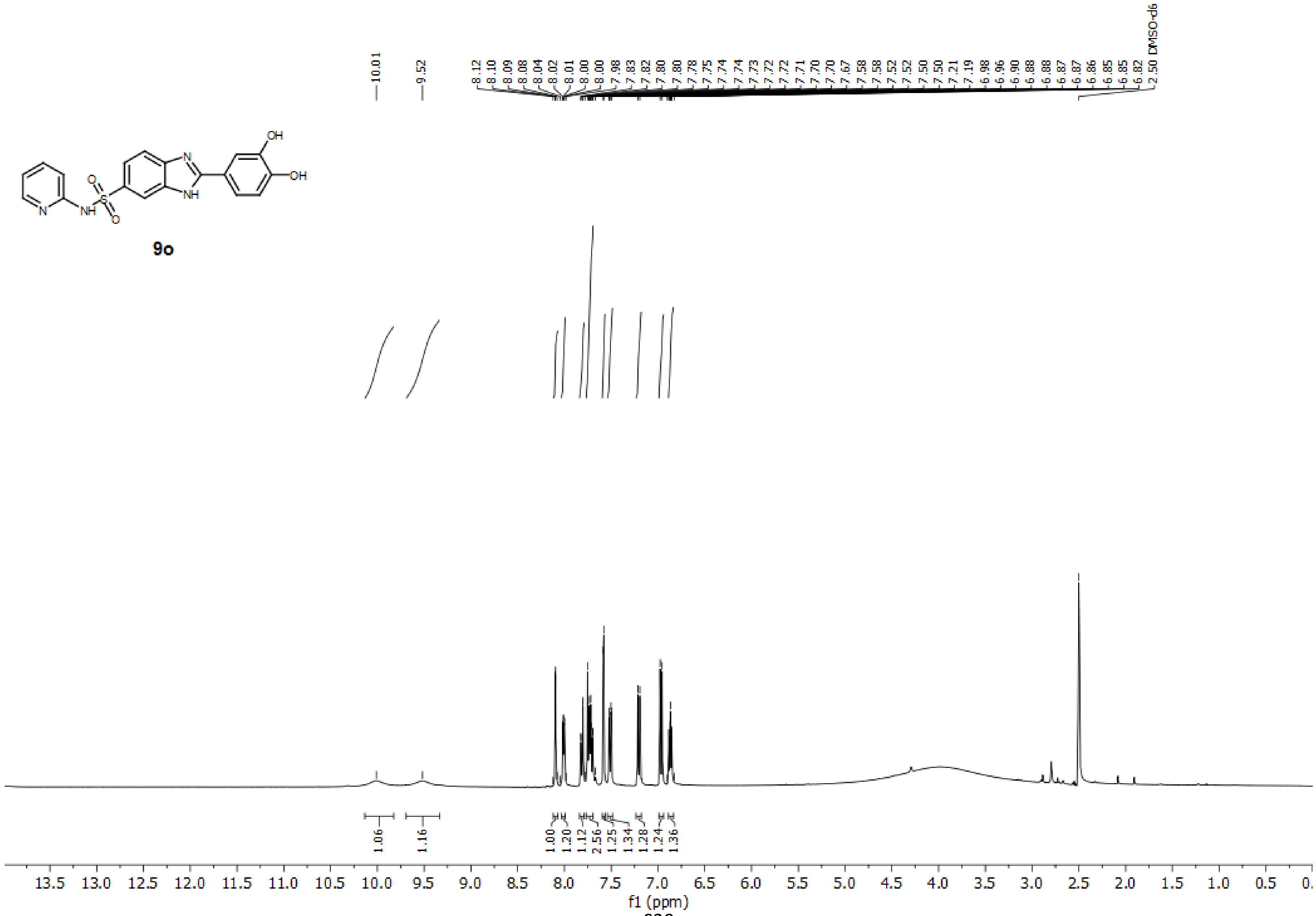
9o
OH
OH
NH
N
NH
N
O
S
O
10.01
9.52
8.12
8.10
8.09
8.08
8.04
8.02
8.01
8.00
8.00
7.98
7.83
7.82
7.80
7.80
7.78
7.75
7.74
7.74
7.73
7.72
7.72
7.71
7.70
7.70
7.67
7.58
7.58
7.52
7.52
7.50
7.50
7.21
7.19
6.98
6.96
6.90
6.88
6.88
6.87
6.87
6.86
6.85
6.85
6.82
2.50 DMSO-d6
1.06
1.16
1.00
1.20
1.12
2.56
1.25
1.34
1.28
1.24
1.36
f1 (ppm)

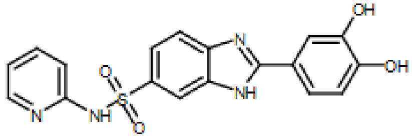
OH
OH
N
NH
O
S
NH
O
N

9o

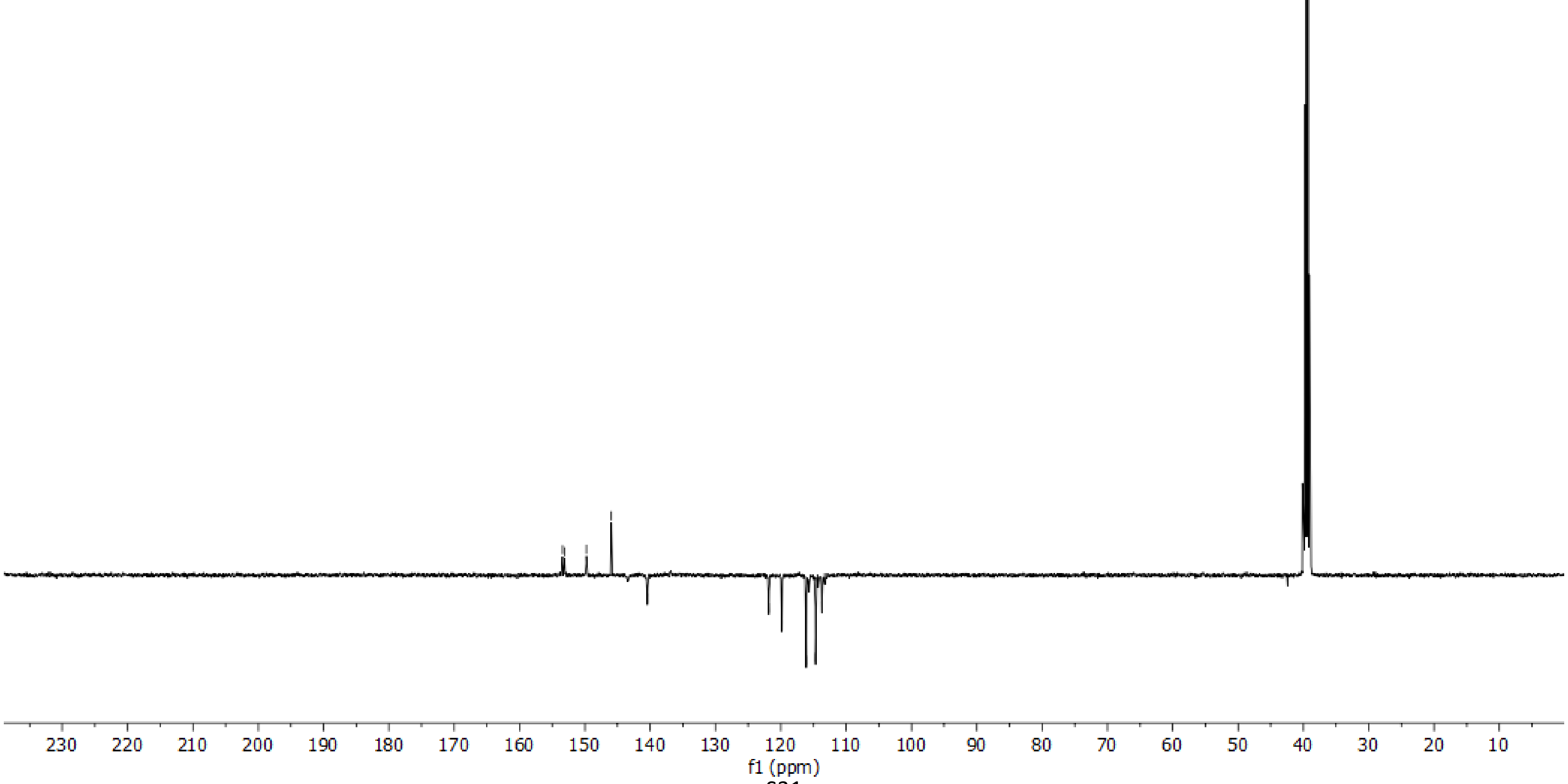
153.45
153.09
149.75
145.95
140.43
121.86
119.82
116.12
115.71
114.68
114.35
113.71
113.29
39.52 DMSO-d6
230 220 210 200 190 180 170 160 150 140 130 120 110 100 90 80 70 60 50 40 30 20 10
f1 (ppm)

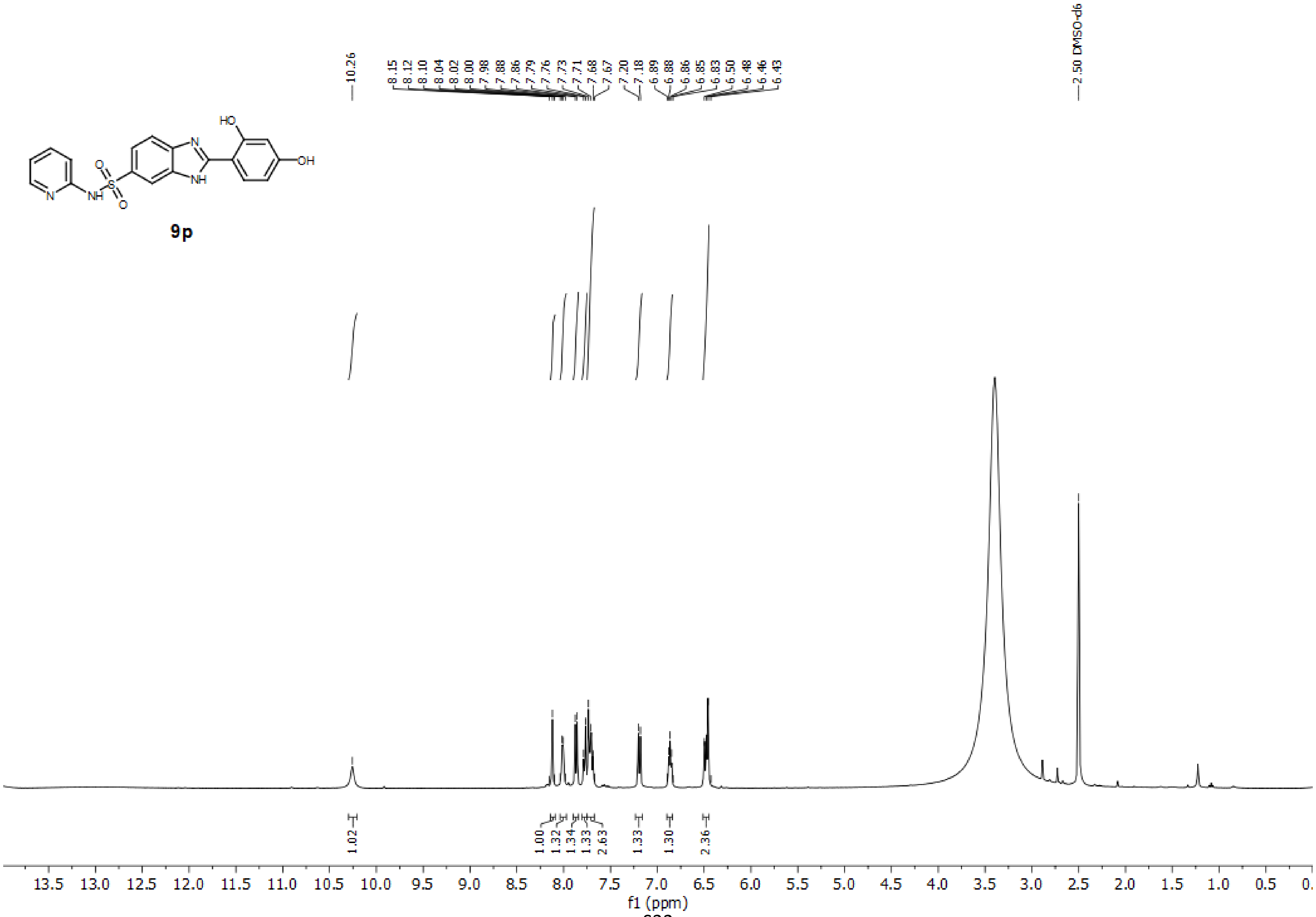

10.26
8.15
8.12
8.10
8.04
8.02
8.00
7.98
7.88
7.86
7.79
7.76
7.73
7.71
7.68
7.67
7.20
7.18
6.89
6.88
6.86
6.85
6.83
6.50
6.48
6.46
6.43
2.50 DMSO-d6
1.02
1.00
1.32
1.34
1.33
2.63
1.33
1.30
2.36
f1 (ppm)

9p

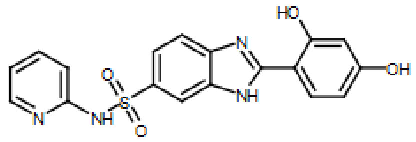

HO
OH
N
NH
O
S
O
NH
N

9p

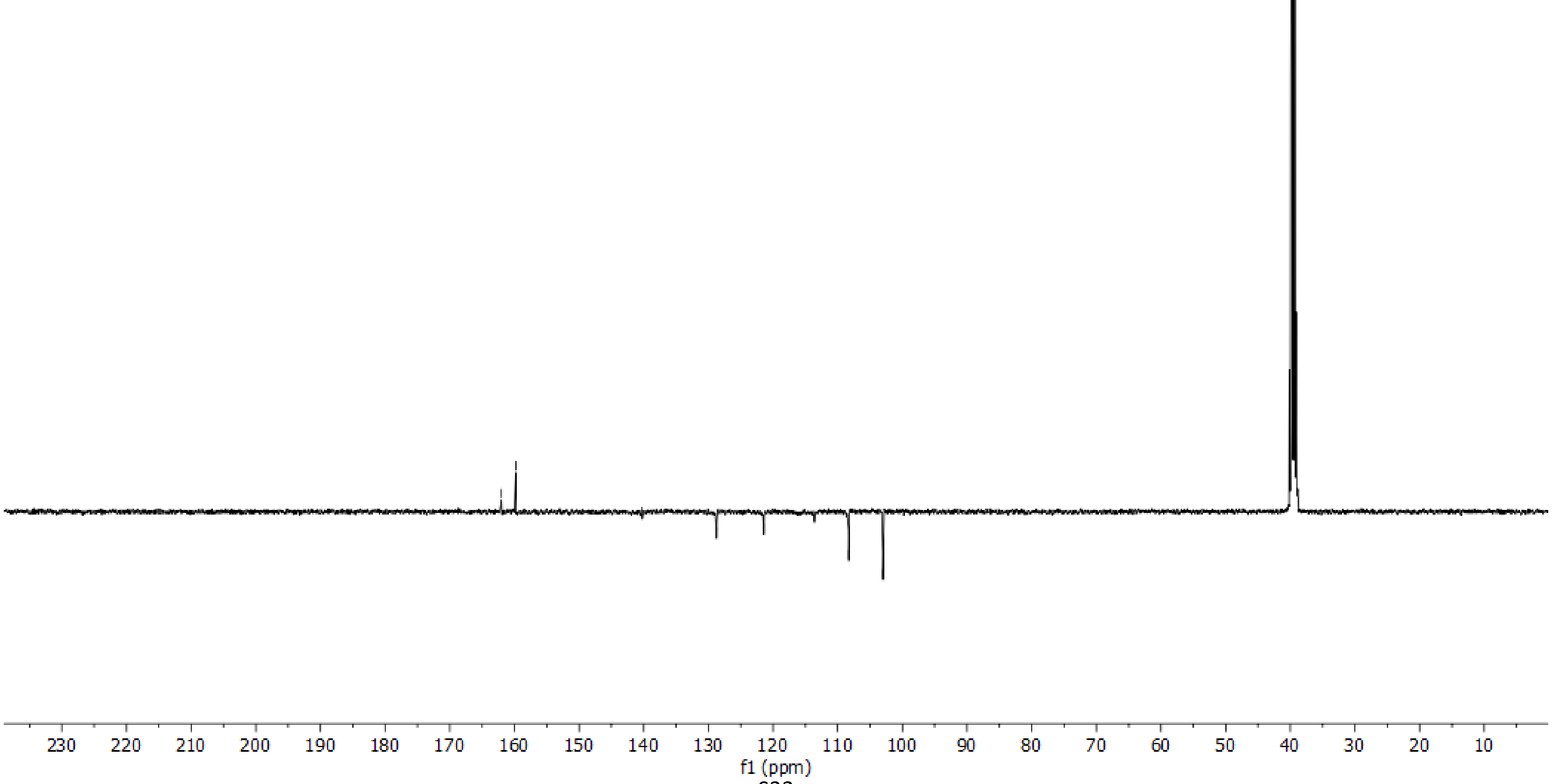

162.03
159.80
140.27
128.77
121.43
113.63
108.28
103.02
230 220 210 200 190 180 170 160 150 140 130 120 110 100 90 80 70 60 50 40 30 20 10
f1 (ppm)

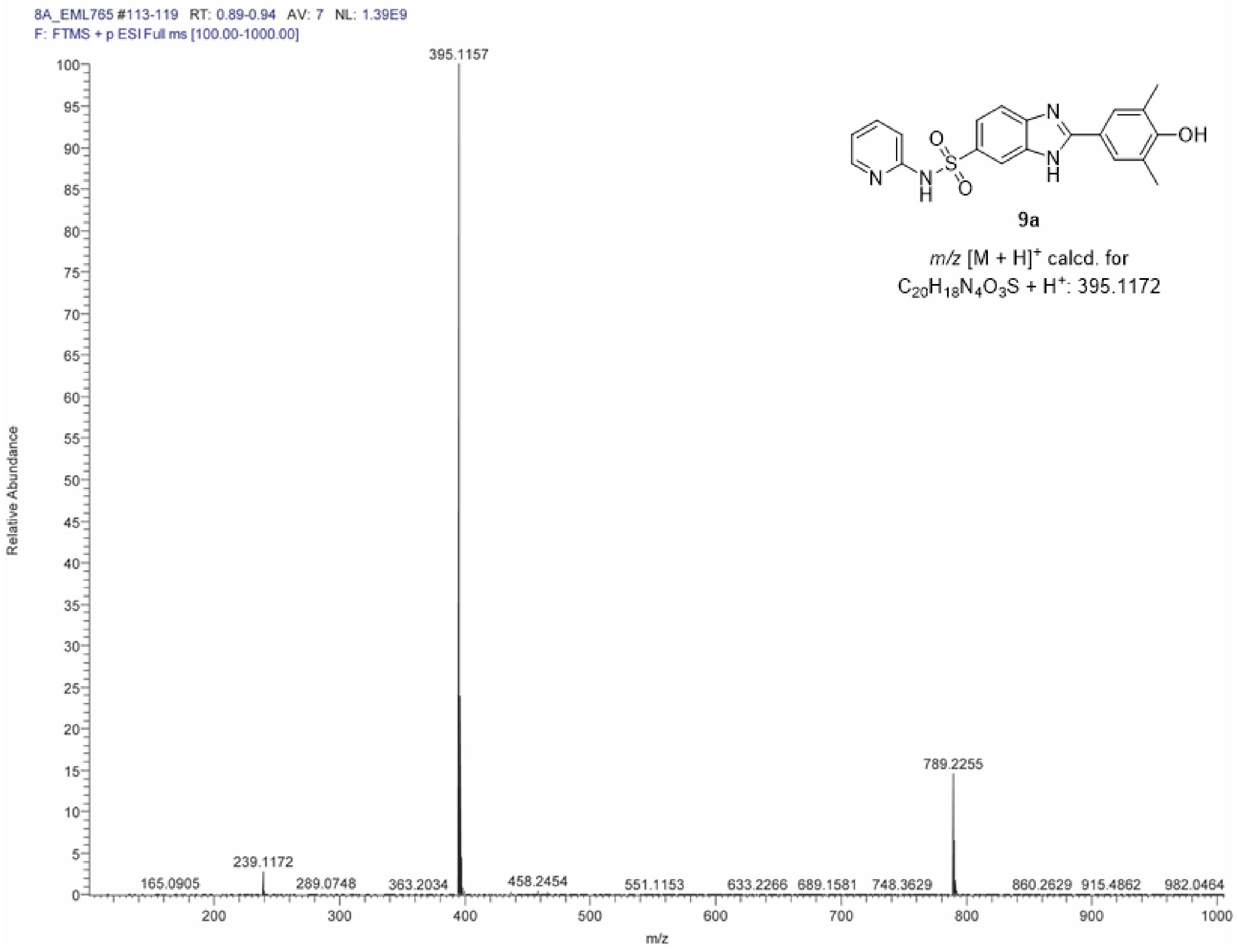

9a

m/z $[M + H]^+$ calcd. for $C_{20}H_{18}N_4O_3S + H^+$: 395.1172

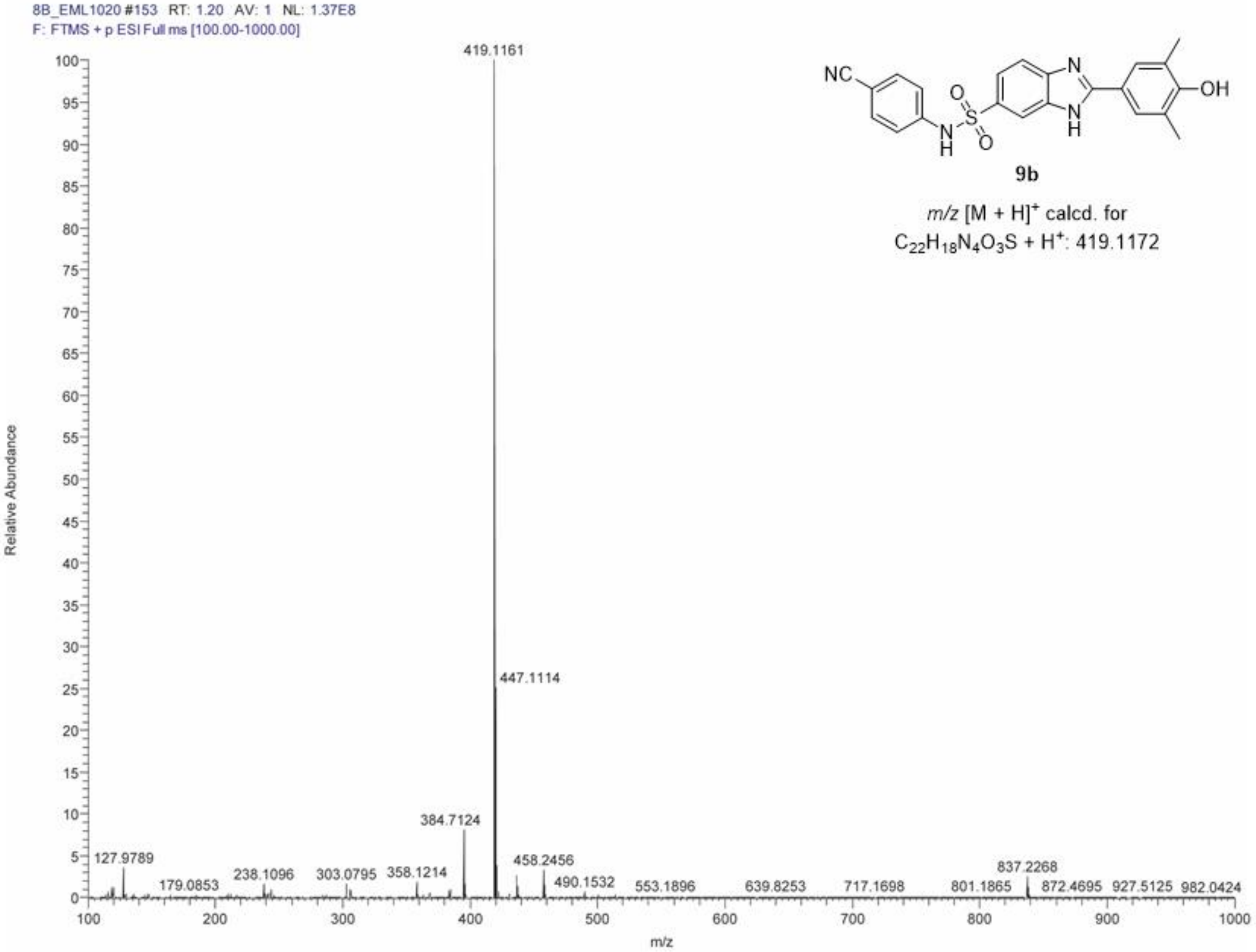
8B_EML1020 #153 RT: 1.20 AV: 1 NL: 1.37E8
F: FTMS + p ESI Full ms [100.00-1000.00]
419.1161
447.1114
384.7124
127.9789
179.0853
238.1096
303.0795
358.1214
458.2456
490.1532
553.1896
639.8253
717.1698
801.1865
837.2268
872.4695
927.5125
982.0424
Relative Abundance
m/z
9b
m/z [M + H]+ calcd. for
C22H18N4O3S + H+: 419.1172

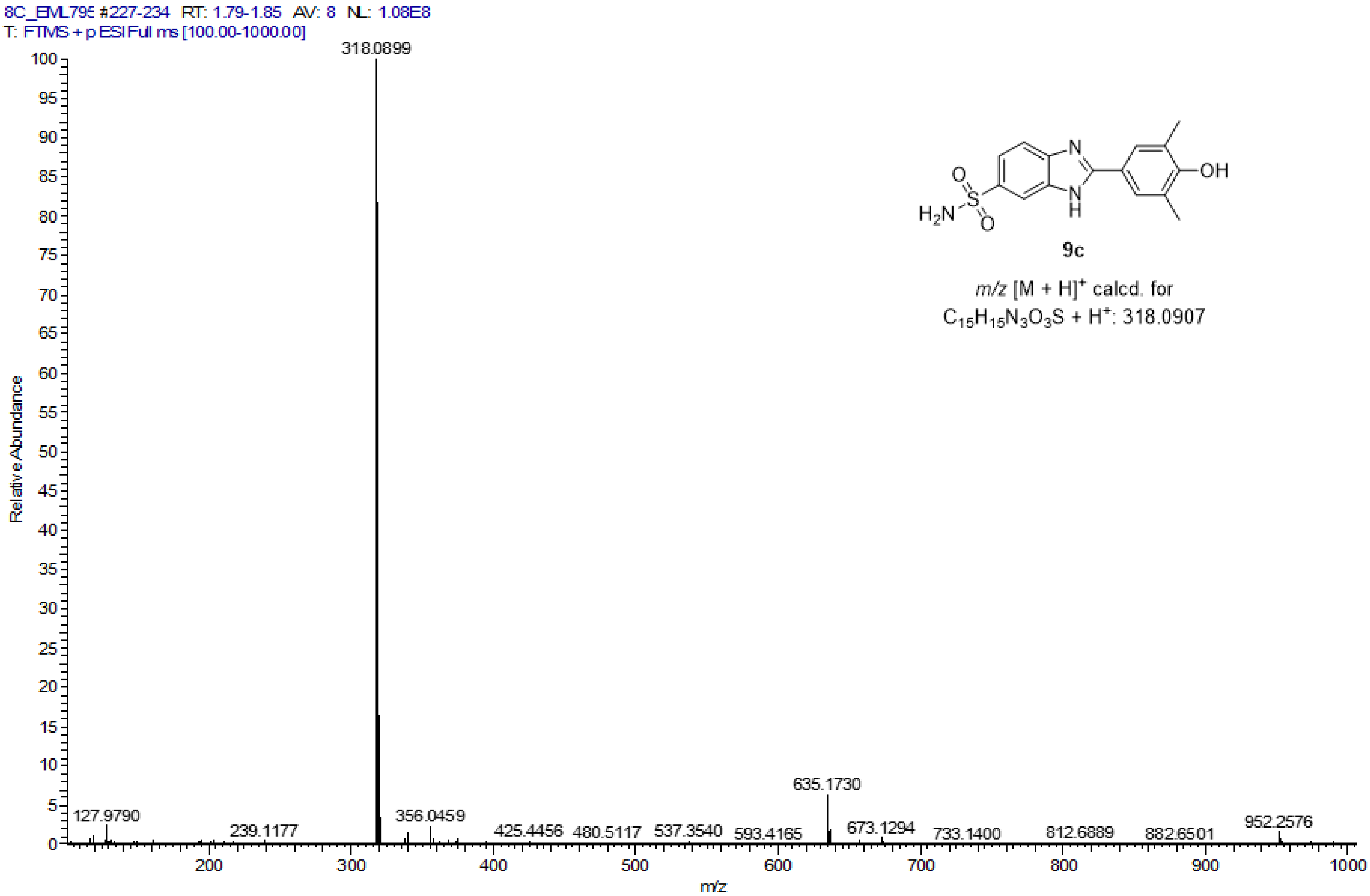

9c

m/z $[M + H]^+$ calcd. for $C_{15}H_{15}N_3O_3S + H^+$: 318.0907

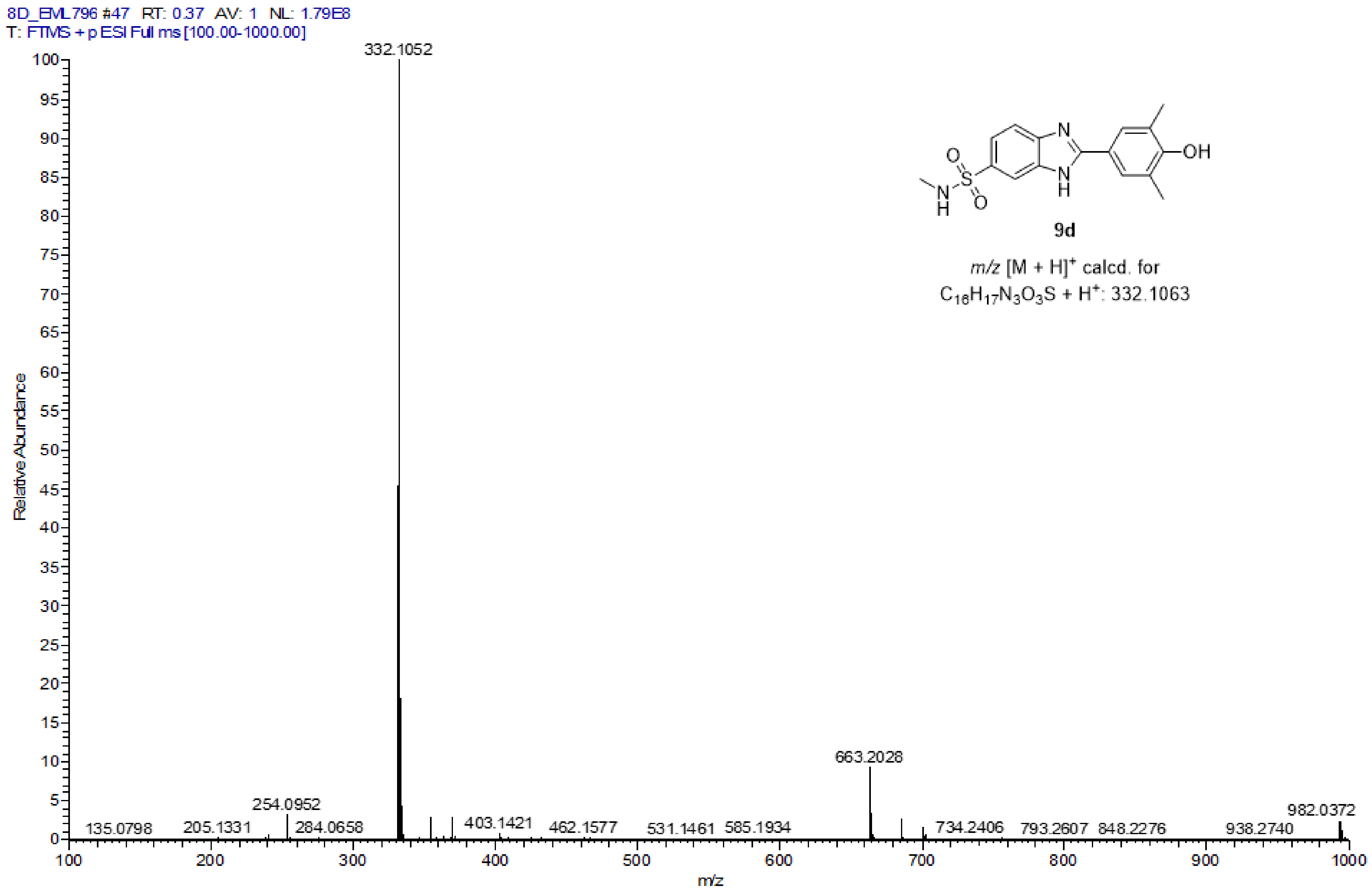
8D_EML796 #47 RT: 0.37 AV: 1 NL: 1.79E8
T: FTMS + p ESI Full ms [100.00-1000.00]
332.1052
663.2028
254.0952
982.0372
135.0798
205.1331
284.0658
403.1421
462.1577
531.1461
585.1934
734.2406
793.2607
848.2276
938.2740
Relative Abundance
m/z
9d
m/z [M + H]+ calcd. for
C16H17N3O3S + H+: 332.1063

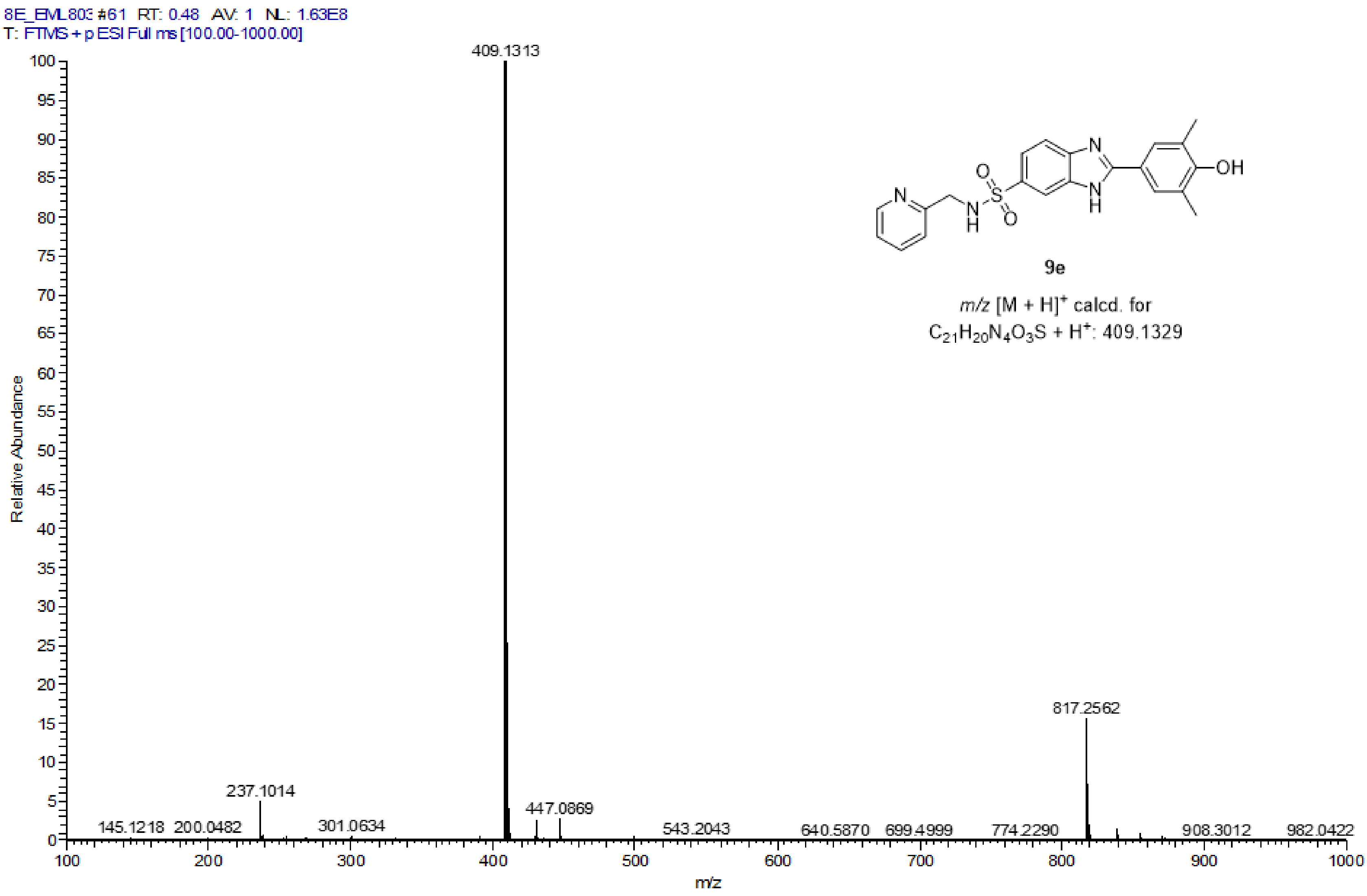

8E_EML803 #61 RT: 0.48 AV: 1 NL: 1.63E8
T: FTMS + p ESI Full ms [100.00-1000.00]
409.1313
817.2562
237.1014
447.0869
145.1218
200.0482
301.0634
543.2043
640.5870
699.4999
774.2290
908.3012
982.0422
Relative Abundance
m/z
9e
m/z $[M + H]^+$ calcd. for
$C_{21}H_{20}N_4O_3S + H^+$: 409.1329

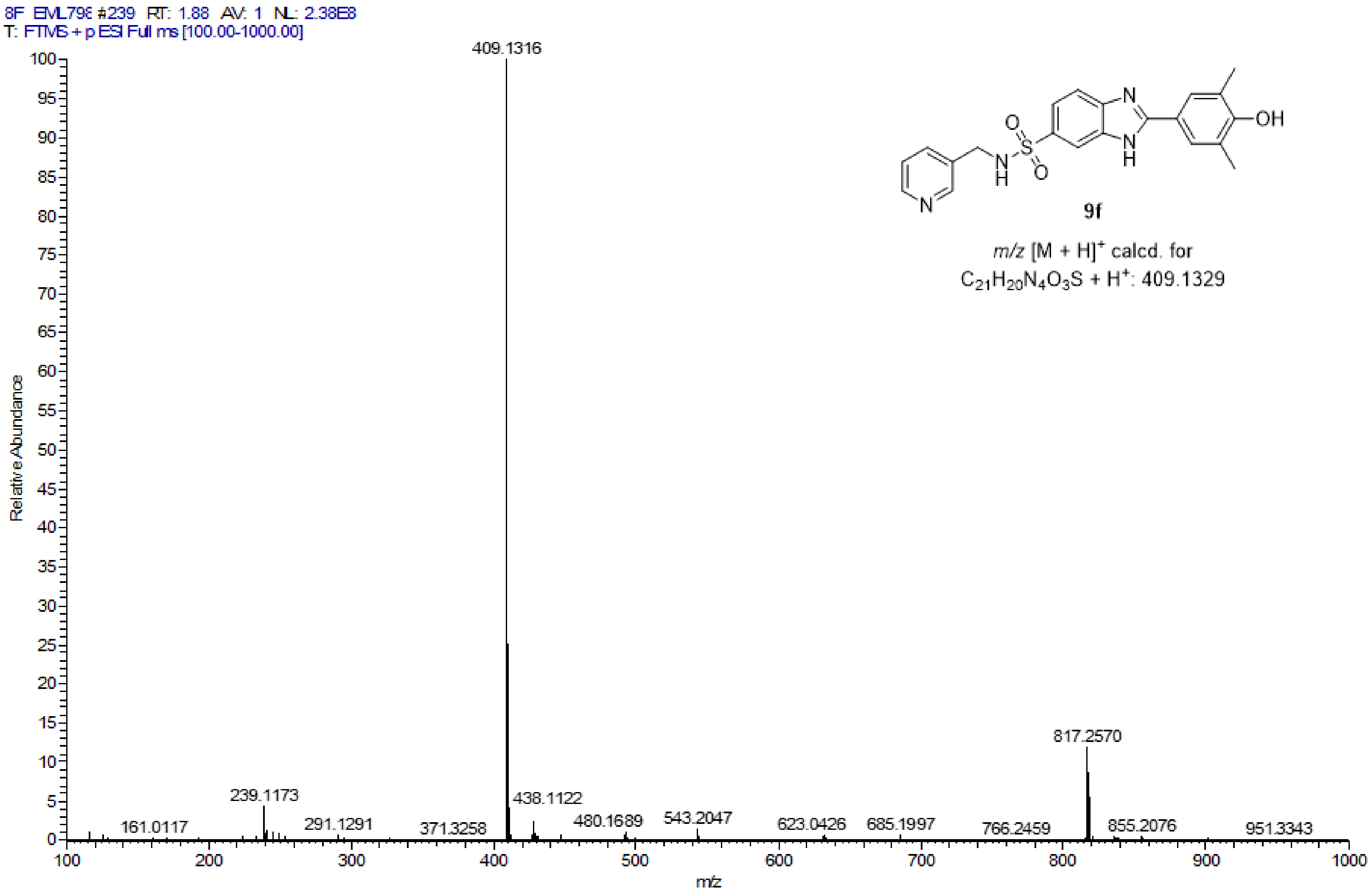
8F EML798 #239 RT: 1.88 AV: 1 NL: 2.38E8
T: FTMS + p ESI Full ms [100.00-1000.00]
409.1316
817.2570
239.1173
438.1122
480.1689
543.2047
161.0117
291.1291
371.3258
623.0426
685.1997
766.2459
855.2076
951.3343
Relative Abundance
m/z
9f
m/z [M + H]+ calcd. for
C21H20N4O3S + H+: 409.1329

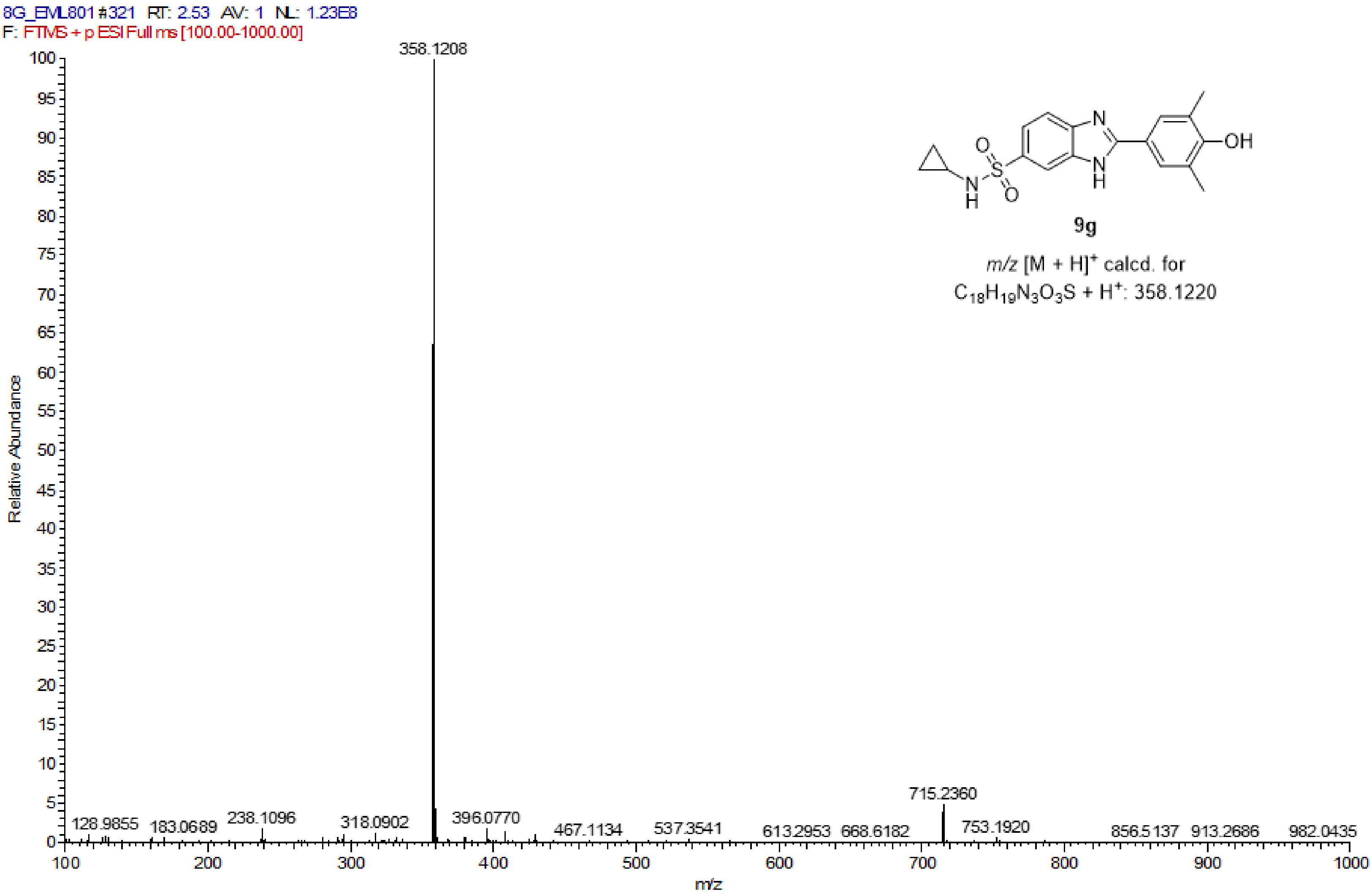

9g

m/z [M + H]$^+$ calcd. for

$C_{18}H_{19}N_3O_3S + H^+$: 358.1220

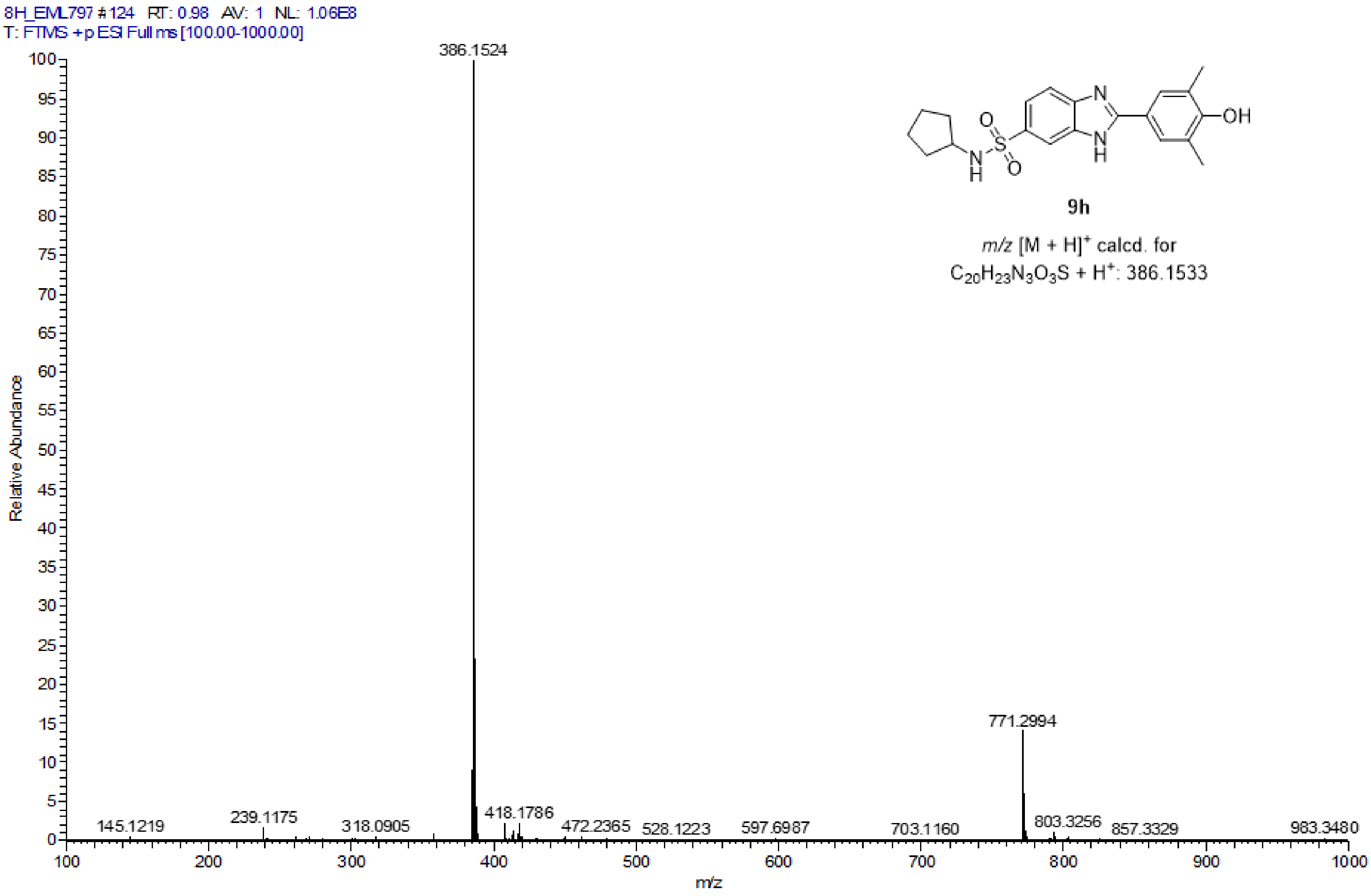

9h

m/z $[M + H]^+$ calcd. for $C_{20}H_{23}N_3O_3S + H^+$: 386.1533

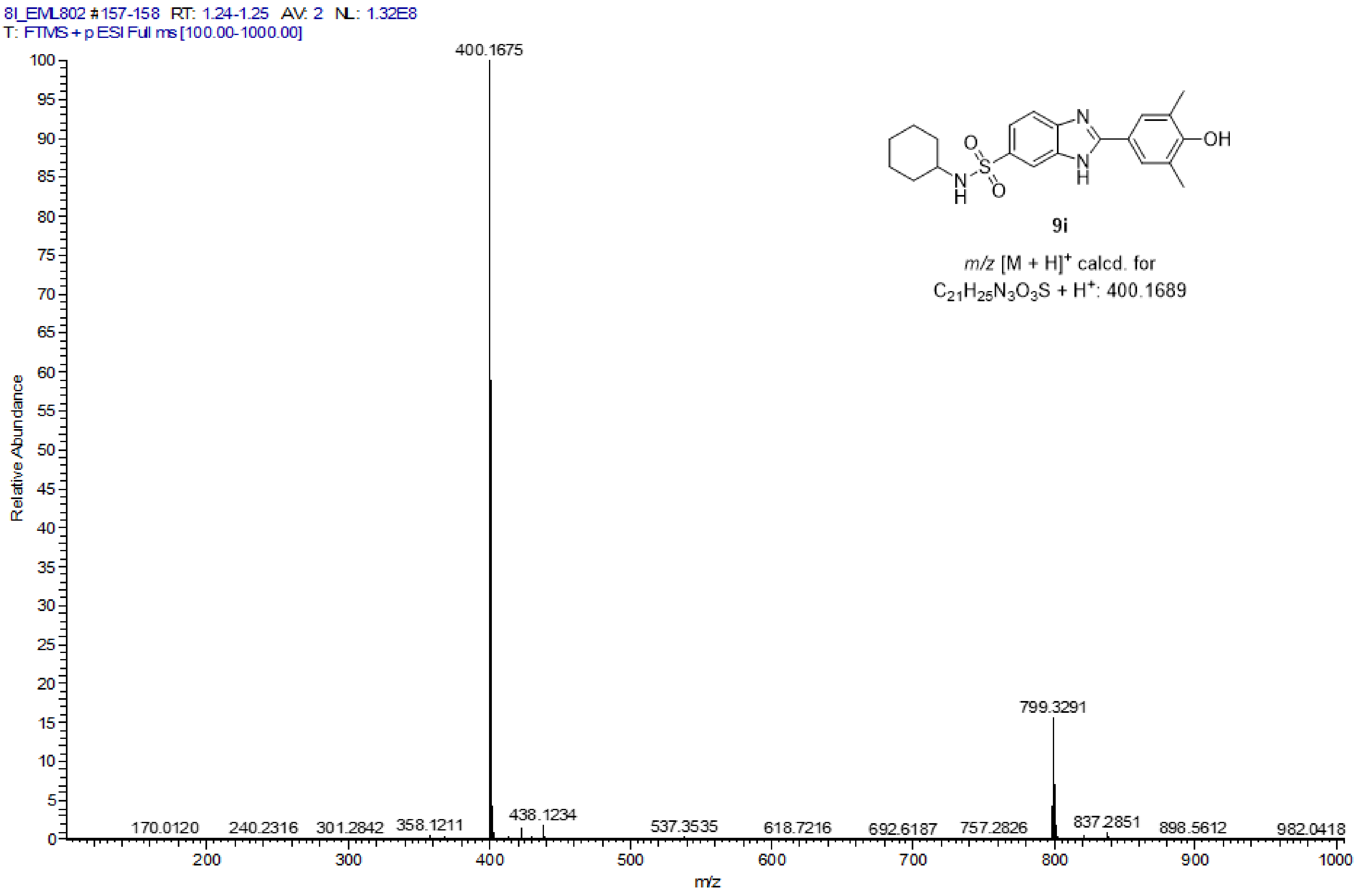

9i

m/z [M + H]$^+$ calcd. for
$C_{21}H_{25}N_3O_3S + H^+$: 400.1689

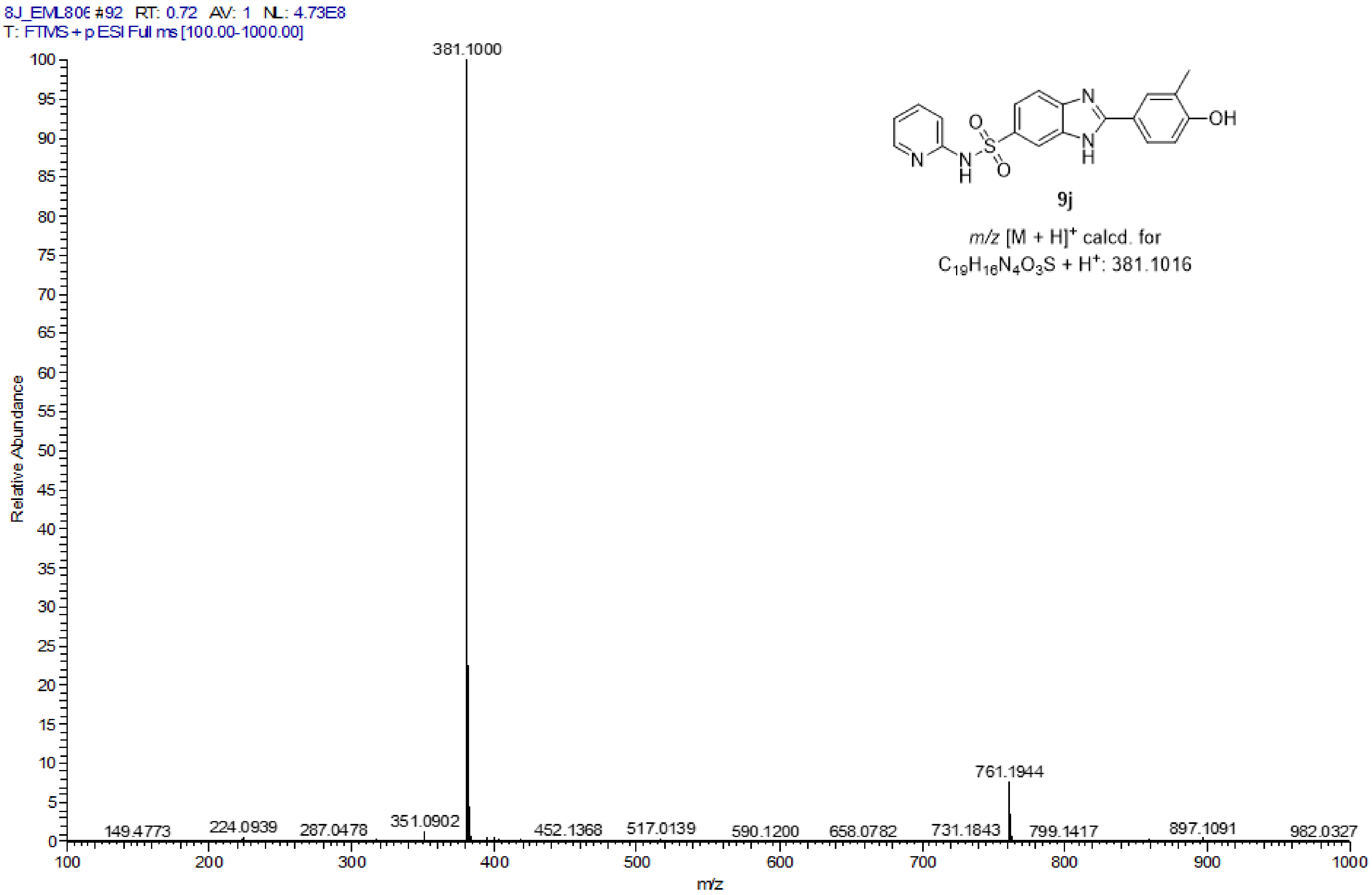
8J_EML806 #92 RT: 0.72 AV: 1 NL: 4.73E8
T: FTMS + p ESI Full ms [100.00-1000.00]
381.1000
761.1944
149.4773
224.0939
287.0478
351.0902
452.1368
517.0139
590.1200
658.0782
731.1843
799.1417
897.1091
982.0327
Relative Abundance
m/z
9j
m/z [M + H]+ calcd. for
C19H16N4O3S + H+: 381.1016

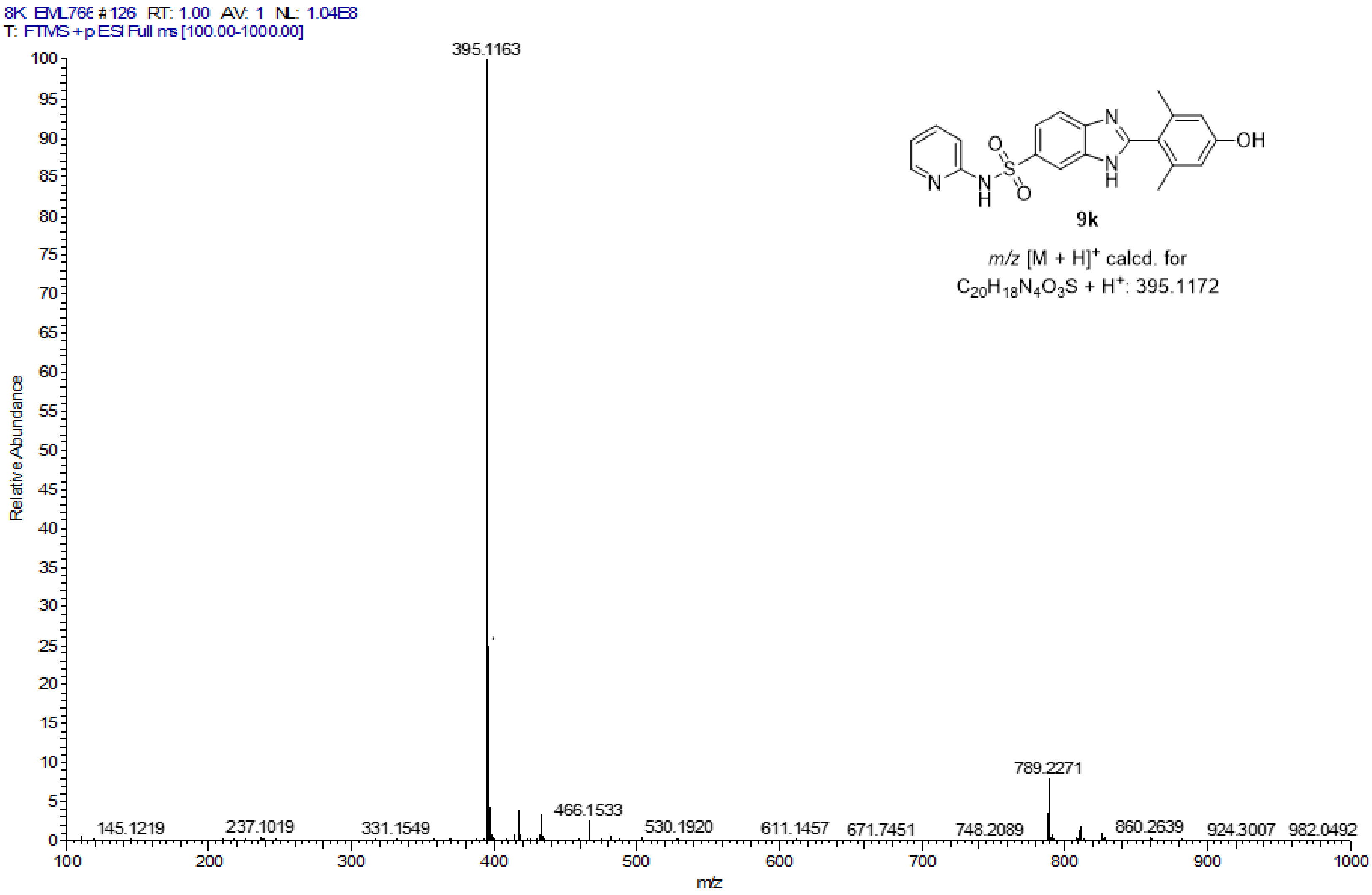
8K EML766 #126 RT: 1.00 AV: 1 NL: 1.04E8
T: FTMS + p ESI Full ms [100.00-1000.00]
395.1163
789.2271
466.1533
145.1219
237.1019
331.1549
530.1920
611.1457
671.7451
748.2089
860.2639
924.3007
982.0492
Relative Abundance
m/z
9k
m/z [M + H]+ calcd. for
C20H18N4O3S + H+: 395.1172

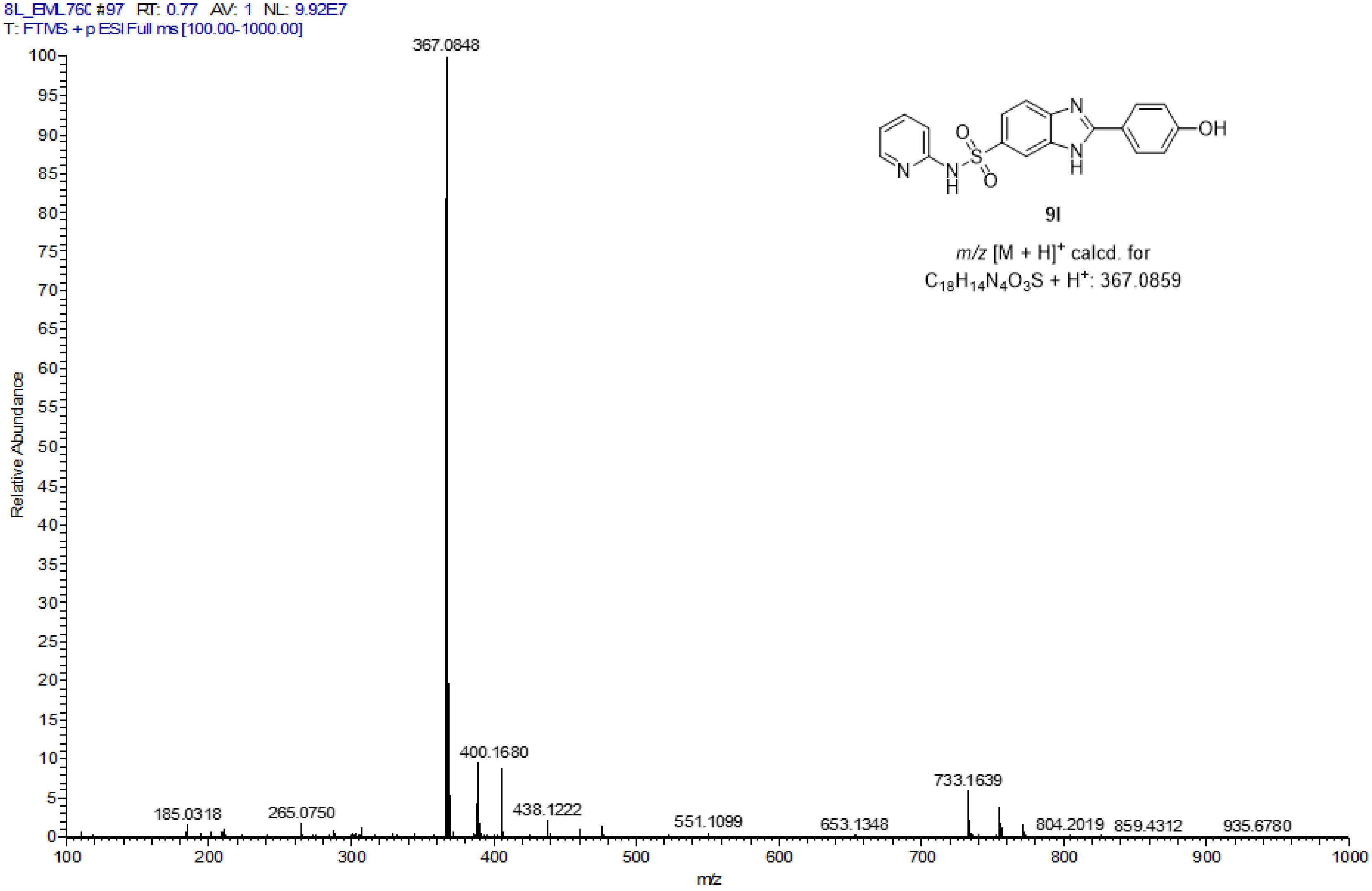

8L_EML760 #97 RT: 0.77 AV: 1 NL: 9.92E7
T: FTMS + p ESI Full ms [100.00-1000.00]
367.0848
400.1680
438.1222
185.0318
265.0750
551.1099
653.1348
733.1639
804.2019
859.4312
935.6780
Relative Abundance
m/z
9l
m/z [M + H]$^+$ calcd. for
$C_{18}H_{14}N_4O_3S + H^+$: 367.0859

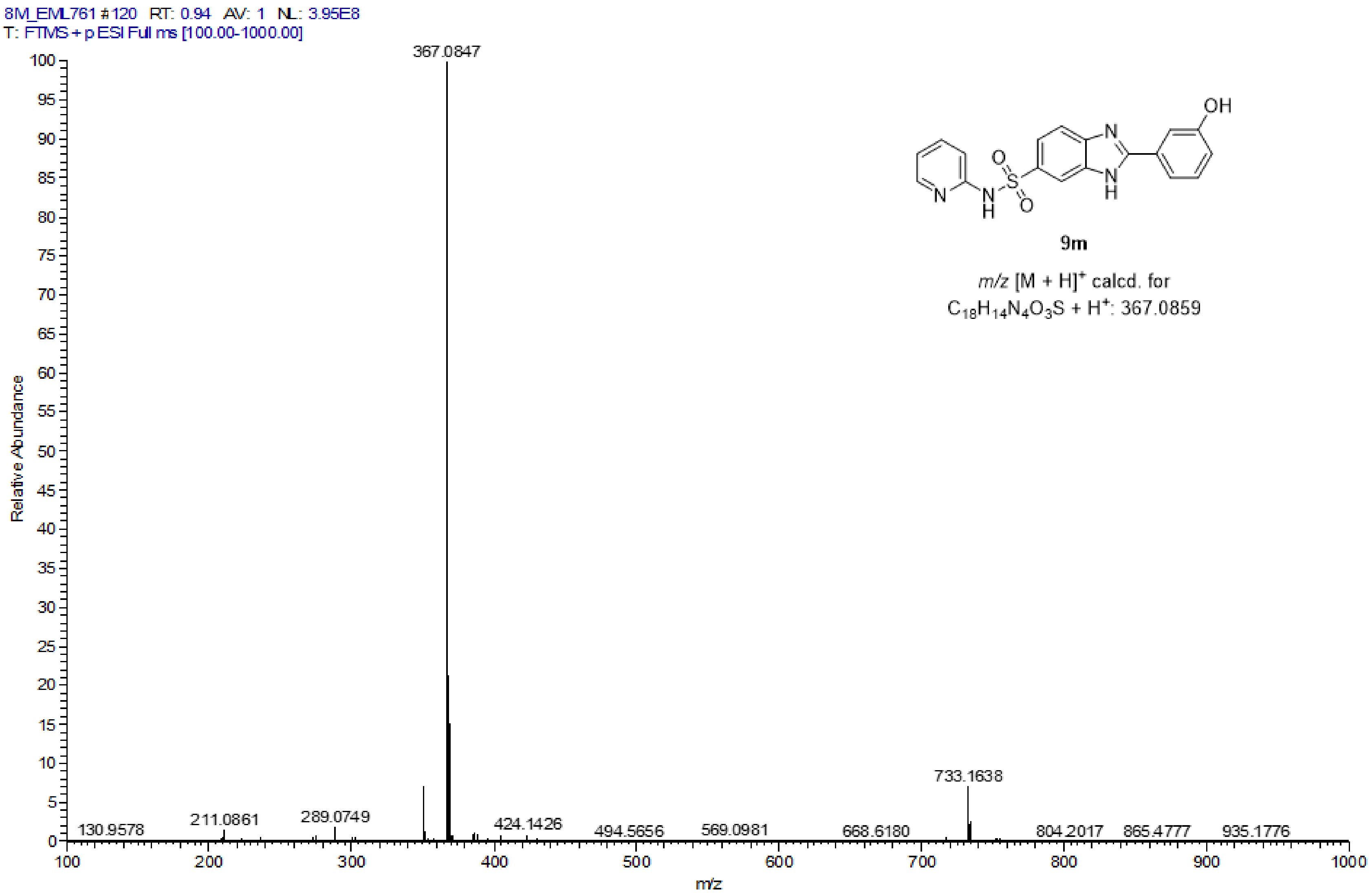
8M_EML761 #120 RT: 0.94 AV: 1 NL: 3.95E8
T: FTMS + p ESI Full ms [100.00-1000.00]
367.0847
733.1638
130.9578
211.0861
289.0749
424.1426
494.5656
569.0981
668.6180
804.2017
865.4777
935.1776
Relative Abundance
m/z
OH
N
N
H
O
S
O
N
H
N
9m
m/z $[M + H]^+$ calcd. for
$C_{18}H_{14}N_4O_3S + H^+$: 367.0859

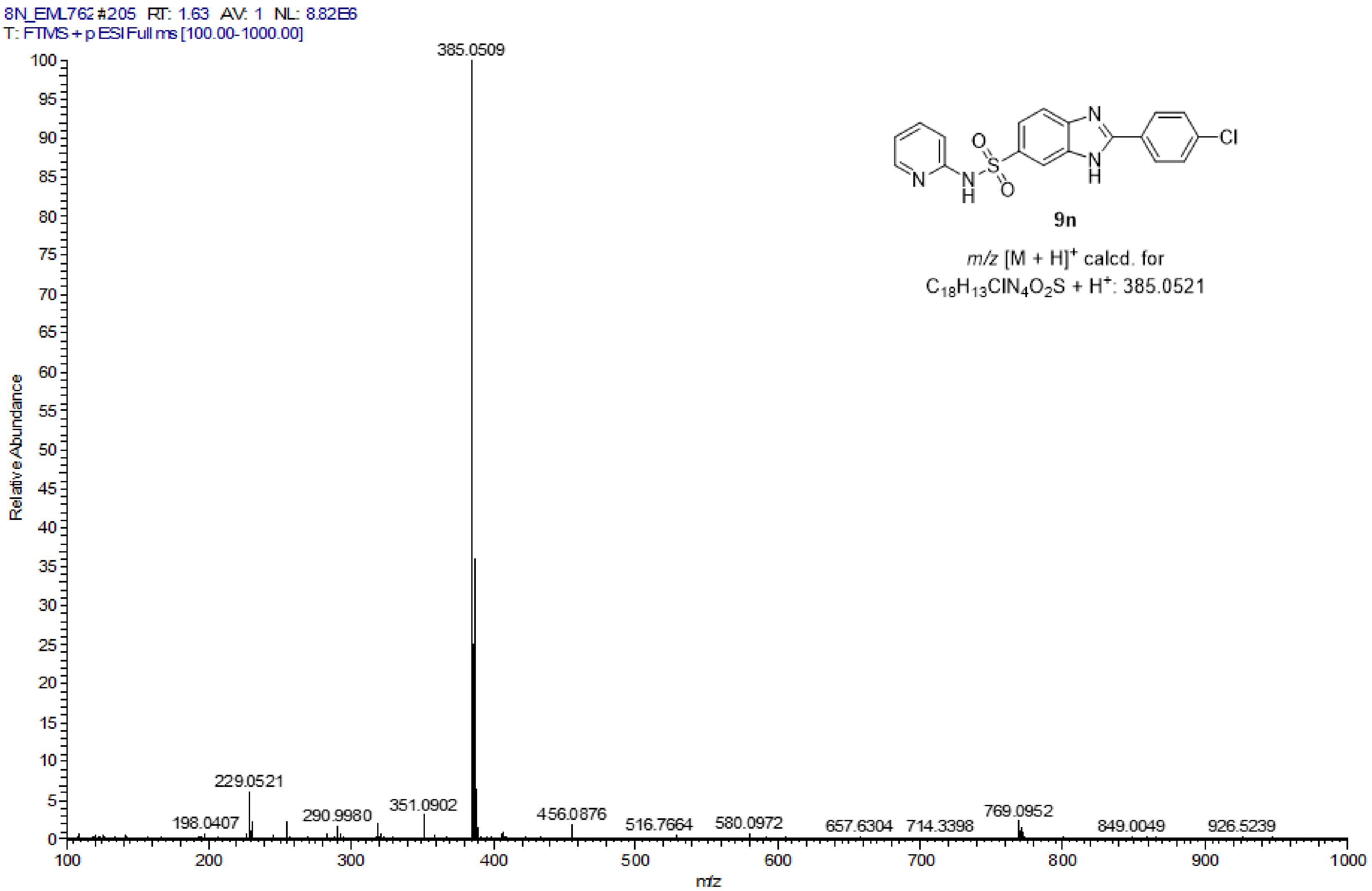
8N_EML762 #205 RT: 1.63 AV: 1 NL: 8.82E6
T: FTMS + p ESI Full ms [100.00-1000.00]
385.0509
229.0521
198.0407
290.9980
351.0902
456.0876
516.7664
580.0972
657.6304
714.3398
769.0952
849.0049
926.5239
Relative Abundance
m/z
N
O
S
O
N
H
N
N
H
Cl
9n
m/z [M + H]+ calcd. for
C18H13ClN4O2S + H+: 385.0521

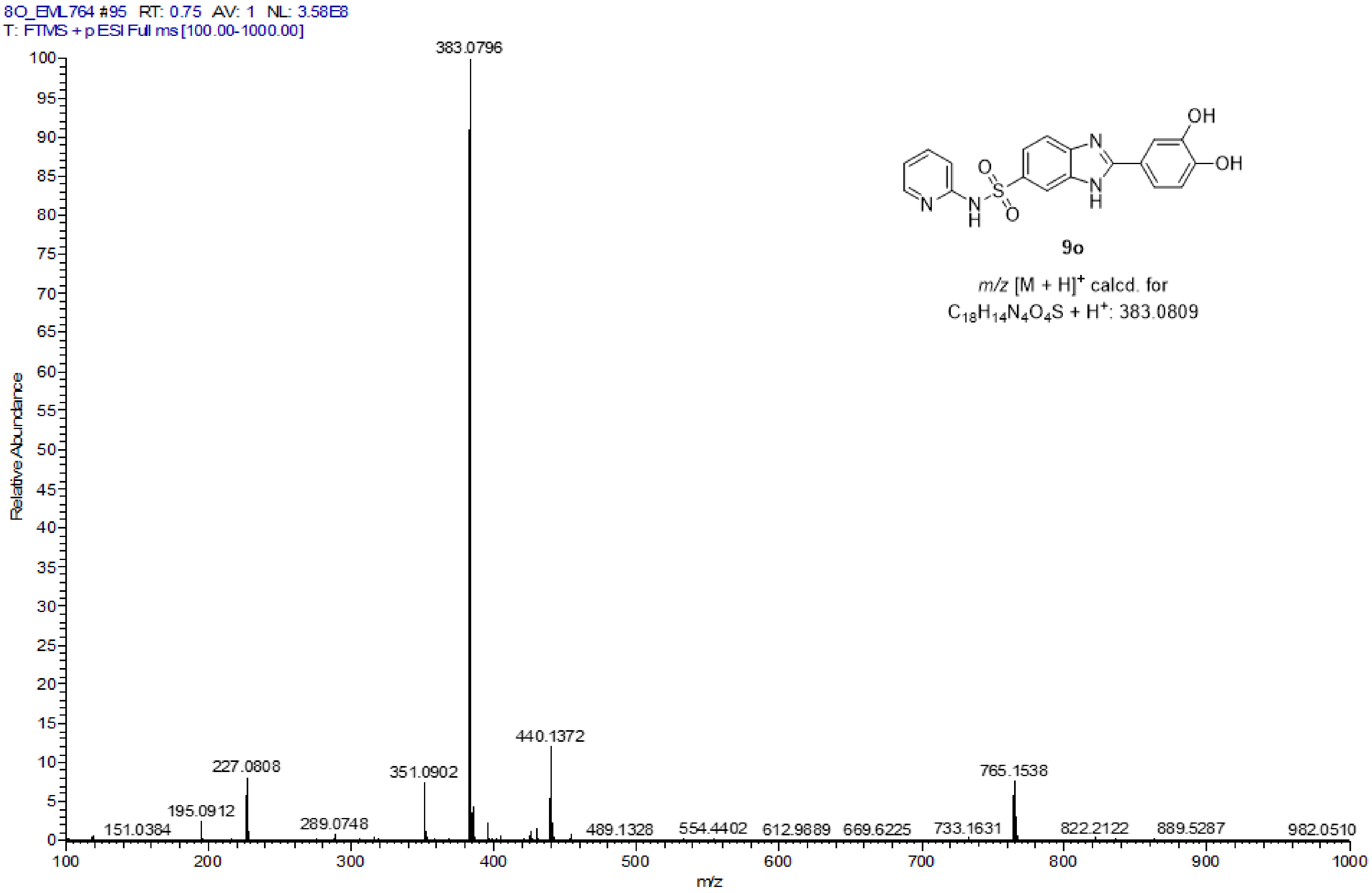
8O_EML764 #95 RT: 0.75 AV: 1 NL: 3.58E8
T: FTMS + p ESI Full ms [100.00-1000.00]
383.0796
440.1372
227.0808
351.0902
765.1538
195.0912
151.0384
289.0748
489.1328
554.4402
612.9889
669.6225
733.1631
822.2122
889.5287
982.0510
Relative Abundance
m/z
OH
OH
N
N
H
O
S
O
N
H
N
9o
m/z [M + H]+ calcd. for
C18H14N4O4S + H+: 383.0809

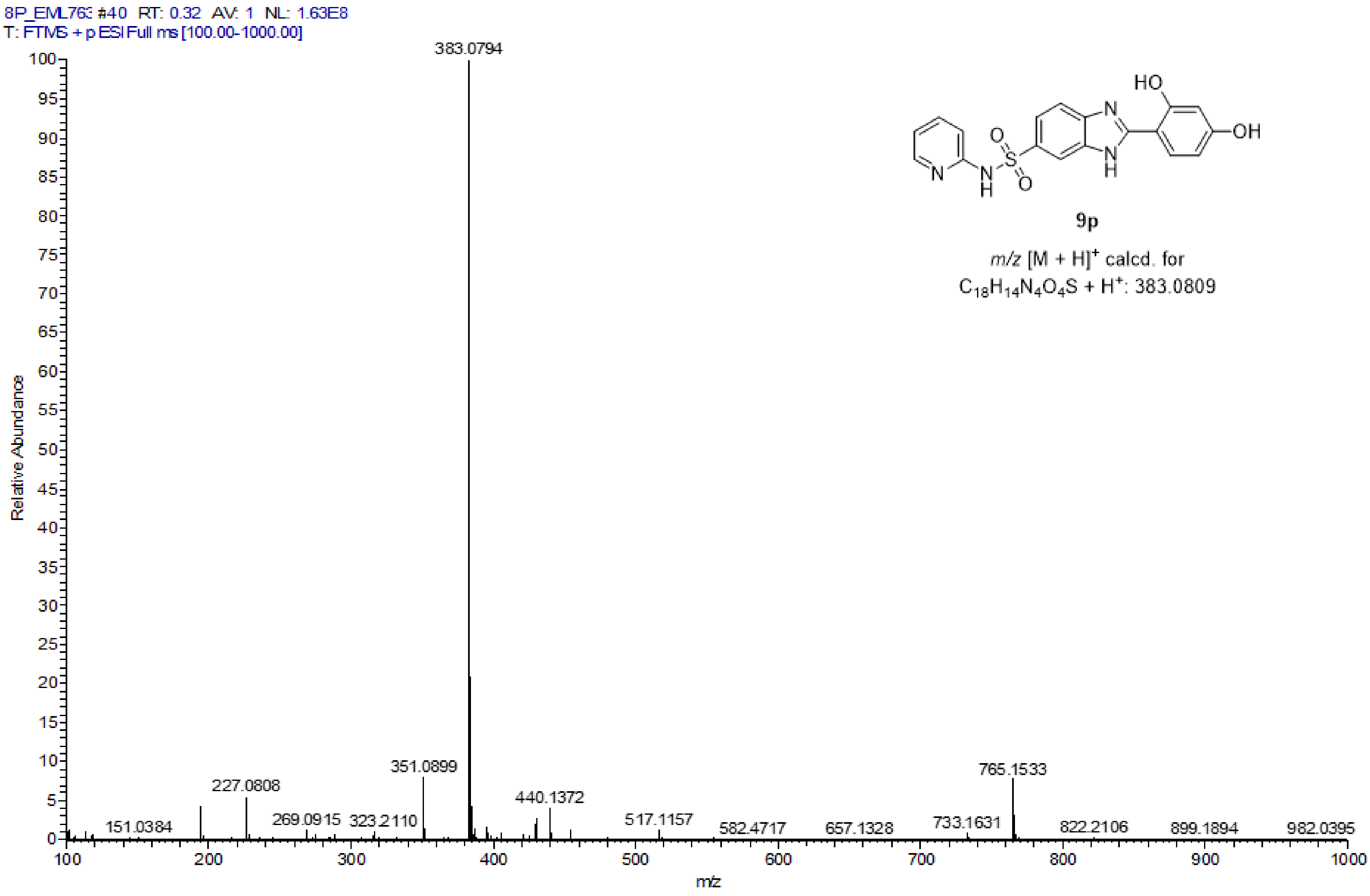

9p

m/z $[M + H]^+$ calcd. for $C_{18}H_{14}N_4O_4S + H^+$: 383.0809

HPLC traces of **9a**

SHIMADTZU HPLC ANALYSIS REPORT

Data File Name : 9a.lcd

Chromatogram 220 nm

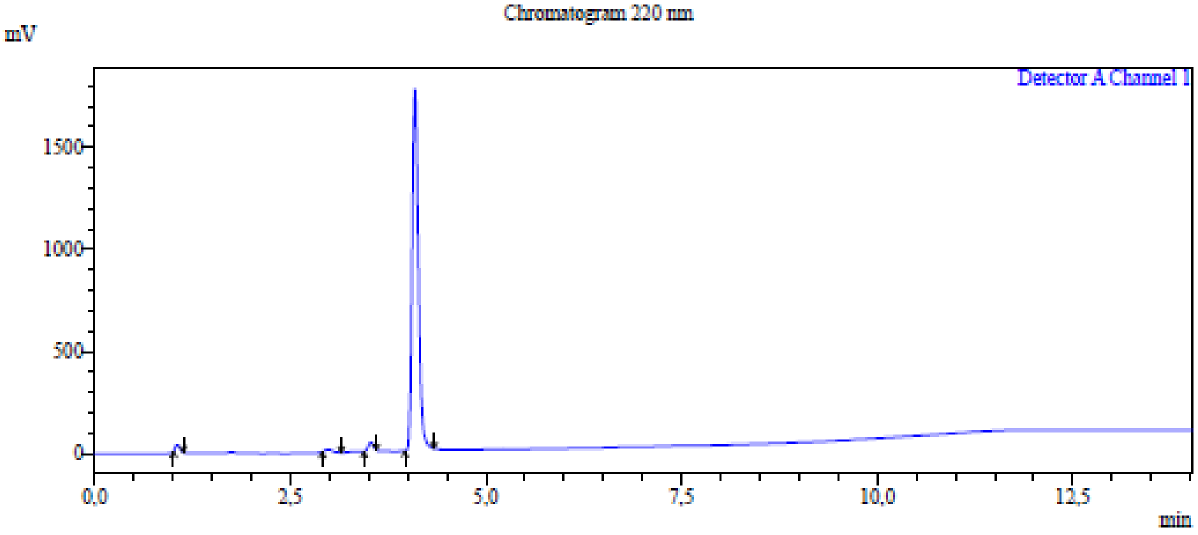

Peak Table

Detector A Channel 1 220nm

Peak#	Ret. Time	Height	Area	Area%
1	1,051	33117	131564	1,311
2	2,976	12986	77010	0,767
3	3,524	39416	178698	1,780
4	4,089	1769378	9650849	96,142
Total		1854896	10038120	100,000

Chromatogram 254 nm

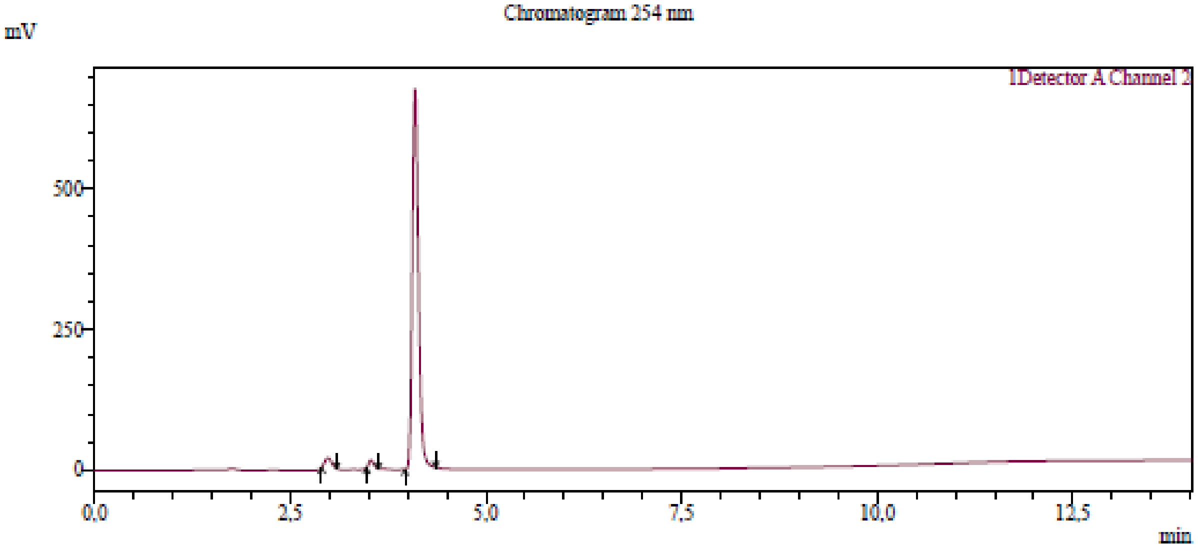

Peak Table

Detector A Channel 2 254nm

Peak#	Ret. Time	Height	Area	Area%
1	2,975	17866	106834	2,801
2	3,526	13552	59309	1,555
3	4,090	675095	3647669	95,644
Total		706513	3813812	100,000

HPLC traces of **9b**

SHIMADTZU HPLC ANALYSIS REPORT

Data File Name : 9b.lcd

Chromatogram 220 nm

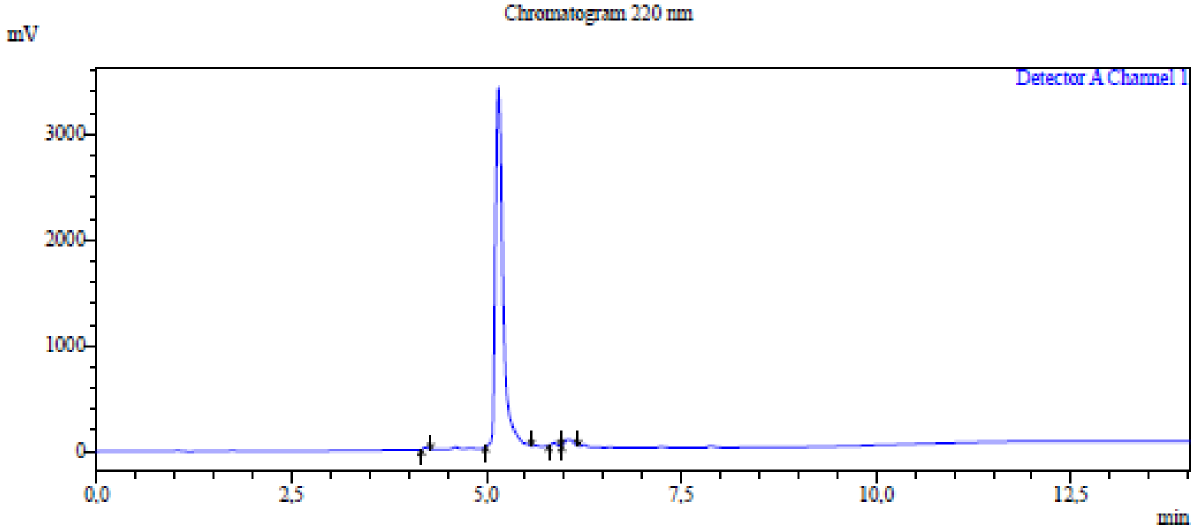

Peak Table

Detector A Channel 1 220nm

Peak#	Ret. Time	Height	Area	Area%
1	4,216	17997	70489	0,299
2	5,151	3386268	23006903	97,485
3	5,860	26800	111581	0,473
4	6,042	58079	411416	1,743
Total		3489144	23600389	100,000

Chromatogram 254 nm

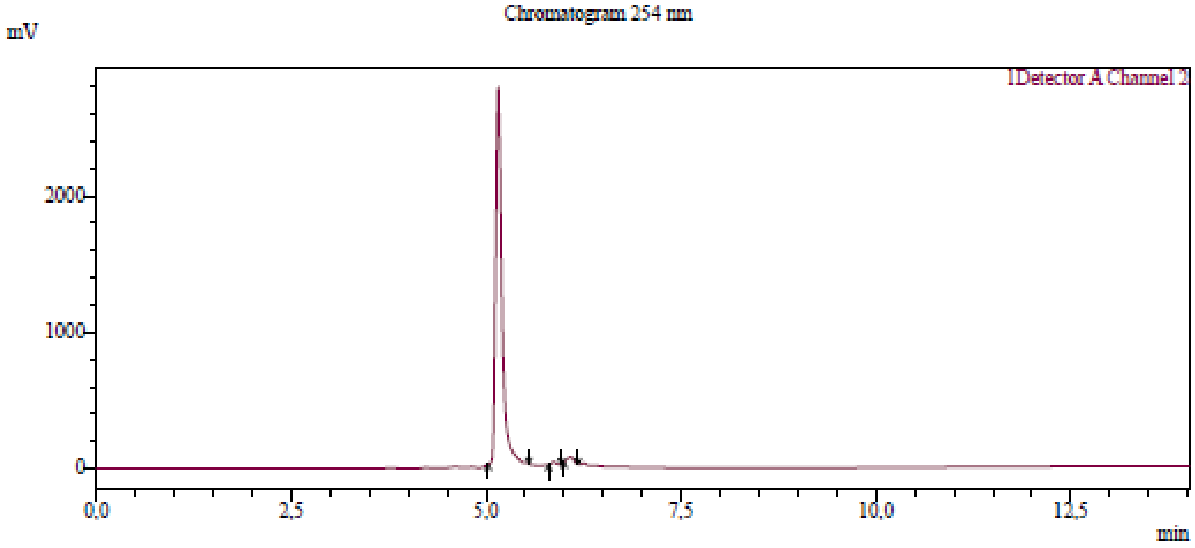

Peak Table

Detector A Channel 2 254nm

Peak#	Ret. Time	Height	Area	Area%
1	5,150	2758959	15629586	97,885
2	5,860	20312	83199	0,521
3	6,081	45023	254523	1,594
Total		2824294	15967308	100,000

HPLC traces of **9c**

SHIMADTZU HPLC ANALYSIS REPORT

Data File Name : 9c.lcd

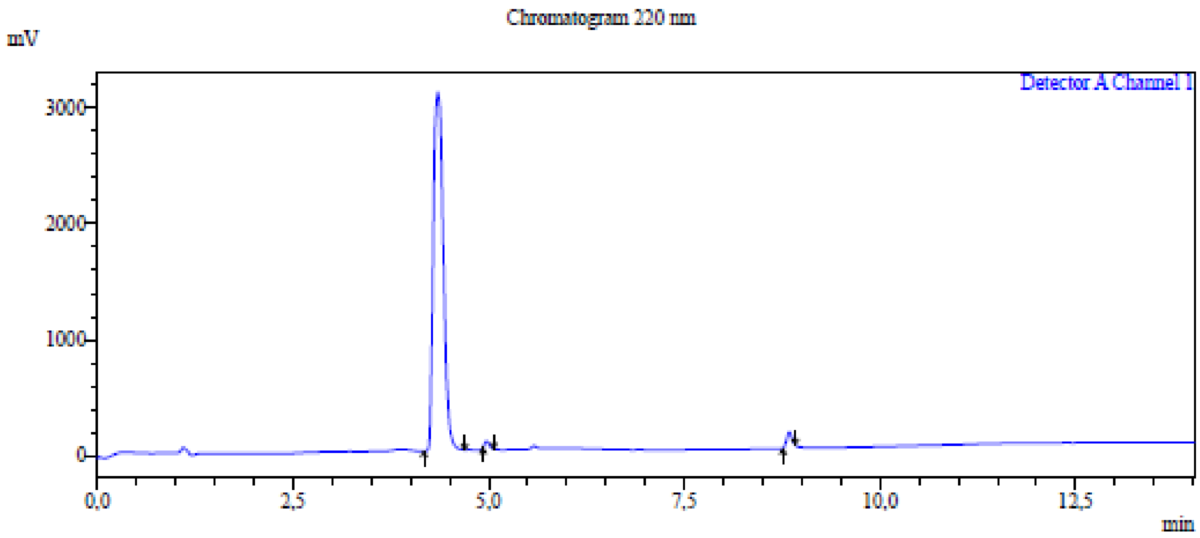

Peak Table

Detector A Channel 1 220nm

Peak#	Ret. Time	Height	Area	Area%
1	4,347	3079413	27546681	97,145
2	4,972	59588	250804	0,884
3	8,835	124158	558682	1,970
Total		3263159	28356167	100,000

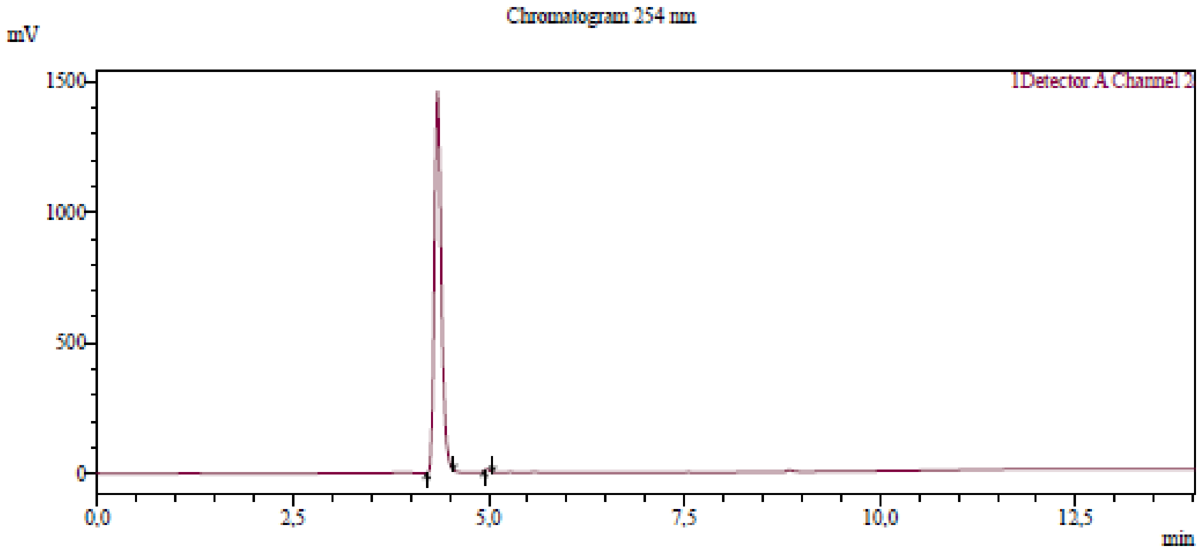

Peak Table

Detector A Channel 2 254nm

Peak#	Ret. Time	Height	Area	Area%
1	4,342	1454730	9031112	99,691
2	4,973	8058	28037	0,309
Total		1462788	9059149	100,000

HPLC traces of **9d**

SHIMADTZU HPLC ANALYSIS REPORT

Data File Name : 9d.lcd

Chromatogram 220 nm

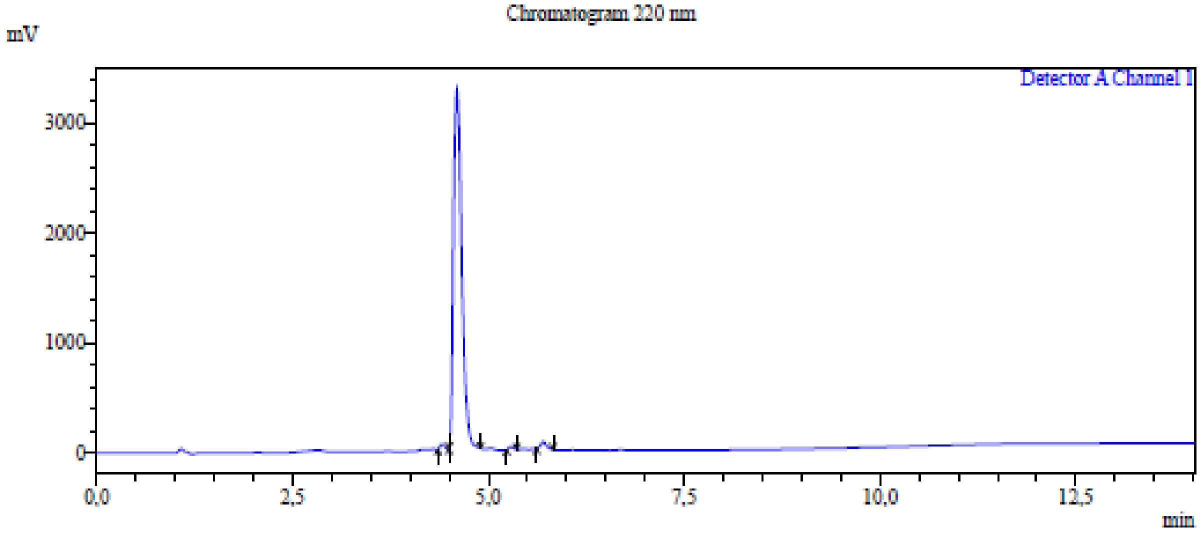

Peak Table

Detector A Channel 1 220nm

Peak#	Ret. Time	Height	Area	Area%
1	4,408	41405	143761	0,591
2	4,595	3268500	23753726	97,662
3	5,289	24918	104063	0,428
4	5,696	63523	320720	1,319
Total		3398347	24322270	100,000

Chromatogram 254 nm

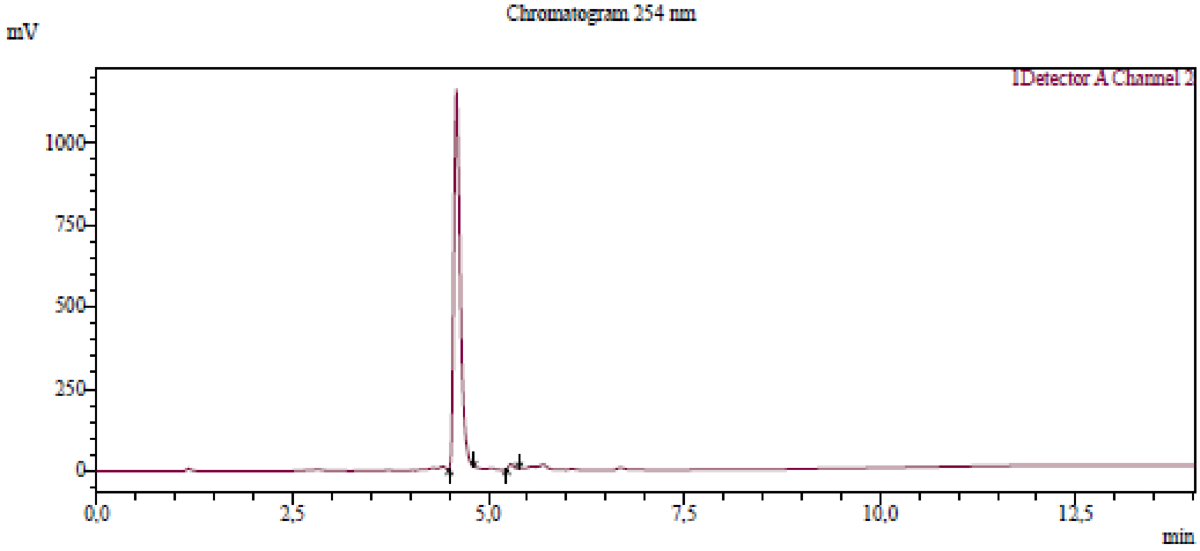

Peak Table

Detector A Channel 2 254nm

Peak#	Ret. Time	Height	Area	Area%
1	4,589	1154286	6490078	99,125
2	5,287	13246	57271	0,875
Total		1167532	6547349	100,000

HPLC traces of **9e**

SHIMADTZU HPLC ANALYSIS REPORT

Data File Name : 9e.lcd

Chromatogram 220 nm

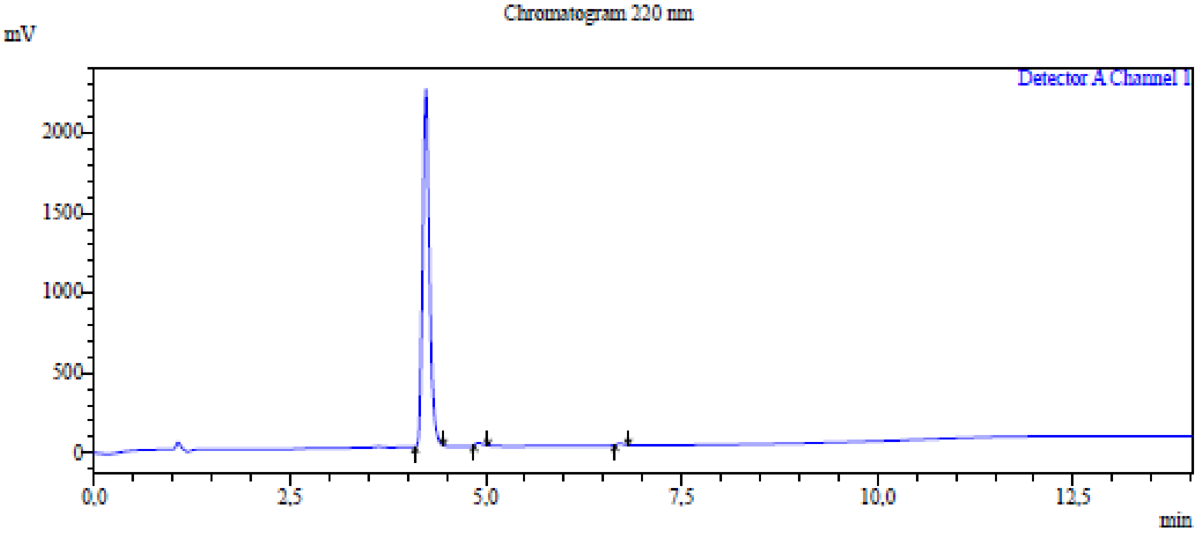

Peak Table

Detector A Channel 1 220nm

Peak#	Ret. Time	Height	Area	Area%
1	4,229	2236517	13639886	98,960
2	4,901	17641	89022	0,646
3	6,708	10795	54294	0,394
Total		2264954	13783202	100,000

Chromatogram 254 nm

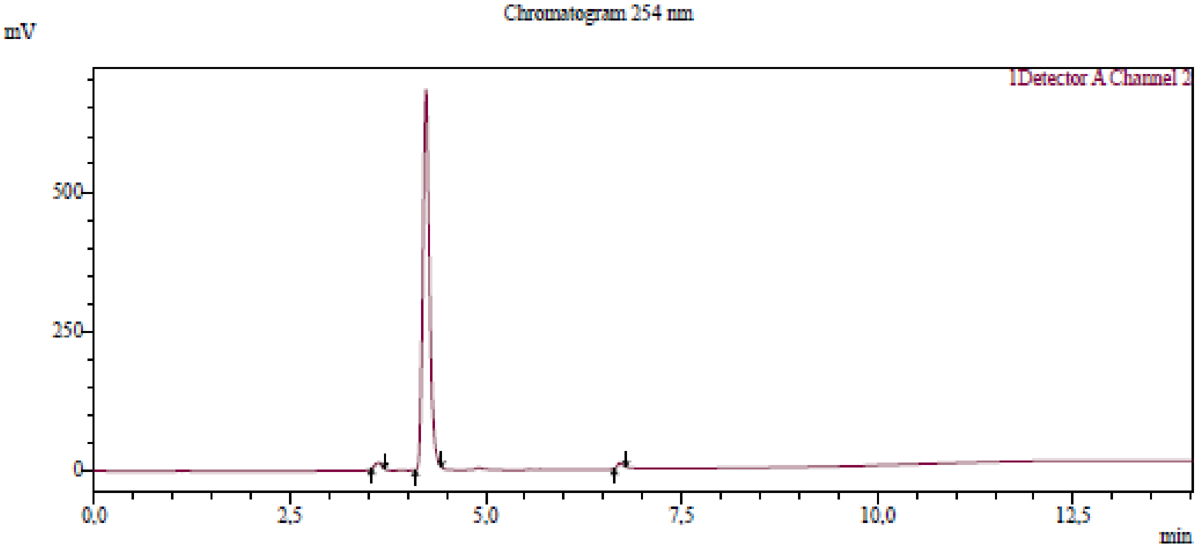

Peak Table

Detector A Channel 2 254nm

Peak#	Ret. Time	Height	Area	Area%
1	3,628	10954	61410	1,482
2	4,231	681238	4043733	97,573
3	6,710	8253	39186	0,946
Total		700445	4144329	100,000

HPLC traces of **9f**

SHIMADTZU HPLC ANALYSIS REPORT

Data File Name : 9f.lcd

Chromatogram 220 nm

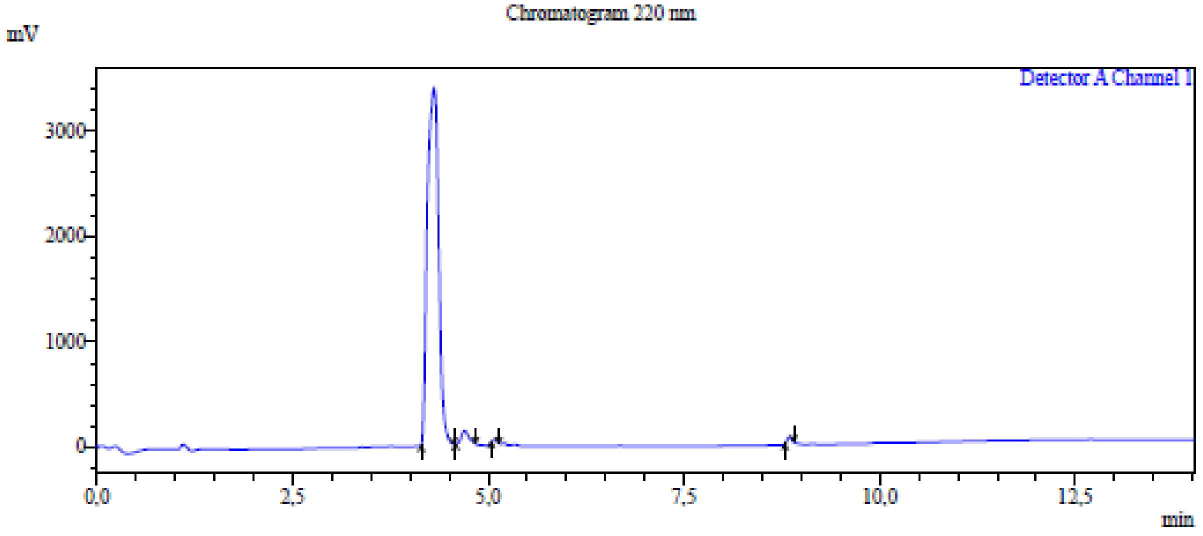

Peak Table

Detector A Channel 1 220nm

Peak#	Ret. Time	Height	Area	Area%
1	4,303	3377695	33989208	96,843
2	4,691	125023	802847	2,287
3	5,076	30748	100188	0,285
4	8,846	55045	204997	0,584
Total		3588510	35097240	100,000

Chromatogram 254 nm

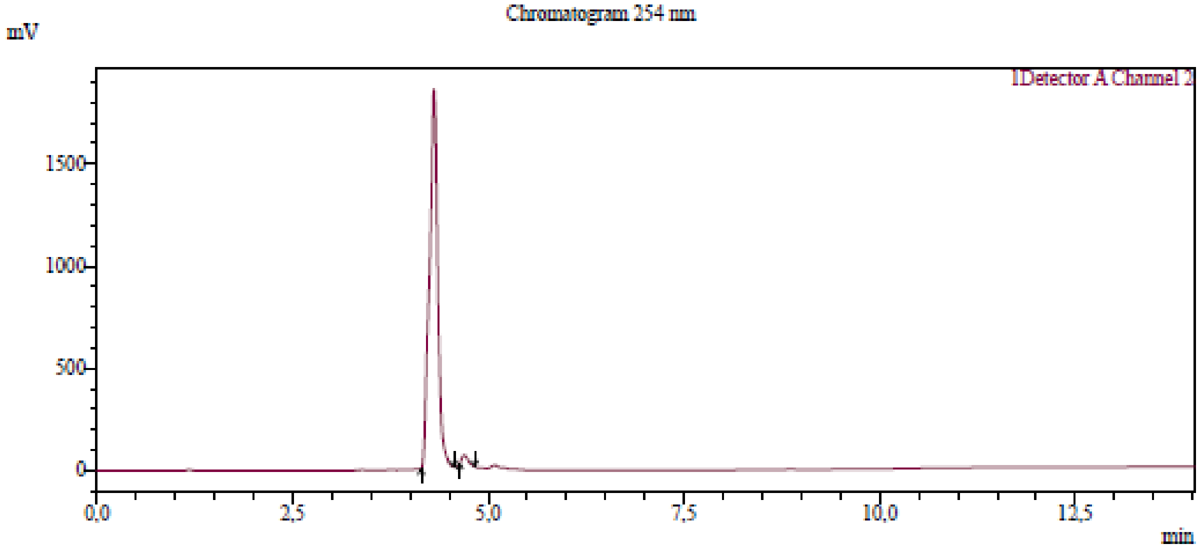

Peak Table

Detector A Channel 2 254nm

Peak#	Ret. Time	Height	Area	Area%
1	4,303	1852582	13573715	98,211
2	4,688	45974	247320	1,789
Total		1898556	13821035	100,000

HPLC traces of **9g**

SHIMADTZU HPLC ANALYSIS REPORT

Data File Name : 9g.lcd

Chromatogram 220 nm

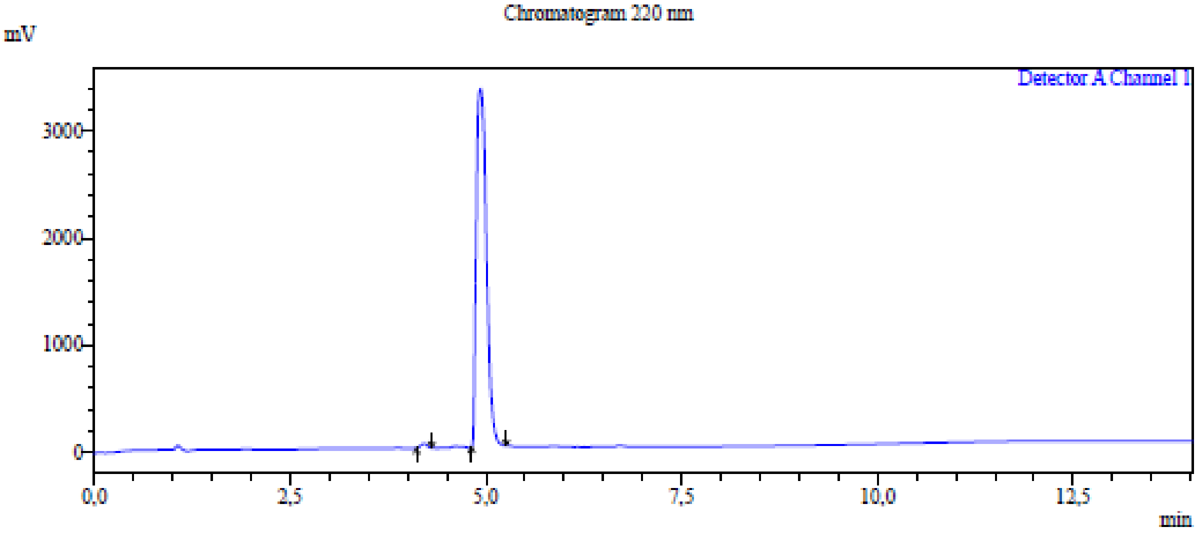

Peak Table

Detector A Channel 1 220nm

Peak#	Ret. Time	Height	Area	Area%
1	4,203	40446	213417	0,699
2	4,926	3342802	30306116	99,301
Total		3383248	30519533	100,000

Chromatogram 254 nm

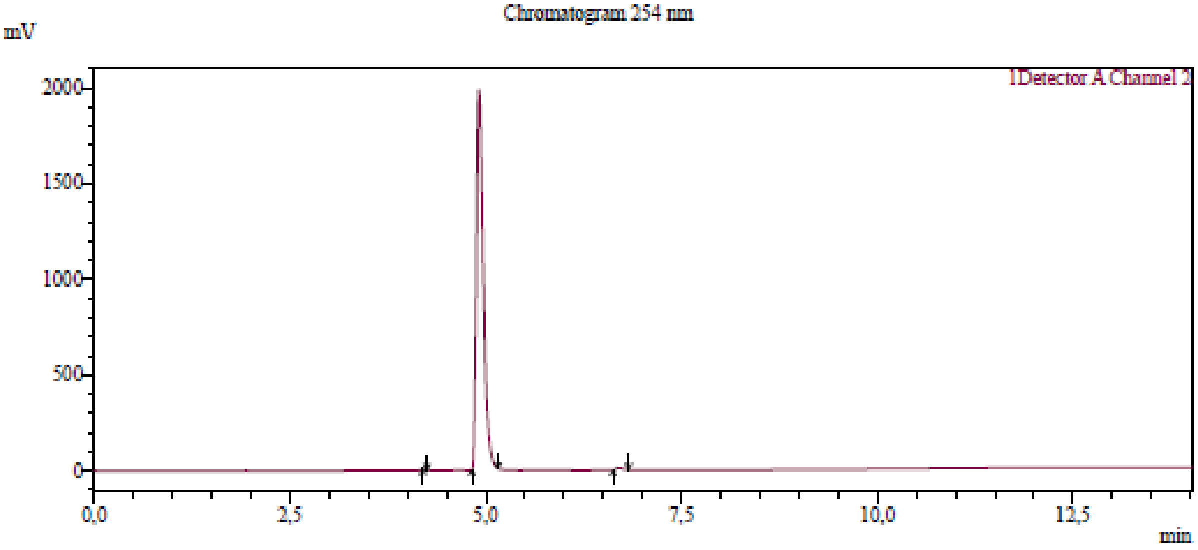

Peak Table

Detector A Channel 2 254nm

Peak#	Ret. Time	Height	Area	Area%
1	4,207	655	1433	0,012
2	4,912	1987165	12197337	99,538
3	6,702	10161	55124	0,450
Total		1997981	12253894	100,000

HPLC traces of **9h**

SHIMADTZU HPLC ANALYSIS REPORT

Data File Name : 9h.lcd

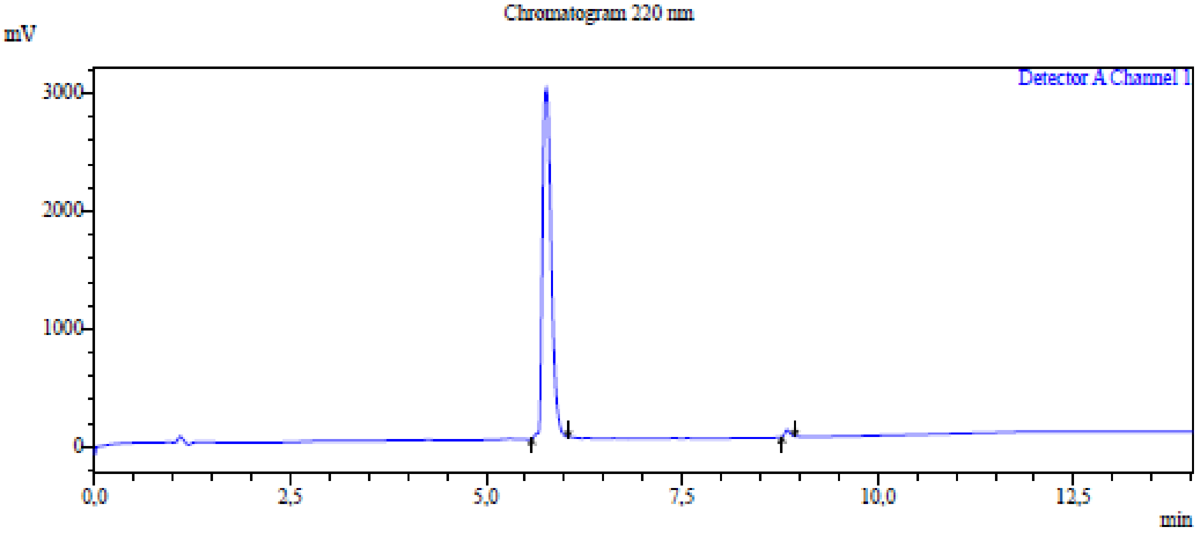

Peak Table

Detector A Channel 1 220nm

Peak#	Ret. Time	Height	Area	Area%
1	5,763	2968519	23225231	98,855
2	8,841	56209	268997	1,145
Total		3024727	23494229	100,000

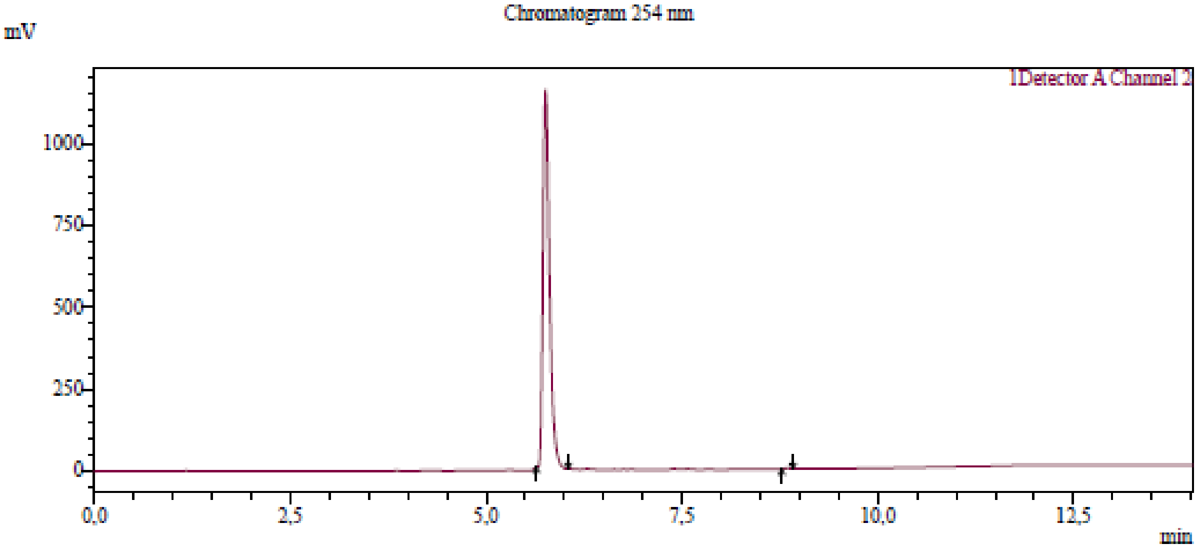

Peak Table

Detector A Channel 2 254nm

Peak#	Ret. Time	Height	Area	Area%
1	5,755	1154112	6753407	99,841
2	8,843	2442	10740	0,159
Total		1156554	6764148	100,000

HPLC traces of **9i**

SHIMADTZU HPLC ANALYSIS REPORT

Data File Name : 9i.lcd

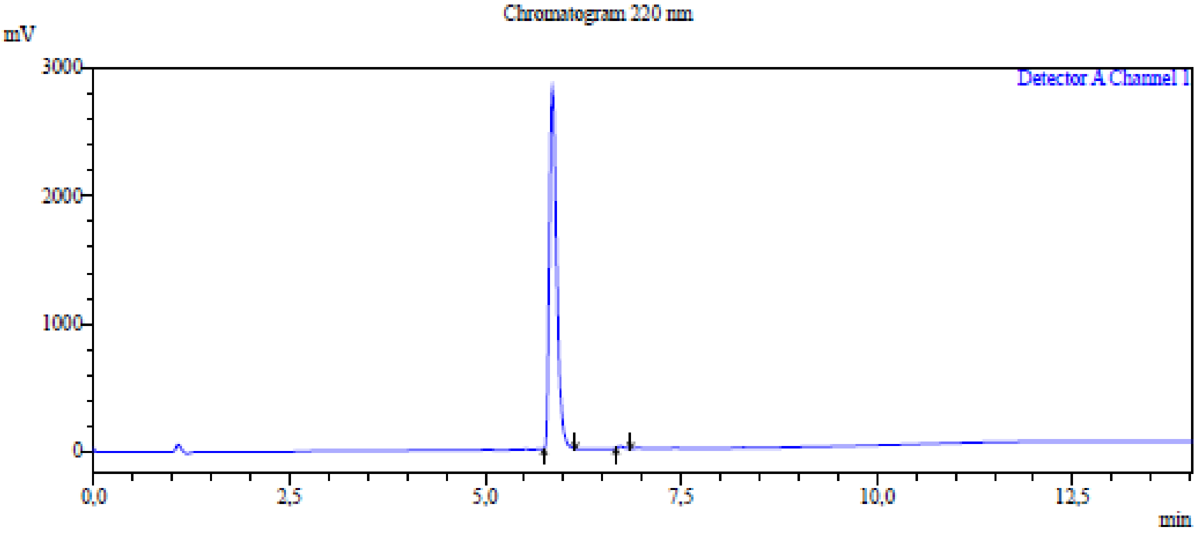

Peak Table

Detector A Channel 1 220nm

Peak#	Ret. Time	Height	Area	Area%
1	5,853	2815285	18166805	99,620
2	6,714	13421	69363	0,380
Total		2828707	18236168	100,000

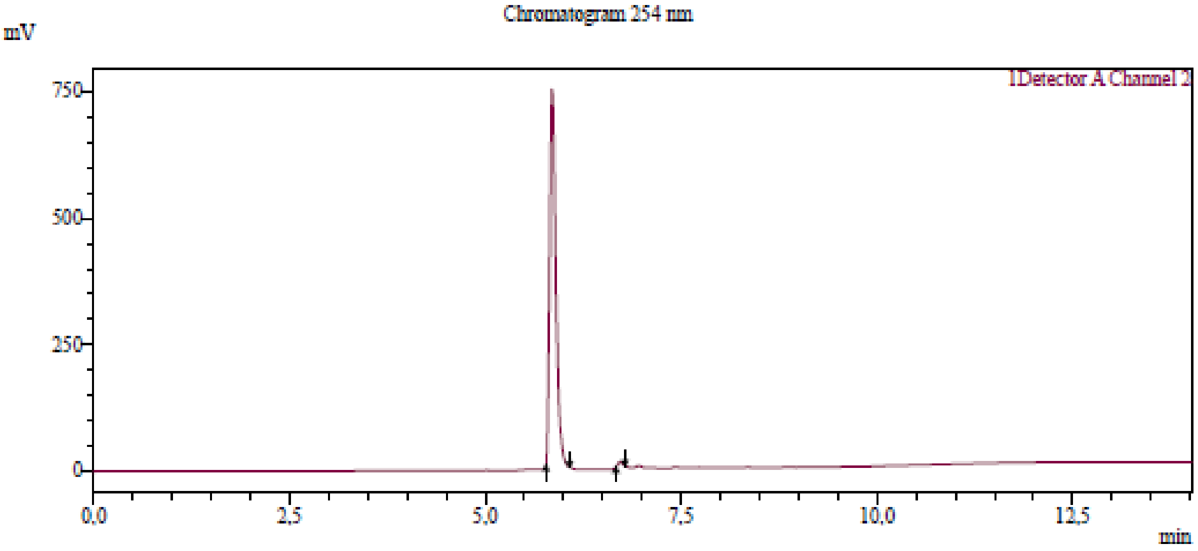

Peak Table

Detector A Channel 2 254nm

Peak#	Ret. Time	Height	Area	Area%
1	5,850	742645	4081445	99,126
2	6,716	8989	35991	0,874
Total		751634	4117436	100,000

HPLC traces of **9j**

SHIMADTZU HPLC ANALYSIS REPORT

Data File Name : 9j.lcd

Chromatogram 220 nm

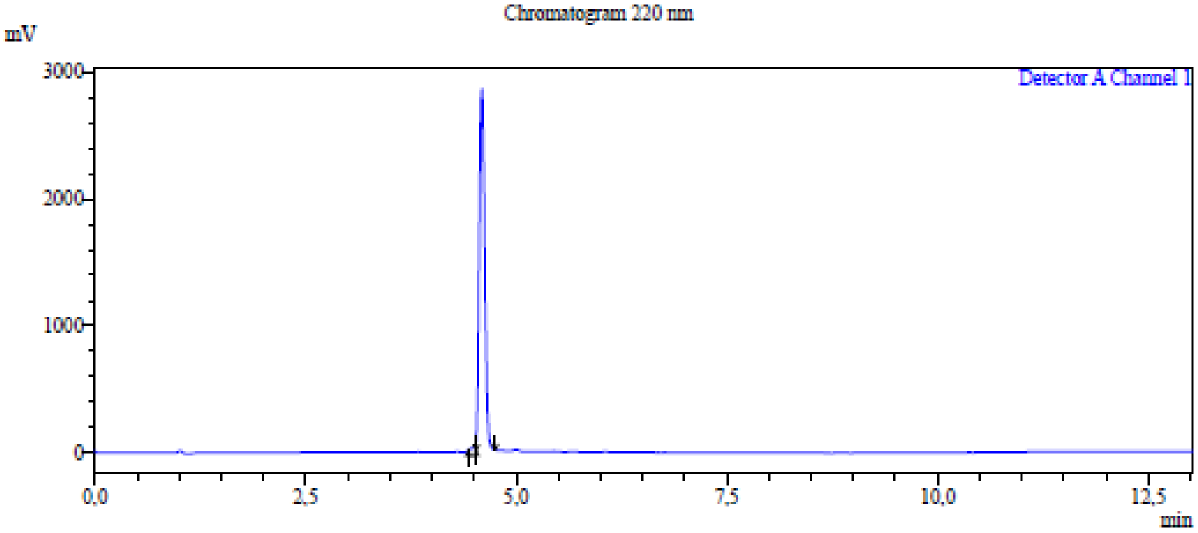

Peak Table

Detector A Channel 1 220nm

Peak#	Ret. Time	Height	Area	Area%
1	4,517	39112	143161	1,116
2	4,587	2850292	12680531	98,884
Total		2889404	12823691	100,000

Chromatogram 254 nm

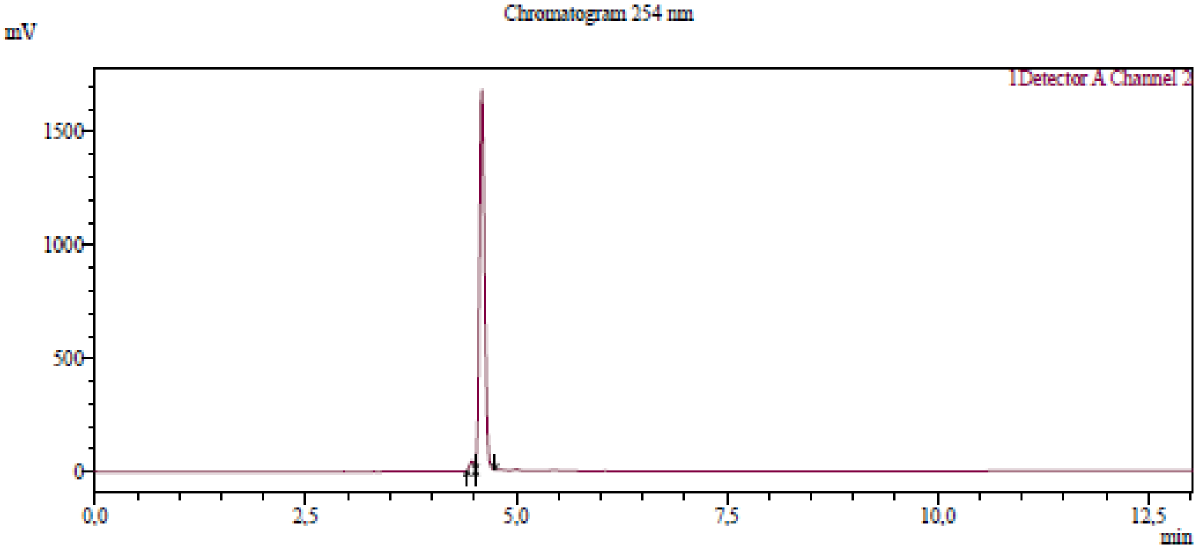

Peak Table

Detector A Channel 2 254nm

Peak#	Ret. Time	Height	Area	Area%
1	4,471	39358	154526	2,160
2	4,586	1679278	6999723	97,840
Total		1718635	7154249	100,000

HPLC traces of **9k**

SHIMADTZU HPLC ANALYSIS REPORT

Data File Name : 9k.lcd

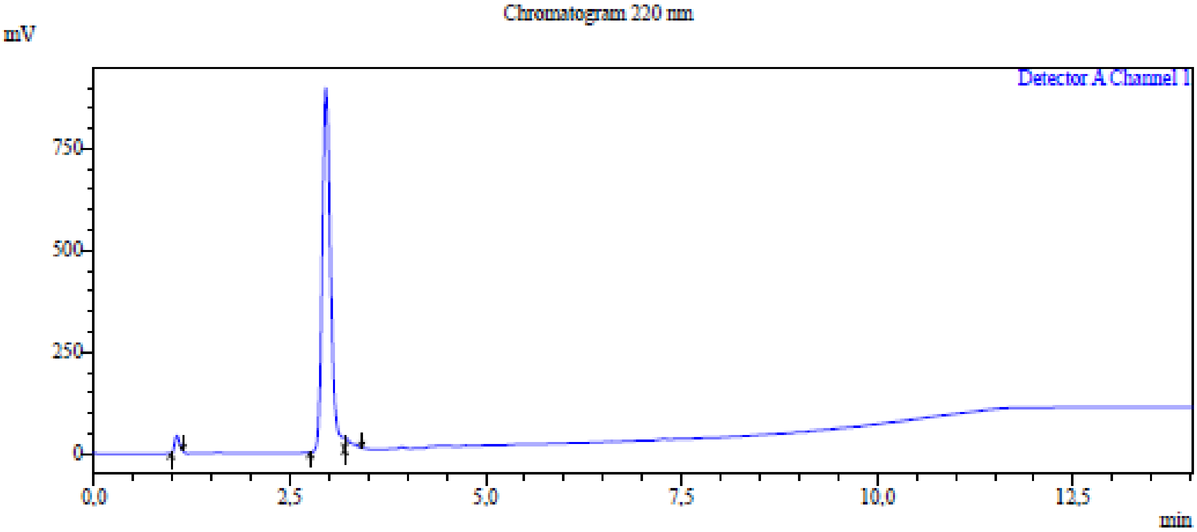

Peak Table

Detector A Channel 1 220nm

Peak#	Ret. Time	Height	Area	Area%
1	1,054	39241	163321	2,331
2	2,958	890402	6687870	95,465
3	3,200	28665	154416	2,204
Total		958309	7005607	100,000

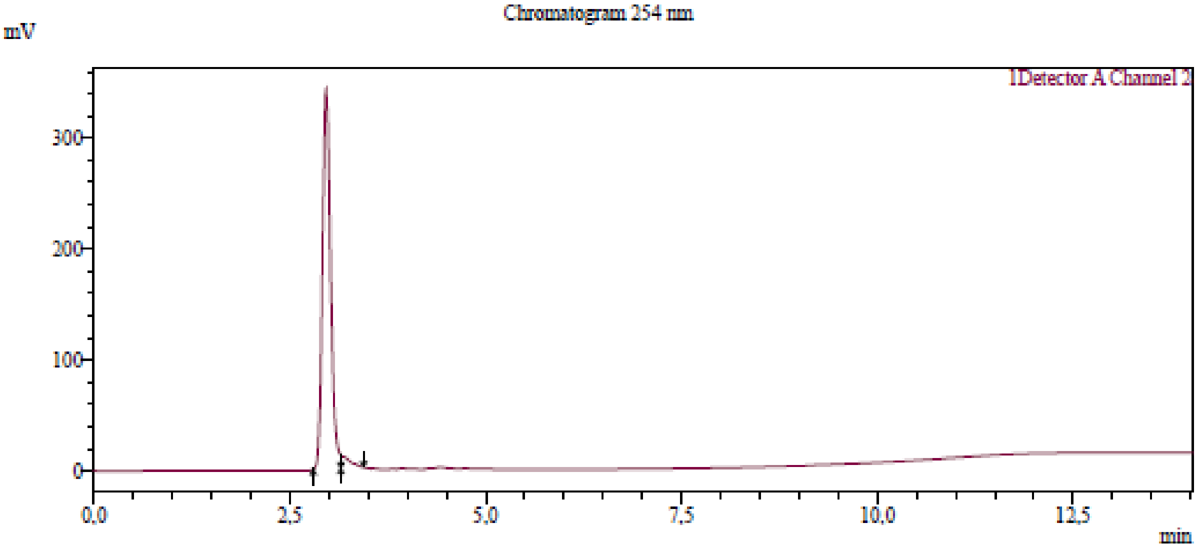

Peak Table

Detector A Channel 2 254nm

Peak#	Ret. Time	Height	Area	Area%
1	2,959	342127	2511994	96,750
2	3,158	10970	84373	3,250
Total		353097	2596367	100,000

HPLC traces of **9l**

SHIMADTZU HPLC ANALYSIS REPORT

Data File Name : 9l.lcd

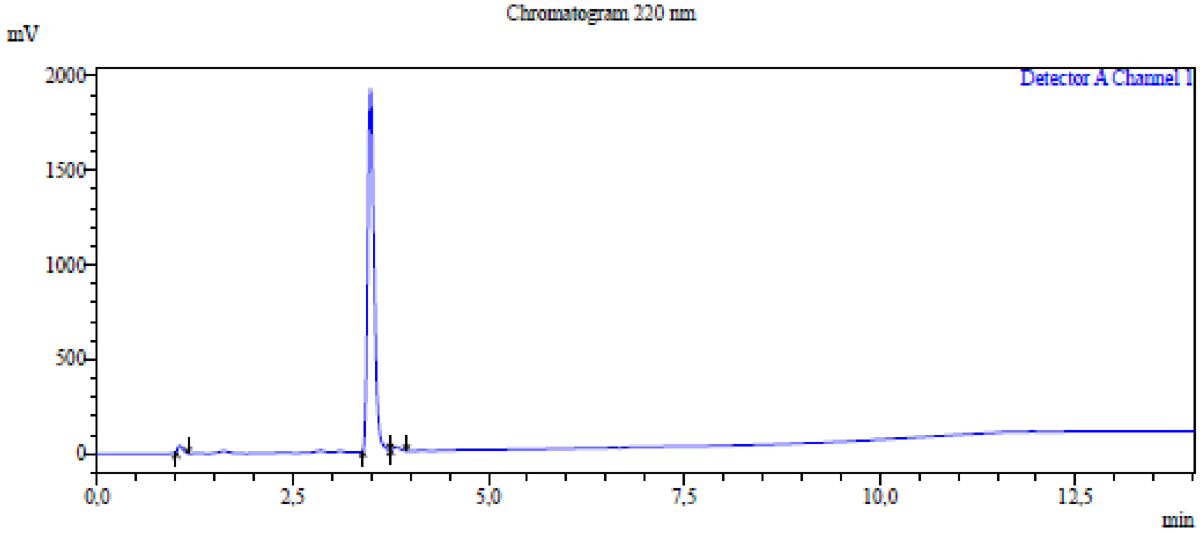

Peak Table

Detector A Channel 1 220nm

Peak#	Ret. Time	Height	Area	Area%
1	1,051	35684	154824	1,403
2	3,488	1917483	10795214	97,818
3	3,818	15955	85990	0,779
Total		1969122	11036028	100,000

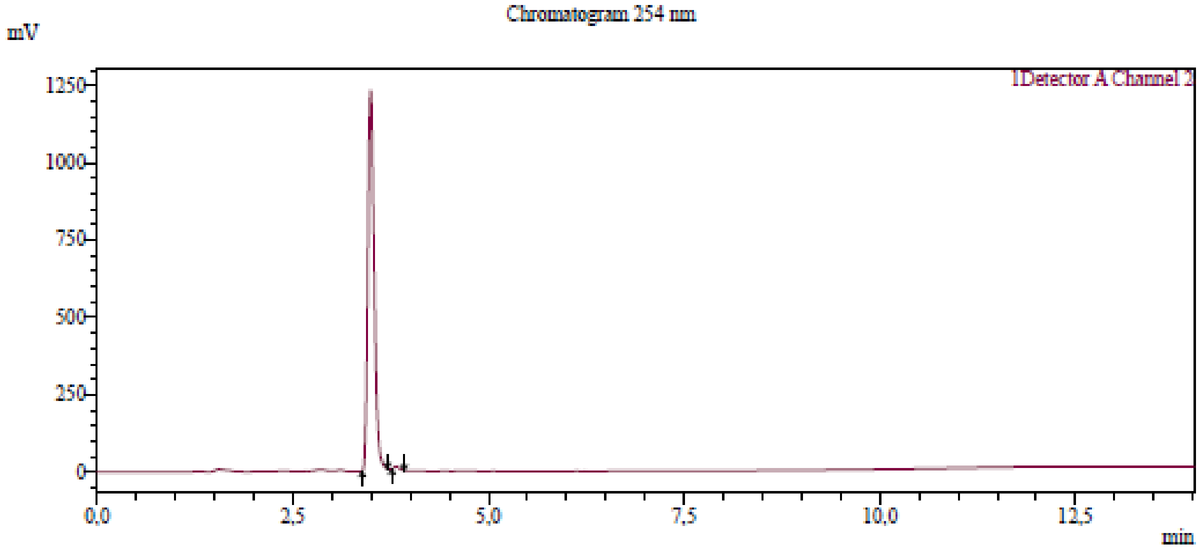

Peak Table

Detector A Channel 2 254nm

Peak#	Ret. Time	Height	Area	Area%
1	3,489	1231130	6905990	99,386
2	3,817	8712	42664	0,614
Total		1239842	6948654	100,000

HPLC traces of **9m**

SHIMADTZU HPLC ANALYSIS REPORT

Data File Name : 9m.lcd

Chromatogram 220 nm

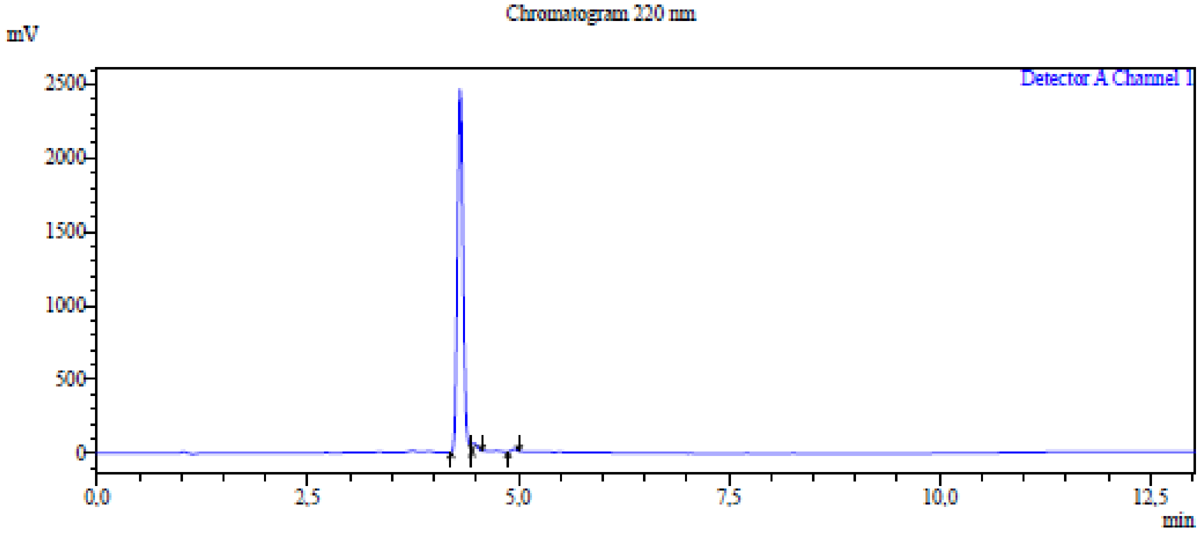

Peak Table

Detector A Channel 1 220nm

Peak#	Ret. Time	Height	Area	Area%
1	4,305	2459205	11508429	97,744
2	4,474	50796	204173	1,734
3	4,948	15401	61470	0,522
Total		2525402	11774072	100,000

Chromatogram 254 nm

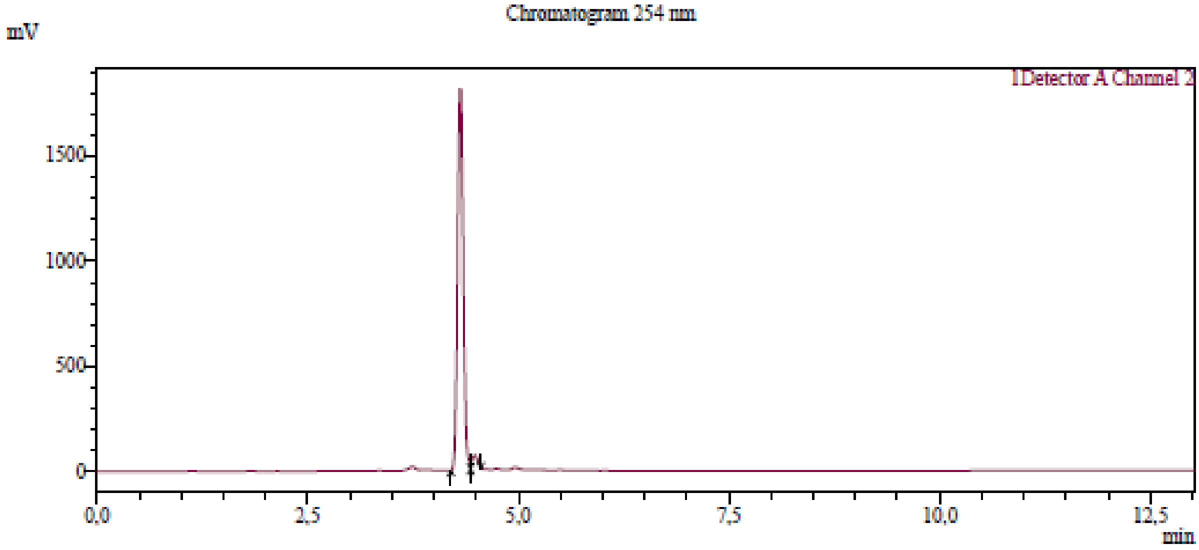

Peak Table

Detector A Channel 2 254nm

Peak#	Ret. Time	Height	Area	Area%
1	4,308	1811651	8491301	96,750
2	4,481	63556	285223	3,250
Total		1875207	8776524	100,000

HPLC traces of **9n**

SHIMADTZU HPLC ANALYSIS REPORT

Data File Name : 9n.lcd

Chromatogram 220 nm

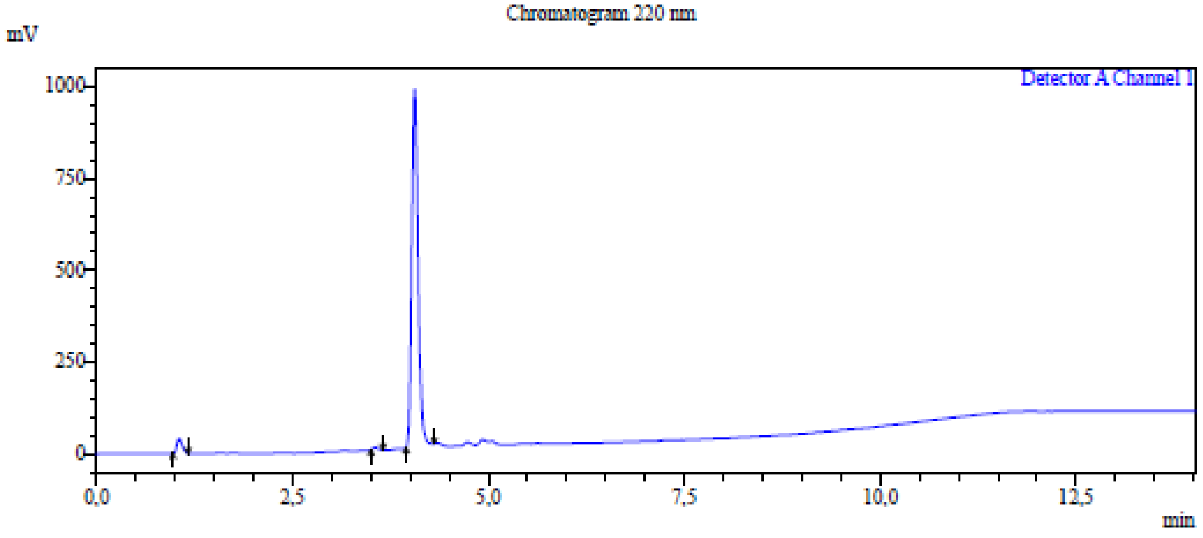

Peak Table

Detector A Channel 1 220nm

Peak#	Ret. Time	Height	Area	Area%
1	1,050	39573	191193	3,381
2	3,549	6090	26712	0,472
3	4,054	974828	5437845	96,147
Total		1020492	5655750	100,000

Chromatogram 254 nm

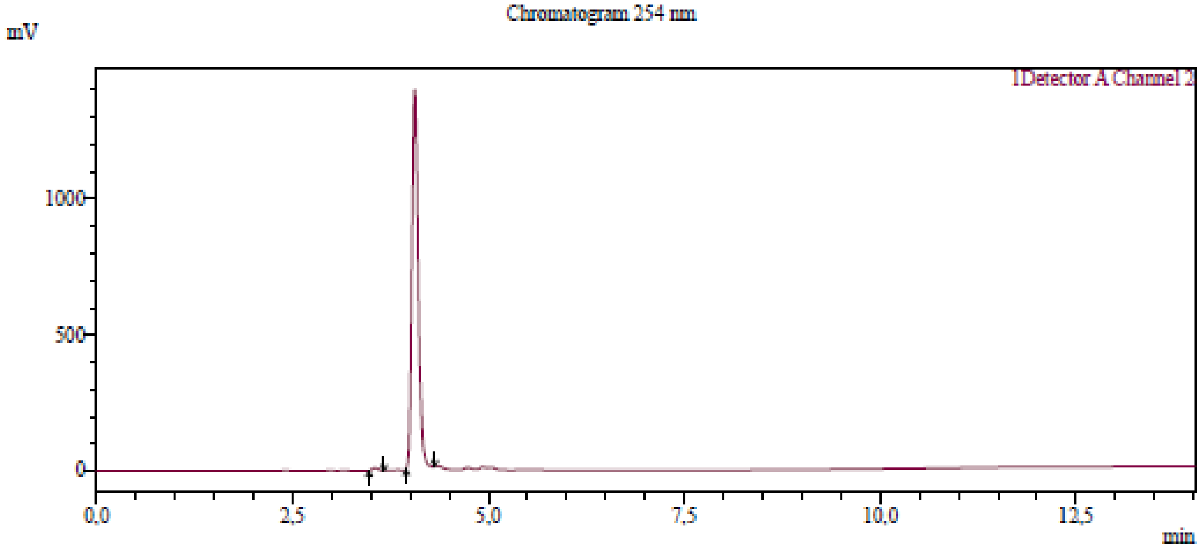

Peak Table

Detector A Channel 2 254nm

Peak#	Ret. Time	Height	Area	Area%
1	3,550	10673	54610	0,702
2	4,056	1391841	7725783	99,298
Total		1402514	7780393	100,000

HPLC traces of **9o**

SHIMADTZU HPLC ANALYSIS REPORT

Data File Name : 9o.lcd

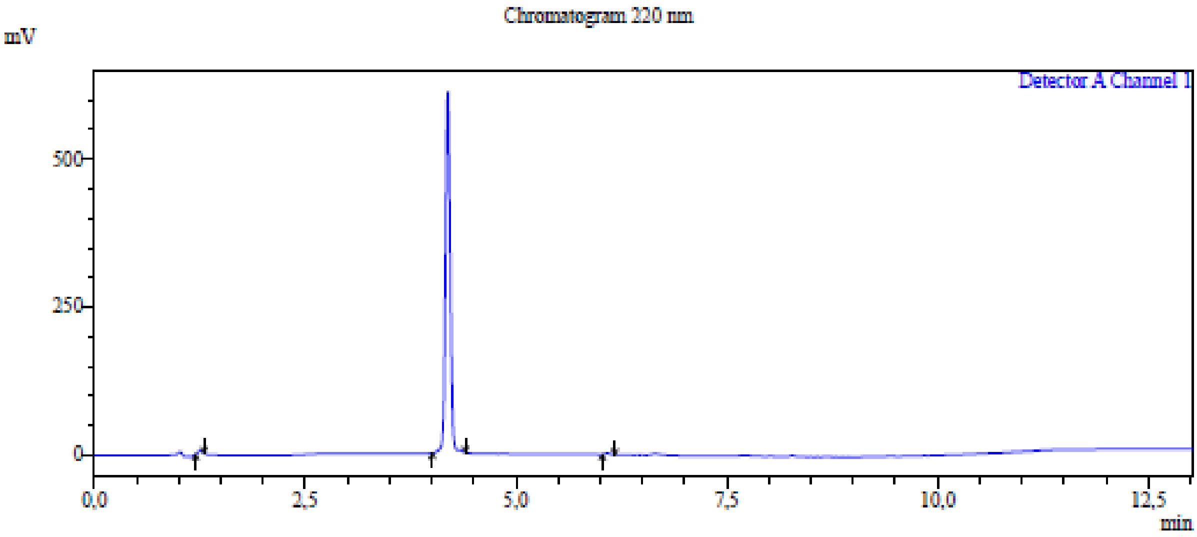

Peak Table

Detector A Channel 1 220nm

Peak#	Ret. Time	Height	Area	Area%
1	1,256	4674	17074	0,708
2	4,186	610468	2386253	98,938
3	6,079	2317	8550	0,354
Total		617459	2411877	100,000

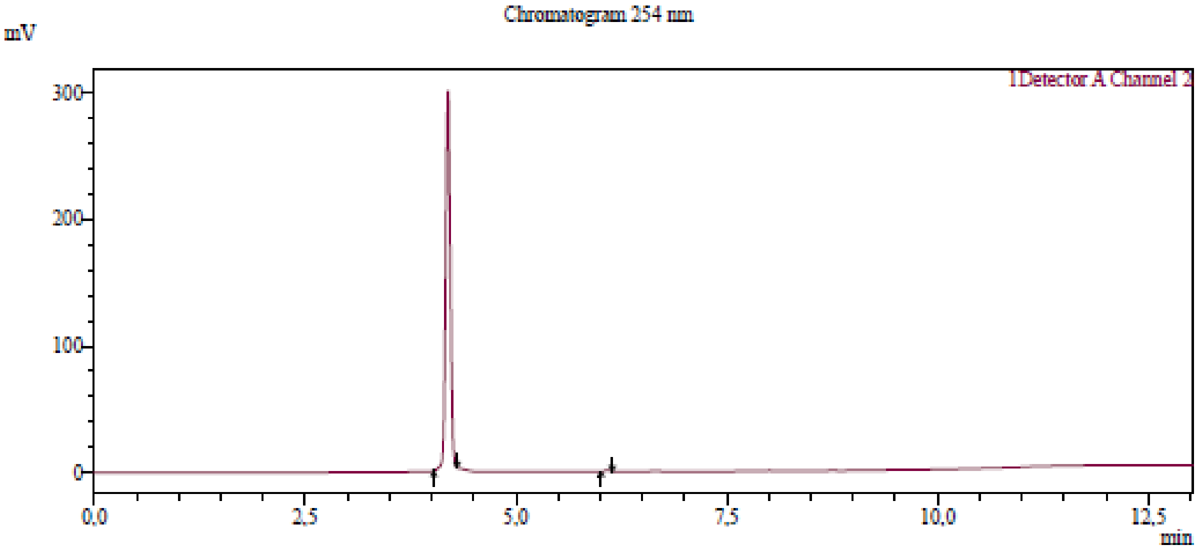

Peak Table

Detector A Channel 2 254nm

Peak#	Ret. Time	Height	Area	Area%
1	4,190	298864	1142505	99,653
2	6,079	1035	3984	0,347
Total		299899	1146489	100,000

HPLC traces of **9p**

SHIMADTZU HPLC ANALYSIS REPORT

Data File Name : 9p.lcd

Chromatogram 220 nm

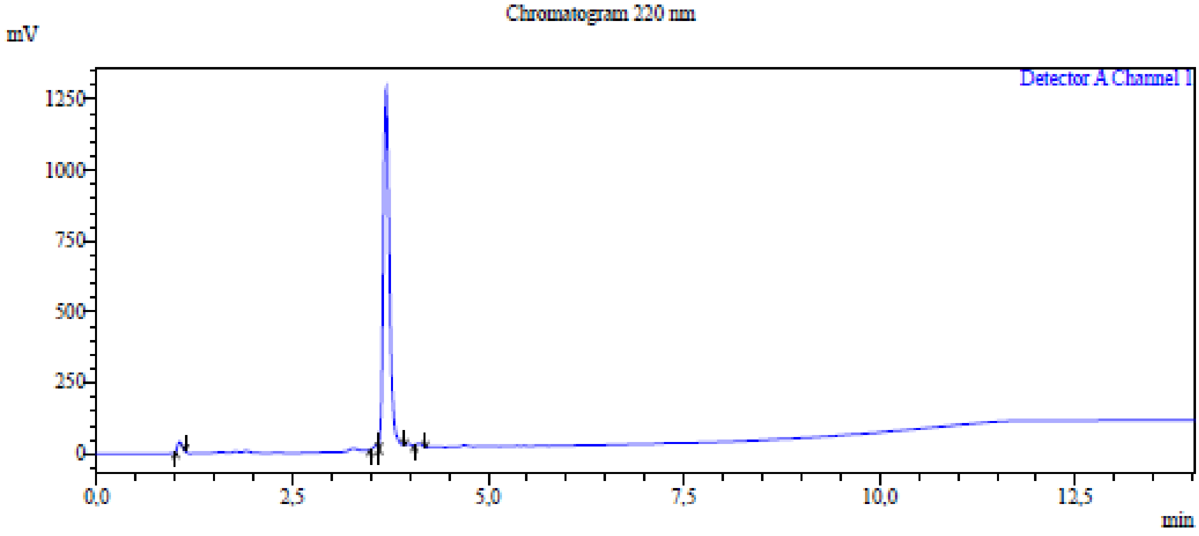

Peak Table

Detector A Channel 1 220nm

Peak#	Ret. Time	Height	Area	Area%
1	1,058	35002	139106	1,961
2	3,600	13243	64279	0,906
3	3,693	1265966	6847955	96,533
4	4,108	10220	42553	0,600
Total		1324430	7093892	100,000

Chromatogram 254 nm

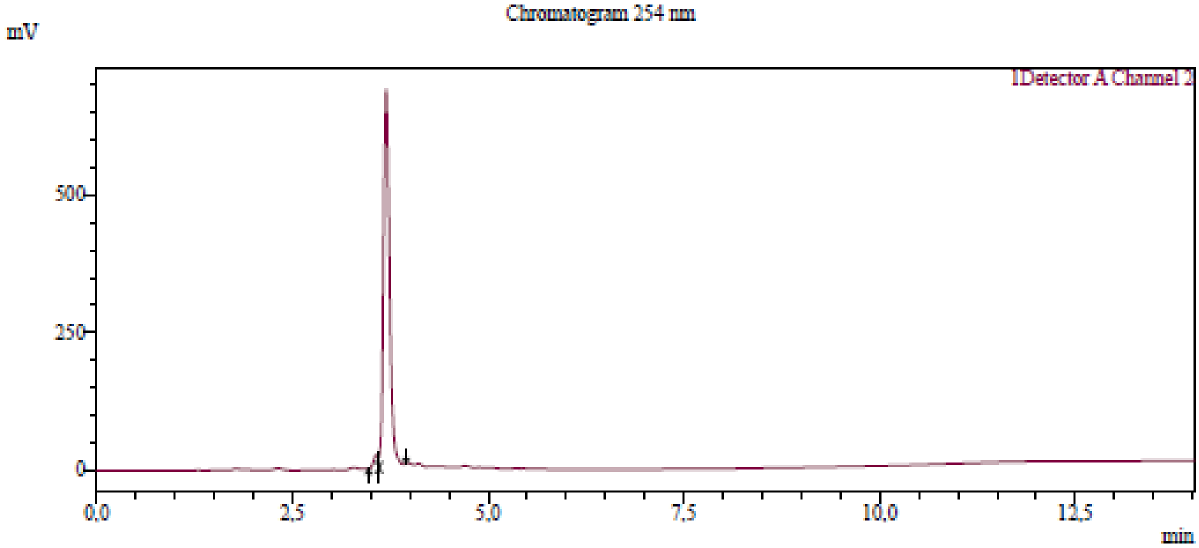

Peak Table

Detector A Channel 2 254nm

Peak#	Ret. Time	Height	Area	Area%
1	3,569	23055	104858	2,769
2	3,694	684383	3681838	97,231
Total		707437	3786695	100,000
